# Increased risk of multisystem comorbidities and disease trajectories following hyperthyroidism: evidence from the 0.5 million UK Biobank population

**DOI:** 10.1530/EC-25-0066

**Published:** 2025-04-23

**Authors:** Qiuyuan Chen, Doudou Chen, Xinpan Wang, Yunhao Zheng, Longyao Zhang, Zaiming Li, Xiaoyu Wu, Qin Chen, Ruyang Zhang, Feng Chen, Tao Yang, Xuqin Zheng, Yongyue Wei

**Affiliations:** ^1^ Department of Biostatistics, School of Public Health, Nanjing Medical University, Nanjing, China; ^2^ Department of Endocrinology, The Second Hospital of Nanjing, Nanjing, China; ^3^ Department of Endocrinology and Metabolism, The First Affiliated Hospital with Nanjing Medical University, Nanjing, China; ^4^ West China Medical Centre, Sichuan University, Chengdu, China; ^5^ Department of Epidemiology and Biostatistics, School of Public Health, Tongji Medical College, Huazhong University of Science and Technology, Wuhan, China; ^6^ The First School of Clinical Medicine, Nanjing Medical University, Nanjing, China; ^7^ Department of Public Health, Ningbo No 2 Hospital, Ningbo, China; ^8^ Centre for Public Health and Epidemic Preparedness and Response, Peking University, Beijing, China; ^9^ Department of Epidemiology and Biostatistics, School of Public Health, Peking University, Beijing, China; ^10^ Key Laboratory of Epidemiology of Major Diseases (Peking University), Ministry of Education, Beijing, China

**Keywords:** hyperthyroidism, disease trajectory, PheWAS, UK Biobank

## Abstract

**Background and aims:**

Hyperthyroidism is a clinical syndrome caused by the excessive production of thyroid hormones, which can have a broad impact on overall health. We systematically investigated the subsequent multisystem comorbidities associated with hyperthyroidism and the progression of these conditions.

**Methods:**

After a 1:4 propensity score matching, a total of 5,832 hyperthyroidism patients and 22,579 controls from the UK Biobank were included in this study. Phenome-wide association study was conducted to explore the associations between hyperthyroidism and a broad range of subsequent diseases, supplemented by landmark analysis to depict the time-varying effects. Disease trajectory analysis was used to explore the sequential pattern of comorbidity progression of hyperthyroidism.

**Results:**

Patients with prior diagnosed hyperthyroidism were observed to have an elevated risk of developing 110 subsequent diseases across multiple systems and all-cause mortality and four causes of death, with particularly marked short-term adverse effects. Disease trajectory analysis demonstrated that the three disease clusters most affected by hyperthyroidism were cardiovascular disease cluster, gastrointestinal inflammatory disease cluster and diabetes-mediated disease cluster.

**Conclusion:**

Hyperthyroidism is associated with an elevated risk of subsequent multisystem diseases and mortality. Disease trajectory analysis has elucidated critical sequential patterns of disease progression, offering valuable insights for the management of comorbidities in patients with hyperthyroidism.

## Introduction

Hyperthyroidism, a clinical syndrome induced by the excessive production of thyroid hormones (THs) (1), is primarily caused by Graves’ disease, with other etiologies including toxic multinodular goiter and toxic adenoma (2). Patients often require long-term medication or surgery and regular follow-up due to the chronic nature of hyperthyroidism (3). Globally, hyperthyroidism affects approximately 2.5% of the population, with a prevalence of 2% in women and 0.5% in men (4).

THs play a crucial role in the metabolism, growth and development of most of the tissues in the body (5). As a result, hyperthyroidism has broad effects on the physiological and biochemical functions of multiple systems. It is now widely accepted that patients with hyperthyroidism are at increased risk of several cardiovascular diseases (6, 7), psychiatric disorders (8), osteoporosis (9, 10) and eye diseases (11). Some studies have also investigated less common complications of hyperthyroidism and found an elevated risk of dementia (12), hearing disorders (13) and breast cancer (14, 15) after a diagnosis of hyperthyroidism. Mendelian randomization studies indicate a causal link between hyperthyroidism and conditions such as atrial fibrillation (16), heart failure (17) and osteoporosis (18). However, these studies have predominantly been hypothesis-driven, lacking a systematic examination of the full spectrum of the impact of hyperthyroidism on subsequent diseases, and the progression of these conditions remains unclear. A comprehensive evaluation of the effects of hyperthyroidism could enhance our knowledge of its overall impact on health and offer recommendations for managing comorbidities in patients with hyperthyroidism.

Phenome-wide association study (PheWAS) is designed to identify a broad range of diseases associated with a specific trait (19). Landmark analysis is used to estimate time-varying effects (20). Disease trajectory analysis has been widely used to explore sequential patterns of disease progression (21, 22). This study, based on the UK Biobank, integrates these techniques to comprehensively assess the increased risk of comorbidities and mortality following a diagnosis of hyperthyroidism, and to uncover patterns of disease progression.

## Methods

### Study design

**Figure 1:**
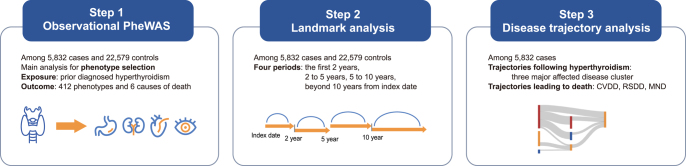
Flowchart of the study design.

In this study, PheWAS was first conducted to identify associations between prior diagnosed hyperthyroidism and subsequent diseases. Further analyses were based on the phenotypes identified in PheWAS. Landmark analysis was performed to investigate the short- and long-term effects of hyperthyroidism. Disease trajectory analysis was performed to elucidate the sequential pattern of diseases after hyperthyroidism and the pathways linking hyperthyroidism to specific causes of mortality. More details of the study design are presented in Fig. 1.

### Data material

UK Biobank is a large, population-based, prospective cohort study that recruited ∼500,000 participants aged 40–69 years between 2006 and 2010. Baseline assessments, including touch-screen questionnaires, interviews with trained nurses, physical examinations and biospecimen collection, were conducted for all participants at 22 assessment centers, collecting detailed information on socioeconomic and demographic characteristics, lifestyle factors, medical history and medication usage. Participants’ health-related outcomes were obtained through regular linkage to multiple national databases (e.g., inpatient medical records, cancer registries and death registries). Detailed information about the UK Biobank was described elsewhere (23, 24).

Ascertainment of hyperthyroidism, comorbidities and all-cause mortality diagnostic records used for this study were sourced from an integration of inpatient hospital data and self-reported records from the UK Biobank, which provided detailed information on types of diagnoses and timing of each diagnosis. Conditions related to pregnancy, childbirth, puerperium, congenital diseases and injuries or poisonings caused by external factors were not included in our analysis. ICD-10 codes in diagnostic records were mapped to PheCodes, a disease coding system designed for PheWAS, which defines different phenotypes by amalgamating multiple ICD codes that are clinically or biologically similar (25).

In the inpatient hospital data, diagnoses with ICD-10 codes E05.0∼E05.9 were identified as hyperthyroidism, corresponding to the PheCodes 242, 242.1 and 242.2. In the self-reported records, diagnoses coded 1225 (hyperthyroidism/thyrotoxicosis) and 1522 (Grave’s disease) were identified as hyperthyroidism. If a diagnosis was recorded multiple times for the same individual, only the first record and its corresponding timestamp were retained.

The time of death and the underlying cause of death were recorded in the UK Biobank mortality data. The underlying causes of death were categorized into 16 major causes of death based on affected systems (Supplementary Table S1 (see section on Supplementary materials given at the end of the article)).

### Statistical analysis

#### PheWAS

Participants with at least one recorded diagnosis of hyperthyroidism were identified. To ensure the completeness of diagnostic records following a hyperthyroidism diagnosis, only cases diagnosed after January 1, 1997 were included in this study, and those who died on the day of diagnosis of hyperthyroidism were excluded.

To achieve a relatively balanced case and control group, we conducted 1:4 propensity score matching (PSM) among individuals who had never been diagnosed with hyperthyroidism during follow-up. Factors considered during the matching process included year of birth, gender, baseline BMI, Townsend Deprivation Index, educational level, smoking status, alcohol consumption and physical activity levels. Details about PSM and matching covariates are provided in the Supplementary Methods.

The diagnosis date of hyperthyroidism for each case was designated as the index date for the matched group. Control group members who died or were lost to follow-up before the index date were excluded from analysis. In addition, to exclude possible underreporting in the diagnostic records, control individuals who have used hyperthyroidism medications in the baseline records or a history of thyroid surgery were removed from analysis. The medications and surgical procedures considered for exclusion included antithyroid drugs (including carbimazole and propylthiouracil) and thyroid surgery history (including thyroid radioablation therapy and thyroidectomy/partial thyroidectomy).

In this study, phenotypes that co-occurred with hyperthyroidism in at least 20 cases were selected as candidate phenotypes. Each matched group, comprising one case and multiple controls, was considered as a subcohort. For each subcohort, the index date was established as the date of entry into the cohort. Stratified Cox regression was used to examine the associations between prior diagnosed hyperthyroidism and each candidate phenotype. Follow-up concluded with the earliest of the following events: onset of the analyzed phenotype, death, loss to follow-up or the censoring date (October 31, 2022, for England, August 31, 2022, for Scotland, and May 31, 2022, for Wales). For each analysis, individuals who had previously been diagnosed with the analyzed phenotype prior to the index date were excluded.

Fine-Gray competing risk model was used to analyze the impact of hyperthyroidism on specific causes of death, taking into account the competing risks from different death causes. Only death causes occurring in at least 20 hyperthyroidism patients were included in the analysis.

#### Landmark analysis

Landmark analyses were conducted to investigate the short- and long-term effects of prior diagnosed hyperthyroidism. Analyses were divided into four periods: the first 2 years, 2–5 years, 5–10 years and beyond 10 years from the index date. The inception of each period was considered the cohort entry date, and the end of each period signaled the termination of observation. Hazard ratios were assessed for each period. For each landmark analysis, individuals who had already been diagnosed with the analyzed phenotype or who had died or were lost to follow-up before the landmark point were excluded.

#### Disease trajectory analysis

Methods for constructing disease trajectories have been detailed in previous studies (26, 27). In disease trajectories, arrows indicated the direction of progression between different phenotypes. The study population for this section was limited to patients diagnosed with hyperthyroidism.

First, among the phenotypes identified in PheWAS, all possible disease pairs D1 → D2 with at least ten cases were selected. Second, binomial test was used to evaluate the statistical significance of the sequential relationship of D1 → D2. The disease pair was selected for further analysis only if the proportion of D1 preceding D2 significantly exceeded 50% among individuals diagnosed with both D1 and D2. Third, with D1 as the exposure and D2 as the outcome, the effect of D1 on the risk of D2 was calculated, and disease pairs with statistically significant OR >1 were selected. Fourth, if the three disease pairs D1 → D2, D2 → D3 and D1 → D3 coexisted, mediation analyses were conducted with D2 serving as the mediator. The sequence D1 → D2 → D3 was then linked into a disease trajectory of length 3 if a statistically significant indirect effect of D2 was observed in the D1 → D2 → D3 pathway. Similarly, disease trajectories of longer length can be constructed by mediating the linkage of diseases.

A similar approach was applied to construct trajectories leading to mortality. For each cause of death, logistic regression was used to identify phenotypes significantly associated with it. These phenotypes were then used to construct the trajectories leading to each cause of death.

#### Subgroup and sensitivity analysis

To investigate the heterogeneity of impact across different population, the dataset was stratified by gender, and Cox regression was used to estimate the effects within each gender subgroup. Restricted cubic splines were used to characterize the moderating effect of age at diagnosis on these effects.

PheWAS was conducted with the exclusion of diagnostic records within 6 months after the index date to mitigate the impact of diagnostic delays. A subgroup with at least two recorded diagnoses of hyperthyroidism, spaced more than 3 months apart, was selected for analysis to identify cases of persistent hyperthyroidism. Certain thyroid disorders, such as thyroiditis, may induce temporary hyperthyroid symptoms; therefore, cases of hyperthyroidism with prior diagnoses of other thyroid disorders before the index date were excluded. Given that immunotherapy for cancer can significantly affect thyroid function, participants with any diagnosis of malignant neoplasms prior to the index date were excluded.

Bonferroni method was utilized to adjust for multiple testing. Adjusted *P* value of less than 0.05 was considered statistically significant. All analyses were conducted using the R Software version 4.2.3 (The R Project for Statistical Computing). The code and sample data are available for download at (https://github.com/daisy3608/HyperthyroidismComorbidities).

### Role of the funding source

The funder of the study had no role in study design, data collection, data analysis, data interpretation or writing of the report. The corresponding author had full access to all of the data and the final responsibility to submit for publication.

## Results

Among all participants from the UK Biobank, a total of 7,955 individuals had at least one recorded diagnosis of hyperthyroidism. Of these, 5,839 cases were diagnosed after January 1, 1997, with seven individuals dying on the day of diagnosis, which were excluded from the analysis. After PSM and exclusion criteria to the control population, 5,832 cases were successfully matched with 22,579 controls, forming the analysis population for this study (Supplementary Fig. S1).

The baseline characteristics of the participants are presented in Table 1. Among patients with hyperthyroidism, 77.1% were female, with an average age at diagnosis of 60.1 years. The characteristics of the case and control groups were comparable, with an average follow-up time of 10.8 years (interquartile range (IQR) 4.0–17.7) and 11.5 years (IQR 4.8–18.1), respectively.

**Table 1:** Baseline characteristics of the study population.

Characteristic	Overall (*n* = 28,411)	Hyperthyroidism cases* (*n* = 5,832)	PSM matched controls^†^ (*n* = 22,579)
Age at index date, mean (SD), y	59.9 ± 11.1	60.1 ± 11.6	59.9 ± 11.0
Gender, no. (%)			
Female	21,862 (76.9)	4,495 (77.1)	17,367 (76.9)
Male	6,549 (23.1)	1,337 (22.9)	5,212 (23.1)
BMI, mean (SD), kg/m^2^	28.1 ± 5.44	28.1 ± 5.36	28.1 ± 5.46
Townsend deprivation index, mean (SD)	−1.01 ± 3.18	−1.01 ± 3.16	−1.01 ± 3.19
Educational attainment, no. (%)			
College or university degree	7,962 (28.0)	1,629 (27.9)	6,333 (28.0)
A level/AS level or equivalent	2,961 (10.4)	613 (10.5)	2,348 (10.4)
O level/GCSE or equivalent	6,088 (21.4)	1,249 (21.4)	4,839 (21.4)
Other	5,098 (17.9)	1,071 (18.4)	4,027 (17.8)
None of the above	6,302 (22.2)	1,270 (21.8)	5,032 (22.3)
Smoking status, no. (%)			
Current	3,557 (12.5)	727 (12.5)	2,830 (12.5)
Previous	9,927 (34.9)	2,060 (35.3)	7,867 (34.8)
Never	14,927 (52.5)	3,045 (52.2)	11,882 (52.6)
Alcohol consumption, no. (%)			
Three times a week or more	9,062 (31.9)	1,848 (31.7)	7,214 (32.0)
Once or twice a week or less	16,044 (56.5)	3,304 (56.7)	12,740 (56.4)
Never	3,305 (11.6)	680 (11.7)	2,625 (11.6)
Levels of physical activity, no. (%)			
Low	10,337 (36.4)	2,115 (36.3)	8,222 (36.4)
Moderate	9,462 (33.3)	1,957 (33.6)	7,505 (33.2)
High	8,612 (30.3)	1,760 (30.2)	6,852 (30.3)
Follow-up time, median (IQR), y	11.40 (4.61–18.00)	10.8 (4.03–17.70)	11.50 (4.80–18.10)

SD, standard deviation; IQR, interquartile range; PSM, propensity score matching; BMI, body mass index.

* In the UK Biobank, 5,839 individuals were diagnosed with hyperthyroidism after January 1, 1997. Seven individuals who died on the day of diagnosis were excluded from the analysis.

† PSM was conducted among individuals who had never been diagnosed with hyperthyroidism during follow-up, using a 1:4 matching ratio to match each case with four controls. After PSM, control individuals who died or were lost to follow-up before the index date, as well as those with records of hyperthyroidism treatment or a history of thyroid surgery were excluded from analysis.

### PheWAS

**Figure 2:**
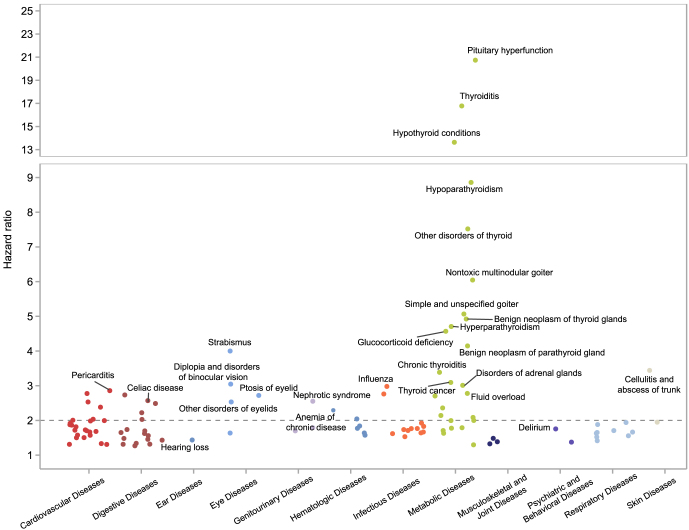
Results of the PheWAS analysis of hazard ratios for subsequent diseases among individuals with hyperthyroidism compared with matched controls. The x-axis represents disease categories by physiological system, and the y-axis represents the hazard ratios. Each point corresponds to a phenotype that was significantly associated with prior diagnosed hyperthyroidism after Bonferroni correction. Detailed results of PheWAS analysis are provided in Supplementary Table S2.

A total of 412 candidate phenotypes that co-occurred with hyperthyroidism in at least 20 cases were examined. After Bonferroni correction, 110 phenotypes were significantly associated with prior diagnosed hyperthyroidism (Fig. 2 and Supplementary Table S2). The top five complications with the highest risk were pituitary hyperfunction (hazard ratio (HR) = 20.45, 95% confidence interval (CI): 8.54–48.94), thyroiditis (HR = 18.99, 95% CI: 7.9–45.66), hypothyroidism conditions (HR = 14.79, 95% CI: 13.18–16.59), hypoparathyroidism (HR = 9.61, 95% CI: 5.72–16.13) and other thyroid disorders (HR = 8.64, 95% CI: 6.39–11.67). Phenotypes with the highest hazard ratios were predominantly related to the pituitary, thyroid and parathyroid glands. These conditions typically developed shortly after the diagnosis of hyperthyroidism; for example, the median time interval was 0.8 years (IQR 0.3–3.3) for benign parathyroid tumors, 1.5 years (IQR 0.6–3.4) for benign thyroid tumors and 1.8 years (IQR 1.1–5.4) for nontoxic uninodular goiter.

In addition, hyperthyroidism increases the risk of diseases across multiple systems, including cardiovascular diseases (e.g., atrial fibrillation, coronary atherosclerosis and heart failure), metabolic diseases (e.g., diabetes), musculoskeletal diseases (e.g., osteoporosis), digestive system disorders (e.g., esophagitis and GERD), eye diseases (e.g., lacrimal gland and eyelid diseases) and renal diseases (e.g., renal failure and nephrotic syndrome). The most common categories were endocrine disorders (26), cardiovascular diseases (24) and digestive system diseases (17).

The diagnosis of hyperthyroidism was significantly associated with an elevated risk of all-cause mortality (HR = 1.87, 95% CI: 1.71–2.04). Furthermore, increased risks were observed for four specific causes of mortality: malignant neoplasms death (MND) (HR = 1.49,95% CI: 1.30–1.70), cardiovascular disease death (CVDD) (HR = 1.82, 95% CI: 1.49–2.21), respiratory system disease death (RSDD) (HR = 1.76, 95% CI: 1.33–2.35) and digestive system disease death (HR = 2.03, 95% CI: 1.36–3.04).

### Landmark analysis

For the vast majority of phenotypes, effects of prior diagnosed hyperthyroidism showed a rapid gradient decline as the time since diagnosis increased, indicating a strong short-term impact of hyperthyroidism on health decline (Supplementary Tables S4 and S5). For example, the HRs of hyperthyroidism for atrial fibrillation were 2.36 (95% CI: 1.74–3.20) within 2 years, 1.53 (95% CI: 1.14–2.06) for 2–5 years, 1.21 (95% CI: 0.93–1.58) for 5–10 years and 1.09 (95% CI: 0.92–1.31) beyond 10 years since index date. The risk increased most significantly within 2 years, including cardiovascular complications such as chronic ischemic heart disease (HR = 3.64, 95% CI: 2.61–5.08), heart failure (HR = 3.44, 95% CI: 2.47–4.79) and cerebral ischemia (HR = 4.12, 95% CI: 2.63–6.47). Hypothyroidism (HR = 21.79, 95% CI: 17.00–27.93) and other thyroid disorders (HR = 20.65, 95% CI: 10.15–42.04) were typically diagnosed within 2 years after the diagnosis of hyperthyroidism. The risk increase for all-cause mortality due to hyperthyroidism was most marked within 2 years (HR = 5.62, 95% CI: 4.65–6.80), while there was no significant risk increase after 10 years (HR = 1.01, 95% CI: 0.87–1.18).

### Disease trajectory analysis

The process of disease pair selection is illustrated in Supplementary Fig. S5, with detailed information on the frequency and association strength of each pair provided in Supplementary Table S6. Results of the mediation analysis are presented in Supplementary Table S7, with 121 D1 → D2 → D3 demonstrating a statistically significant indirect effect of D2, suggesting that multiple disease pairs can be connected through D2.

**Figure 3:**
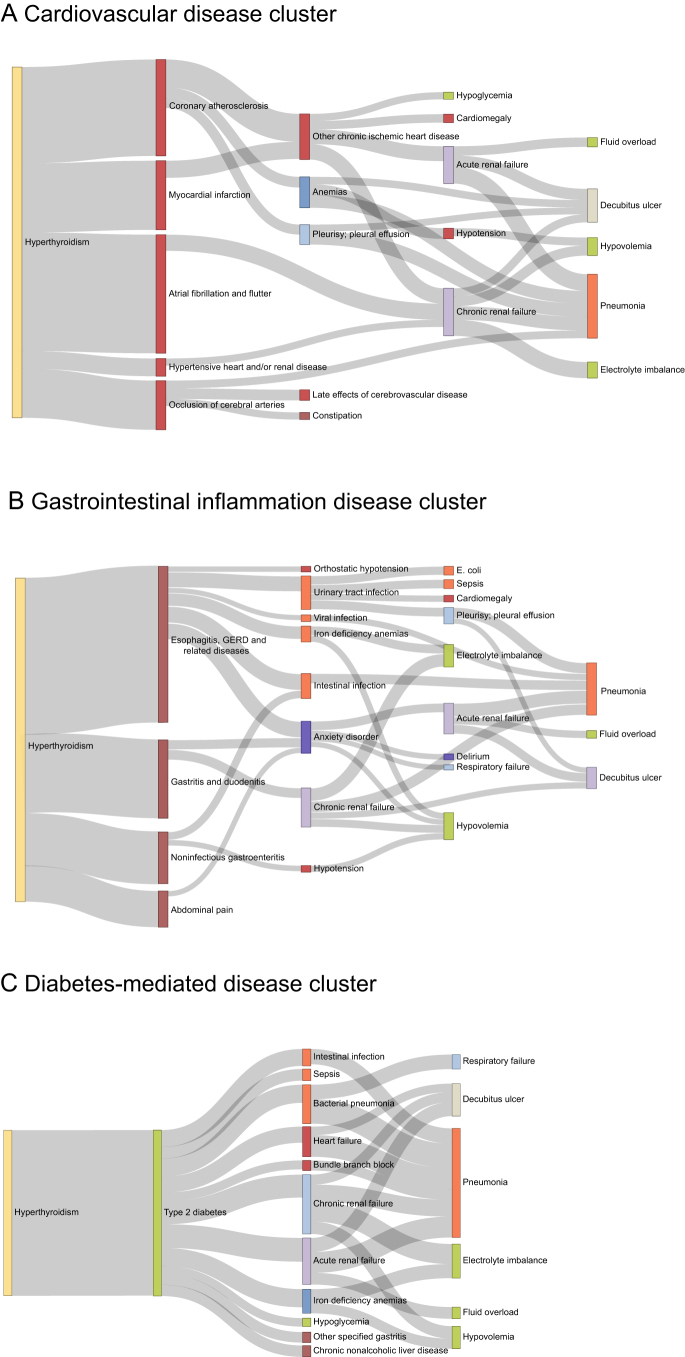
Disease trajectories of three main disease clusters affected by hyperthyroidism: (A) cardiovascular disease cluster, (B) gastrointestinal inflammation disease cluster and (C) diabetes-mediated disease cluster. In the Sankey diagram, each node represents a phenotype. The color of the node indicates disease categories by physiological system. Links between nodes represent associations between disease pairs, with the width of the links reflecting the frequency of these disease pairs among participants with hyperthyroidism. Detailed information on disease pairs is listed in Supplementary Table S6.

An overview of disease trajectory following hyperthyroidism is depicted in Supplementary Fig. S6. Three primary disease clusters were identified based on similarities in affected systems according to phenotypes directly associated with hyperthyroidism (Fig. 3). Cluster 1 originated from cardiovascular diseases, where hyperthyroidism directly contributed to a cascade of increased risk for adverse cardiovascular events, such as coronary artery atherosclerosis, myocardial infarction, atrial fibrillation, cerebral artery occlusion and hypertensive cardiac disease. These conditions eventually led to complications such as pneumonia and acute kidney failure. Cluster 2 started with gastrointestinal (GI) inflammation (esophagitis and gastroenteritis), which subsequently coexisted with psychiatric disorders such as anxiety and more severe conditions such as delirium. Cluster 3 was diabetes-mediated diseases, where hyperthyroidism exerts a direct elevated risk of diabetes. Subsequent complications, such as increased susceptibility to metabolic disturbances, infectious diseases and renal failure, were partly mediated by the onset of diabetes.

**Figure 4:**
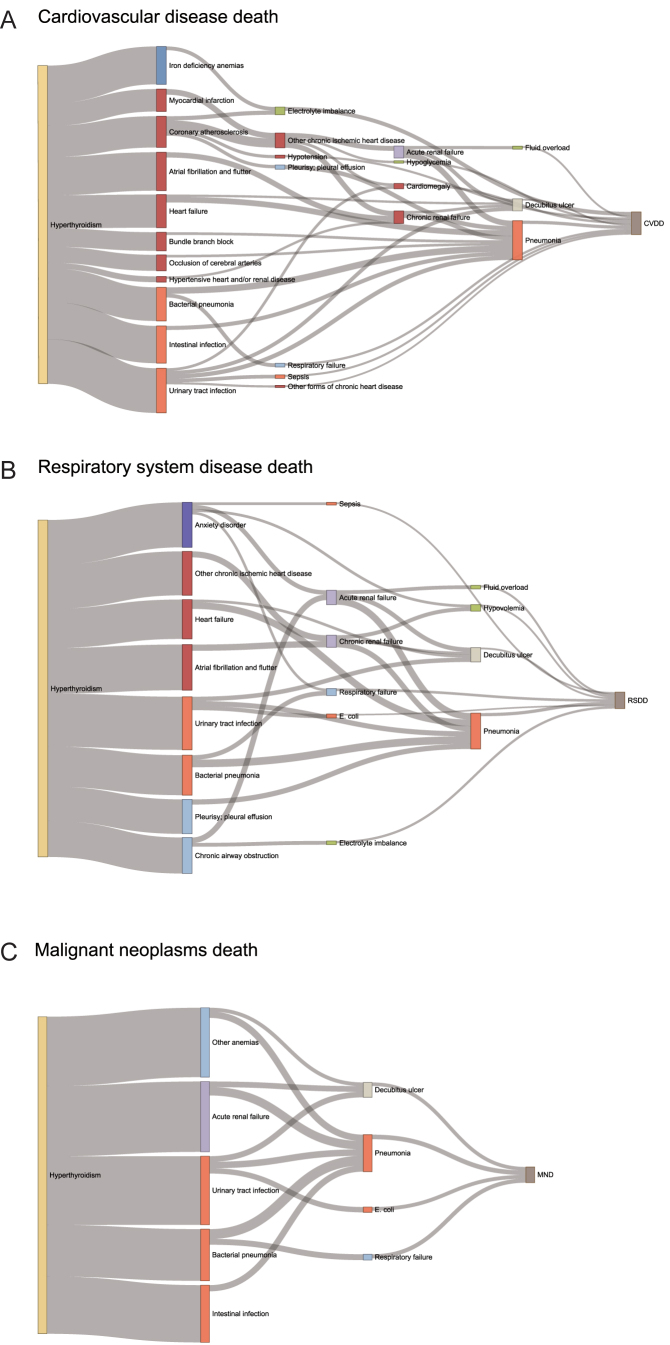
Disease trajectories following hyperthyroidism leading to three main causes of death: (A) CVDD, (B) RSDD and (C) MND. In the Sankey diagram, each node represents a phenotype. The color of the node indicates disease categories by physiological system. Links between nodes represent associations between disease pairs, with the width of the links reflecting the frequency of these disease pairs among participants with hyperthyroidism. Detailed information on disease pairs is listed in Supplementary Table S8. CVDD, cardiovascular disease death; RSDD, respiratory system disease death; MND, malignant neoplasms death.

In the analysis of trajectories leading to mortality, three specific causes of death – CVDD, RSDD and MND – were considered, and trajectories were constructed for each cause (Fig. 4). The selection process for disease pairs is shown in Supplementary Fig. S7. Detailed information on the disease pairs and the results of the mediation analysis are presented in Supplementary Tables S8 and S9.

Trajectory leading to CVDD primarily consisted of cardiovascular diseases, including atrial fibrillation, ischemic cardiovascular and cerebrovascular diseases, cardiac conduction block and heart failure. Infectious diseases could exacerbate the cardiac burden and accelerate the deterioration of cardiac function, ultimately progressing to decompensated states such as pneumonia, fluid overload and hypotension, which directly contributed to CVDD. In the trajectory leading to RSDD, chronic obstructive pulmonary diseases (COPDs), infectious diseases and cardiac dysfunction all contributed to RSDD. Hyperthyroidism increased the risk of chronic airway obstruction, leading to a progressive decline in lung function, while heart failure contributed to fluid overload, ultimately resulting in pulmonary infections. In the trajectory leading to MND, an increased risk of infectious diseases (including GI, urinary tract and pulmonary infections), along with renal failure and anemia, can accelerate death in patients with malignant neoplasms.

### Subgroup and sensitive analysis

In the sex-stratified analysis (Supplementary Table S10), the results were largely consistent between male (*n* = 21,862) and female (*n* = 6,549). However, compared with females, males diagnosed with hyperthyroidism showed a slightly higher risk of developing most types of subsequent diseases, with a particularly significant increase in the risk of CVDD (HR = 2.38, 95% CI: 1.80–3.14 in males vs HR = 1.47, 95% CI: 1.11–1.94 in females).

For most conditions, particularly cardiovascular diseases, the hazard ratios increased with the age at diagnosis. However, for ocular diseases, such as eyelid disorders and lacrimal system diseases, the hazard ratios decreased with the age at diagnosis (Supplementary Fig. S8).

The results of the sensitivity analysis are listed in Supplementary Tables S11 and S12. After excluding diagnostic records within 6 months of the index date, hazard ratios for most phenotypes were slightly declined. In the subgroup with at least two recorded diagnoses of hyperthyroidism, spaced more than 3 months apart (*n* = 4,724), the risk of certain conditions, such as atrial fibrillation, osteoporosis and type 2 diabetes, was increased. After excluding hyperthyroidism cases with prior diagnoses of other thyroid disorders before the index date (*n* = 19,299), the results remained largely consistent with the main analysis. In the subgroup with no history of malignancy diagnosis before the index date (*n* = 21,540), the results were substantially consistent with the main analysis but showed no significant association between prior diagnosed hyperthyroidism and MND.

## Discussion

Based on the UK Biobank, this prospective study investigated the impact of prior diagnosed hyperthyroidism on subsequent diseases and visualized the progression of the diseases through disease trajectories. Our findings indicated that the onset of hyperthyroidism increased the risk of 110 phenotypes, all-cause mortality and four specific causes of death, with the impact on adverse health outcomes being most pronounced within the first 2 years after diagnosis. It was found that the hyperthyroidism promoted the development of three disease clusters: cardiovascular disease cluster, GI inflammatory disease and diabetes-mediated disease cluster. Trajectories leading to death were also investigated, revealing the pathways through which hyperthyroidism contributed to mortality. These findings systematically uncovered the downstream conditions of hyperthyroidism, offering new clinical insights to improve the prognosis of patients with hyperthyroidism.

Conditions with the highest hazard ratios associated with prior diagnosed hyperthyroidism were either etiologies of hyperthyroidism or complications resulting from the treatment of hyperthyroidism: i) long-term follow-up studies have shown that hypothyroidism was an inevitable consequence of radioiodine therapy in patients with hyperthyroidism of multiple etiologies (28, 29). Patients with Graves’ disease may naturally evolve to hypothyroidism over time due to the transition of TSH receptor antibody types from initial stimulating antibodies to blocking antibodies, or the gradual depletion of thyroid function due to chronic inflammatory processes (30); ii) in the early stages of Hashimoto’s thyroiditis, transient hyperthyroidism can occur as the immune system attacks thyroid cells, leading to a substantial release of THs into the bloodstream. As TH stores are depleted, patients gradually transition to hypothyroidism over a period of 3–24 months (31); iii) hyperthyroidism was typically accompanied by the suppression of pituitary function. However, the analysis suggested that there was an increased risk of pituitary hyperfunction after hyperthyroidism. One possible explanation was that the compensatory elevation of TSH in patients who later develop hypothyroidism may have been misdiagnosed as pituitary hyperfunction. Another possibility was a delayed diagnosis of central hyperthyroidism (32); iv) the strong association between hyperthyroidism and nontoxic goiter or benign neoplasm of the thyroid gland can be explained by detection bias, where patients with hyperthyroidism who undergo thyroid ultrasound are also likely to be identified with other occult thyroid conditions; v) hyperparathyroidism can occur due to radioactive iodine therapy (33), while hypoparathyroidism may result from thyroidectomy (34, 35).

Cardiovascular system is now an established system significantly affected by hyperthyroidism, and the mechanisms linking hyperthyroidism and cardiovascular disease have been well-understood (6, 7, 36, 37). Hyperthyroidism leads to hyperdynamic circulation, increased supraventricular ectopic activity and enhanced platelet plug formation (38). Previous studies, consistent with the findings of this study, indicated that hyperthyroidism increases the risk of multiple cardiovascular diseases, including atrial fibrillation (39), heart failure (17), coronary artery disease (40), stroke (40), heart valve involvement (41) and cardiovascular mortality (42). Polygenic PheWAS also revealed that genetic associations represented by polygenic scores of elevated FT3 levels and high TSH levels were associated with an increased risk of multiple cardiovascular diseases (43). Disease trajectories indicated that progressive cardiovascular diseases can further impair renal function, leading to disturbances in fluid and electrolyte metabolism. Management of cardiovascular complications including the early use of beta-blockers has been a crucial part of the treatment guidelines for hyperthyroidism (44).

Hyperthyroidism is associated with GI dysmotility (45), disruption of gut microbial homeostasis (46) and Helicobacter pylori infection (47), which can lead to GI inflammatory diseases. In addition, there is an increased risk of autoimmune GI diseases such as autoimmune gastritis and inflammatory bowel disease in patients with Graves’ disease (48, 49). Anxiety and GI disorders are frequently comorbid (50, 51); disease trajectories in our analyses also showed a strong correlation between anxiety and multiple GI disorders, with anxiety disorders commonly developing subsequent to GI inflammation, possibly due to the more pronounced symptoms of GI inflammation. Our research suggested that the adverse effects of hyperthyroidism on GI inflammation may be underestimated, which indicated an increased risk of further complications including renal failure, fluid imbalance, anemia and acute infections.

It has been reported that there is a co-existence of hyperthyroidism and diabetes, with an increased risk of diabetes in patients with hyperthyroidism (52). Studies have suggested that excess THs raise blood glucose levels through several pathways, including stimulating glucagon secretion, increasing gluconeogenesis and glycogenolysis and inducing insulin resistance in the liver and peripheral tissues (53, 54). Our study revealed that the diabetes secondary to hyperthyroidism was a key factor in the health decline of patients with hyperthyroidism, which partly mediated conditions such as renal failure, iron deficiency anemia, acute infections and cardiac dysfunction.

A meta-analysis revealed that mortality increased by 20% in patients with hyperthyroidism (55). On one hand, hyperthyroidism increases the risk of several life-threatening conditions, such as stroke and myocardial infarction; on the other hand, severe hyperthyroidism, such as a thyrotoxic crisis (56), can directly result in death. The link between hyperthyroidism and RSDD has rarely been reported. Our study found that hyperthyroidism raised the risk of COPD, which contributes to RSDD. Previous studies have shown that hypothyroidism can exacerbate gas exchange function in patients with COPD (57). The link between hyperthyroidism and COPD, and its possible mediation by hypothyroidism secondary to hyperthyroidism, requires further investigation. Although previous studies have reported that hyperthyroidism increased the risk of other cancers (58, 59, 60), no association was found between hyperthyroidism and cancers other than thyroid cancer in our study, which can be attributed to the stringent multiple corrections applied. After excluding individuals with a history of malignancy before the index date, the association between hyperthyroidism and malignancy mortality was no longer significant, indicating that the contribution of hyperthyroidism to MND was primarily due to the accelerated decline in the health of patients with malignancy.

In the landmark analysis, we found that the risk of subsequent diseases increased most significantly in the short term, especially within the first 2 years. One possible reason for this is that the effects of hyperthyroidism can diminish over time as a result of effective treatment. For example, 12–18 months of treatment with antithyroid drugs can lead to remission in about 50% of patients (61). Second, animal and cell experiments have demonstrated that excessive THs lead to downregulation of thyroid hormone receptor expression, which reduces the overstimulation of tissues by THs. This represented a compensatory mechanism in response to long-term TH excess (62, 63). One hypothesis is that the stress response induced by hyperthyroidism is most pronounced in the early stages, when the body has not yet established sufficient compensatory mechanisms, making it more susceptible to complications in the short term. This physiological stress response can be partially adapted over time. Furthermore, the treatment of hyperthyroidism imposes an additional burden on the patient’s physiological state, while the psychological burden and mental stress following the diagnosis of hyperthyroidism, together with follow-up visits that may involve more intensive medical examinations and monitoring, can also explain part of the strong short-term effects. These findings suggest that greater attention should be paid to the prevention of complications in the short term after the diagnosis of hyperthyroidism.

Subgroup analysis stratified by gender indicated that men had slightly higher hazard ratios for most diseases compared with women, which can be explained by the fact that men are more likely to experience treatment failure and have a higher recurrence rate (64, 65). Previous studies found that elderly patients were more susceptible to the negative effects of excessive TH (66), with cardiovascular system being primarily affected and less pronounced eye symptoms (67, 68), which was consistent with the results of our analysis.

The main strengths of this study are as follows. Based on a large community-based cohort, with a median follow-up time of over 10 years and complete diagnostic records, this study systematically analyzed the impact of hyperthyroidism on the risk across a broad range of diseases under a hypothesis-free framework. Disease trajectory analysis was conducted to understand the complex interactions between hyperthyroidism-related conditions, providing a longitudinal perspective on disease progression.

Our study has several limitations. First, the arrows in the disease trajectories did not indicate causal relationships between diseases, as the study focused on identifying sequential patterns between diagnoses, which may be influenced by clinical factors. Second, only the first diagnosis of each phenotype was considered, limiting the investigation of the impact of hyperthyroidism on disease recurrence. Third, the UK Biobank lacks detailed information on the causes of hyperthyroidism. While most cases are attributed to Graves’ disease, other potential causes could lead to different impacts on subsequent disease development. Fourth, this study did not account for the effects of different hyperthyroidism treatment methods and the severity and duration of hyperthyroidism. Further research is required to address these aspects.

## Conclusion

This study comprehensively demonstrated that hyperthyroidism increased the risk of 110 types of comorbidities distributed in multiple systems and all-cause mortality, and mortality from four specific causes, with particularly marked short-term adverse effects within the first 2 years of diagnosis. Disease trajectory analysis revealed that cardiovascular disease cluster, gastrointestinal inflammatory disease cluster and diabetes-mediated disease cluster were strongly associated with the subsequent health decline in patients with hyperthyroidism. Findings of this study provided insights for better management of the complications in hyperthyroid patients.

## Supplementary materials



## Declaration of interest

The authors declare that there is no conflict of interest that could be perceived as prejudicing the impartiality of the work reported.

## Funding

This study was funded by the National Natural Science Foundation of Chinahttps://doi.org/10.13039/501100001809 (82473728 to YW, 82220108002 to FC and 82273737 to RZ).

## Author contribution statement

Yongyue Wei, Qiuyuan Chen and Doudou Chen conceived and designed the study. Qiuyuan Chen, Yunhao Zheng and Zaiming Li performed the statistical analysis. Qiuyuan Chen, Doudou Chen and Xinpan Wang drafted the manuscript. Longyao Zhang prepared the figures. Xiaoyu Wu and Qin Chen reviewed the literature. Yongyue Wei, Xuqin Zheng, Feng Chen, Tao Yang and Ruyang Zhang critically reviewed the interpretation of the results and revised the manuscript. All authors read and approved the final version of the manuscript.

## Data availability

This study was conducted using the UK Biobank under application number 124348. The authors affirm that the manuscript is an honest, accurate and transparent account of the study being reported; that no important aspects of the study have been omitted; and that any discrepancies from the study as planned have been explained. Researchers can access the data by submitting an application to the UK Biobank. For detailed guidelines on the application process, please visit http://www.ukbiobank.ac.uk/.

## Ethical approval

UK Biobank was approved by the NHS National Research Ethics Service (reference 16/NW/0274). Written informed consent was obtained from all participants. This study did not require participants to be re-contacted, and no separate ethics approval was required according to the UK Biobank Ethics and Governance Framework (EGF).
