# Supplementary material for: Increased risk of multisystem comorbidities and disease trajectories following hyperthyroidism: evidence from the 0.5 million UK Biobank population
Source: Endocr Connect. 2025 Apr 23;14(5):e250066. doi: 10.1530/EC-25-0066 (PMC12020380; doi:10.1530/EC-25-0066)
Supplement: Supplementary file 1 [file supplementary_materials.pdf]

Increased risk of multisystem comorbidities and disease trajectories following hyperthyroidism: evidence from 0.5 million UK Biobank population

Supplementary materials

Outline

Methods ... 3

Section 1: Details of covariate preprocessing and PSM ... 3
Section 2: Landmark analysis ... 4
Section 3: Construction of disease trajectory ... 5
Section 4: Construction of trajectory leading to death ... 5
Section 5: Details about restrictions in sensitivity analysis ... 6

PheWAS ... 9

Table S1. Rules for categorizing underlying causes of death into 16 major causes of death. ... 9
Figure S1. Flowchart of study participants in PheWAS. ... 11
Figure S2. Results of the PheWAS analysis of hazard ratios for subsequent diseases among individuals with hyperthyroidism compared with matched controls. ... 12
Table S2. Results of the PheWAS analysis of hazard ratios with 95% confidence intervals for subsequent diseases. ... 13
Table S3. Associations between prior diagnosed hyperthyroidism and causes of death. ... 17
Figure S3. Cumulative hazard of multisystem diseases after prior diagnosed hyperthyroidism. ... 18
Figure S4. Cumulative hazard of mortality after prior diagnosed hyperthyroidism. ... 20

Landmark Analysis ... 21

Table S4. Results of landmark analyses to investigate the time-varying effect of prior diagnosed hyperthyroidism on subsequent diseases. ... 21
Table S5. Results of landmark analyses to investigate the time-varying effect of prior diagnosed hyperthyroidism on causes of death. ... 25

Disease Trajectory Analysis ... 26

Figure S5. Research flowchart for identifying disease pairs in the disease trajectory following hyperthyroidism. ... 26
Table S6. Prevalence and odds ratios with 95% confidence intervals for the disease pairs in disease trajectories following hyperthyroidism. ... 27
Table S7. Mediation effect of D2 within a length-3 disease trajectory D1→D2→D3 in disease trajectories following hyperthyroidism. ... 31
Figure S6. An overall view of disease trajectory following hyperthyroidism. ... 35
Figure S7. Research flowchart for identifying disease pairs in the disease trajectory leading to death among participants diagnosed with hyperthyroidism. ... 36
Table S8. Prevalence and odds ratios with 95% confidence intervals for the disease pairs in disease trajectories leading to death. ... 37
Table S9. Mediation effect of D2 within a length-3 disease trajectory D1→D2→D3 in disease trajectories leading to death. ... 42

Subgroup and Sensitivity Analysis ... 46

Table S10. Associations between prior diagnosed hyperthyroidism and subsequent diseases stratified by gender. ... 46
Figure S8. Moderation effect of age at diagnosis characterized by restricted cubic spline. ... 50
Table S11. Sensitivity analyses to investigate the association between prior diagnosed hyperthyroidism and subsequent diseases with different restrictions. ... 52
Table S12. Sensitivity analyses to investigate the association between prior diagnosed hyperthyroidism and death causes with different restrictions. ... 56

Supplementary References ... 57

Methods

Section 1: Details of covariate preprocessing and PSM

The proportion of missing values for each variable was presented as follows. Missing data were imputed using the median, stratified by sex and age.

Physical activity levels were calculated using total metabolic equivalent (MET) hours of physical activity per week, with the duration of each activity level weighted by the estimated MET value (3.3, 4.0 and 8.0 METs for walking, moderate and vigorous intensity, respectively). Following the IPAQ guidelines, physical activity was recoded as 0 for any category of less than 10 minutes per day, and durations of >180 minutes per day were truncated. The level of physical activity for each participant is categorized as low, moderate or vigorous according to the three digits of the MET value. Townsend deprivation index was assigned to each individual based on their postcode location, with a higher score indicating a higher level of deprivation.

PSM was conducted with a 1:4 matching ratio using the nearest neighbor method and a caliper width of 0.1 to ensure closely matched pairs. Propensity scores were derived from a generalized linear model, incorporating covariates such as year of birth, gender, baseline BMI, Townsend Deprivation Index, educational level, smoking status, alcohol consumption, and physical activity levels.

Table. Detailed information on variables in PSM.

Covariate Name		No. Missing (%)	Possible Values	Source
Year of birth		0	continuous variable	Entered by participant.
Gender		0	Female, male	Acquired from central registry at recruitment, but in some cases updated by the participant.
Body mass index (BMI)		3107 (0.62)	continuous variable	Constructed from height and weight measured during the initial Assessment center visit.
Educational attainment		3495 (0.70)	College or University degree, A levels/AS levels or equivalent, O levels/GCSEs or equivalent,other, none of the above	ACE touchscreen question "Which of the following qualifications do you have? (You can select more than one)"
Assessment center		0	22 assessment centers	Automatically acquired at participant consent
Townsend deprivation index		14835 (2.95)	continuous variable	Based on the preceding national census output areas. Each participant is assigned a score corresponding to the output area in which their postcode is located.
Smoking status		2950 (0.59)	Never, previous, current	ACE touchscreen question "Do you smoke tobacco now?" "In the time that you smoked, did you ever stop for more than 6 months?"
Alcohol consumption		1502 (0.30)		ACE touchscreen question "About how often do you drink alcohol?"
Levels of physical activity	Number of days/week walked 10+ minutes	10466 (2.08)	Low, median, high	ACE touchscreen question "In a typical WEEK, on how many days did you walk for at least 10 minutes at a time? (Include walking that you do at work, travelling to and from work, and for sport or leisure)"
	Number of days/week of moderate physical activity 10+ minutes	27262 (5.43)		ACE touchscreen question "In a typical WEEK, on how many days did you do 10 minutes or more of moderate physical activities like carrying light loads, cycling at normal pace? (Do not include walking)"
	Number of days/week of vigorous physical activity 10+ minutes	27573 (5.49)		ACE touchscreen question "In a typical WEEK, how many days did you do 10 minutes or more of vigorous physical activity? (These are activities that make you sweat or breathe hard such as fast cycling, aerobics, heavy lifting)"
	Duration of walks	76894 (15.31)		ACE touchscreen question "How much time do you usually spend walking on one of those days? "
	Duration of moderate activity	134037 (26.68)		ACE touchscreen question "How many minutes did you usually spend doing moderate activities on a typical DAY?"
	Duration of vigorous activity	232940 (46.37)		ACE touchscreen question "How many minutes did you usually spend doing vigorous activities on a typical DAY?"

Section 2: Landmark analysis

Effect of time-varying risk	Risk factor	Population	Follow-up time
HR_2	Prior diagnosed hyperthyroidism at index date	Exclusion: participants diagnosed with the analyzed phenotype before index date	2 year
HR_{2-5}		Exclusion: (1) Participants diagnosed with the analyzed phenotype before index date; (2) Participants diagnosed with the analyzed phenotype within 2 years after the index date; (3) Participants died or were lost to follow-up within 2 years after the index date.	3 year
HR_{5-10}		Exclusion: (1) Participants diagnosed with the analyzed phenotype before index date; (2) Participants diagnosed with the analyzed phenotype within 5 years after the index date; (3) Participants died or were lost to follow-up within 5 years after the index date.	5 year
HR_{10}		Exclusion: (1) Participants diagnosed with the analyzed phenotype before index date; (2) Participants diagnosed with the analyzed phenotype within 10 years after the index date; (3) Participants died or were lost to follow-up within 10 years after the index date.	Until end of follow-up

Figure. Schematic diagram of Landmark analysis.

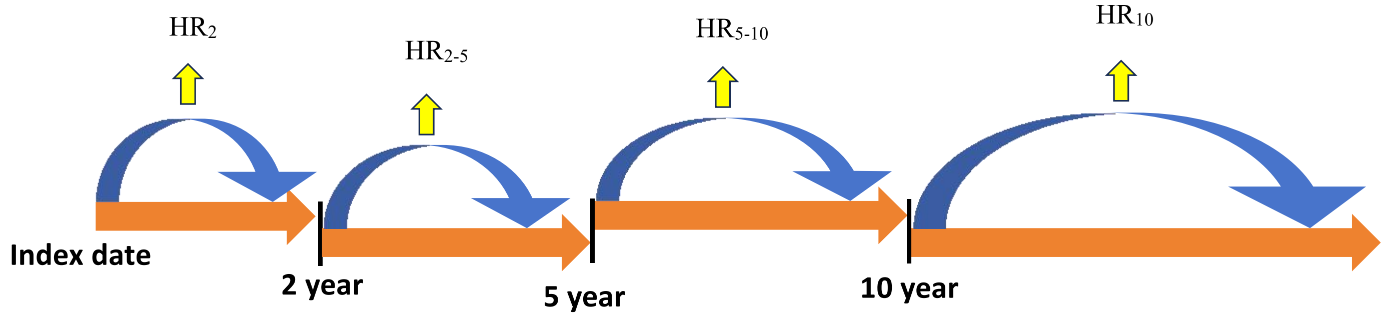

Section 3: Construction of disease trajectory

Step 1: Phenotype selection.

Stratified Cox regression was used to identify associations between prior diagnosed hyperthyroidism and subsequent diseases. Only phenotypes that co-occurred with hyperthyroidism in at least 20 cases were included in our analysis. Each subcohort consisted of one case and multiple controls. The index date was established as the date of entry into the cohort. Follow-up concluded with the earliest of the following events: onset of the specific disease, death, loss to follow-up, or the censoring date. Individuals who had previously been diagnosed with the analyzed phenotype prior to the index date were excluded. Phenotypes with significant associations after Bonferroni correction and HR > 1 were selected for further analysis.

Step 2: Identify all possible D1→D2 pairs.

For each patient diagnosed with hyperthyroidism, diagnostic records after the index date were extracted and all D1→D2 pairs were identified (i.e., the patient is first diagnosed with D1, then diagnosed with D2, which can be considered to have D1→D2). Frequency of each D1→D2 disease pair across all hyperthyroidism patients were calculated, and disease pairs D1→D2 with at least 10 cases were selected for further analysis.

Step 3: Temporal order of D1→D2 pairs.

To ensure temporal order between D1 and D2, binomial test was used to evaluate the statistical significance of the sequential relationship of D1→D2. The disease pair was selected for further analysis only if the proportion of D1 preceding D2 significantly exceeded 50% among individuals diagnosed with both D1 and D2. Patients with the same diagnosis date for D1 and D2 were not included in the analysis. D1→D2 pairs with binomial test p-value < 0.05 after Bonferroni correction were selected for further analysis.

Step 4: Association strength of D1→D2 pairs.

For each analysis, a case-control dataset was constructed, with D1 as the exposure and D2 as the outcome. Logistic regression was used to assess the magnitude of the associations between each disease pair, adjusted for year of birth, gender, baseline BMI, Townsend Deprivation Index, educational level, smoking status, alcohol consumption, and physical activity levels. Participants diagnosed with D1 or D2 before the index date, or with D2 diagnosed prior to D1, were excluded from each analysis. D1→D2 pairs with significant associations after Bonferroni correction and OR > 1 were selected.

Step 5: Combine D1→D2 pairs for longer disease trajectory.

Multiple D1→D2 pairs selected in step 4 were combined to form longer disease trajectories. If the three disease pairs D1→D2, D2→D3, and D1→D3 coexisted, mediation analyses with 1000 bootstrap simulations were conducted with D2 serving as the mediator. The sequence D1→D2→D3 was then linked into a disease trajectory of length 3 if a statistically significant indirect effect of D2 was observed in the D1→D2→D3 pathway. Similarly, disease trajectories of longer length can be constructed by mediating the linkage of diseases, culminating in a comprehensive network of disease trajectories.

Section 4: Construction of trajectory leading to death

Step 1: Phenotype selection.

For phenotypes: Stratified Cox regression was used to identify associations between prior diagnosed hyperthyroidism and subsequent diseases. Only phenotypes that co-occurred with hyperthyroidism in at least 20 cases were included in our analysis. Each subcohort consisted of one case and multiple controls. The index date was established as the date of entry into the cohort. Follow-up concluded with the earliest of the following events: onset of the specific disease, death, loss to follow-up, or the censoring date. Individuals who had previously been diagnosed with the analyzed phenotype prior to the index date were excluded. Phenotypes with significant associations after Bonferroni correction and HR > 1 were selected for further analysis.

For death causes: The underlying cause of death coded according to ICD-10 was mapped to PheCodes, and categorized into 16 major causes of death. Fine-Gray competing risk model was used to analyze the impact of hyperthyroidism on each causes of death, adjusted for year of birth, gender, baseline BMI, Townsend Deprivation Index, educational level, smoking status, alcohol consumption, and physical activity levels. Only causes of death that co-occurred with hyperthyroidism in at least 20 cases were included in our analysis. Death causes with significant associations after Bonferroni correction and HR > 1 were selected for further analysis.

Step 2: D→Death pair selection.

Among phenotypes and death causes selected in step 1, all D→Death pairs were identified. Only D→Death pairs with at least 10 cases were selected for further analysis. For each D→death pair, a case-control dataset was constructed, with D as the exposure and specific death cause as the outcome. Logistic regressions was used to assess the associations between each D→Death pair, adjusted for year of birth, gender, baseline BMI, Townsend Deprivation Index, educational level, smoking status, alcohol consumption, and physical activity levels. Participants diagnosed with D before the index date were excluded from each analysis. For each death cause, phenotypes with significant associations after Bonferroni correction and OR > 1 were selected.

Step 3: D1→D2 pair selection.

For each death cause, Steps 3-5 in Section 3 were repeated to construct trajectories leading to death.

Section 5: Details about restrictions in sensitivity analysis

Any of the following diagnosed phenotypes before the index date is defined as having other thyroid disorders:

PheCode	Phenotype
244	Hypothyroid conditions
240	Simple and unspecified goiter
241.2	Nontoxic multinodular goiter
246	Other disorders of thyroid
241.1	Nontoxic uninodular goiter
241	Nontoxic nodular goiter
242	Thyrotoxicosis with or without goiter
242.1	Graves' disease
242.2	Toxic multinodular goiter
245	Thyroiditis
245.1	Thyroiditis, acute and subacute
245.2	Chronic thyroiditis
242.3	Exophthalmos
246.7	Abnormal results of function study of thyroid

Any of the following diagnosed phenotypes before the index date is defined as having malignant tumors:

PheCode	Phenotype
145.1	Cancer of lip
145.2	Cancer of tongue
145.4	Cancer of the gums
145.5	Cancer of the mouth floor
145	Cancer of mouth
149	Cancer of larynx, pharynx, nasal cavities
195.1	Malignant neoplasm, other
145.3	Cancer of major salivary glands
149.1	Cancer of oropharynx
149.2	Cancer of nasopharynx
149.3	Cancer of hypopharynx
150	Cancer of esophagus
151	Cancer of stomach
159.2	Malignant neoplasm of small intestine, including duodenum
153.2	Colon cancer
153.3	Malignant neoplasm of rectum, rectosigmoid junction, and anus
153	Colorectal cancer
155	Cancer of liver and intrahepatic bile duct
155.1	Malignant neoplasm of liver, primary

159.3	Malignant neoplasm of gallbladder and extrahepatic bile ducts
157	Pancreatic cancer
159	Malignant neoplasm of other and ill-defined sites within the digestive organs and peritoneum
149.9	Cancer of of nasal cavities
149.4	Cancer of larynx
165.1	Cancer of bronchus; lung
164	Cancer of intrathoracic organs
165	Cancer within the respiratory system
170.1	Bone cancer
172.11	Melanomas of skin
172.2	Other non-epithelial cancer of skin
159.4	Malignant neoplasm of retroperitoneum and peritoneum
230	Kaposi's sarcoma
170.2	Cancer of connective tissue
174.1	Breast cancer [female]
184.2	Cancer of other female genital organs, excluding uterus and ovary
184.1	Malignant neoplasm of ovary and other uterine adnexa
180.1	Cervical cancer
182	Malignant neoplasm of uterus
187.1	Malignant neoplasm of unspecified male genital organ
185	Cancer of prostate
187.2	Malignant neoplasm of testis
189.11	Malignant neoplasm of kidney, except pelvis
189.12	Malignant neoplasm of renal pelvis
189.4	Malignant neoplasm of other urinary organs
189.2	Cancer of bladder
190	Cancer of eye
191.1	Cancer of brain and nervous system
193	Thyroid cancer
194	Cancer of other endocrine glands
195.3	Malignant neoplasm of head, face, and neck
198.1	Secondary malignancy of lymph nodes
198	Secondary malignant neoplasm
198.2	Secondary malignancy of respiratory organs
198.3	Secondary malignant neoplasm of digestive systems
198.4	Secondary malignant neoplasm of liver
198.7	Secondary malignant neoplasm of skin
198.5	Secondary malignancy of brain/spine
198.6	Secondary malignancy of bone

201	Hodgkin's disease
202.2	Non-Hodgkins lymphoma
204.4	Multiple myeloma
270.35	Macroglobulinemia
200	Myeloproliferative disease
204	Leukemia
204.1	Lymphoid leukemia
204.2	Myeloid leukemia
204.3	Monocytic leukemia
202	Cancer of other lymphoid, histiocytic tissue
277	Other disorders of metabolism

PheWAS

Table S1. Rules for categorizing underlying causes of death into 16 major causes of death.

death cause	PheCode
Benign neoplasms death	172.3, 180.3, 187, 195, 189, 210, 208, 211, 229, 212, 213, 214, 214.1, 228, 199, 215, 216, 218.1, 218.2, 220, 221, 222, 223, 224, 224.1, 225.1, 225.2, 225, 226, 227.1, 227.2, 227.3, 227, 229.1, 158, 184, 187.8, 191, 200.1, 270.32, 173, 174.3, 610.4
Blood system death	280.1, 280.2, 281.1, 281, 281.9, 282.9, 282.8, 282.5, 289, 283, 283.1, 283.2, 285.8, 284, 284.2, 285.1, 285.2, 285, 285.3, 286.6, 286.1, 286.7, 286.5, 286.4, 287, 287.2, 287.4, 287.3, 288.11, 288, 288.2, 288.3, 289.5, 289.9, 289.8, 279.1, 277.2, 279.8, 697, 270.31, 279
Cardiovascular disease death	394.4, 420.2, 420.1, 394.2, 394, 394.3, 394.7, 395.1, 395.2, 401.1, 401.2, 401.3, 411.3, 411.1, 411.2, 414, 411.9, 411.4, 411.8, 440, 415.11, 415, 415.2, 747.1, 395.3, 395.4, 395, 425.1, 425.8, 425, 425.2, 426.2, 426.3, 426.4, 426.8, 426, 427.42, 427.1, 427.2, 427.41, 427.6, 427.8, 427.3, 427.5, 428, 416, 430.1, 430.2, 430.3, 430, 433.2, 433.1, 442.4, 433.5, 433.8, 440.9, 440.1, 440.2, 442.1, 442, 442.8, 442.2, 442.3, 443.8, 443.1, 446.1, 443.9, 444, 444.2, 444.1, 447, 447.1, 446.9, 448, 217.1, 451, 451.2, 452, 454.1, 530.2, 454, 452.8, 459, 456, 459.9, 289.4, 450, 458.9, 458.1, 458.2, 429.1
Digestive system disease death	520, 520.1, 520.2, 521.1, 521.2, 521.4, 523, 521, 522, 522.1, 523.3, 522.5, 523.1, 526.3, 524.3, 524, 526.41, 525, 525.2, 526.1, 706.2, 528.4, 526.9, 526, 526.5, 527, 527.1, 527.2, 528.3, 528.7, 527.7, 527.8, 528.12, 528.11, 681.2, 528.5, 528.6, 529, 529.6, 530.1, 530.5, 530.3, 530, 530.6, 530.7, 531.2, 531.1, 531, 531.3, 531.4, 531.5, 535, 535.1, 535.8, 535.2, 535.6, 785, 537, 536, 540.1, 540, 550.1, 550.3, 550.4, 550.5, 550, 550.2, 555.1, 555.2, 990, 569.1, 561.1, 558, 441, 441.1, 441.2, 569, 560.1, 560.4, 560.2, 560.3, 562.1, 564.1, 564, 563, 565, 565.1, 578.8, 556, 567, 556.1, 568, 568.1, 571.8, 70.9, 573, 70.4, 571.5, 571.6, 573.1, 573.4, 574.1, 574.2, 574.3, 575, 575.7, 575.6, 575.8, 575.1, 575.2, 577.2, 577.3, 577, 557, 557.1, 569.2, 539, 853, 578.1, 578.2, 578.9
Ear disease death	380.1, 686.1, 385.3, 380, 380.4, 381.2, 381.3, 384.4, 384, 384.1, 385, 385.5, 383, 386, 386.1, 386.3, 389.3, 389, 389.2, 389.1, 382, 381.9, 389.4, 389.5, 388
Endocrine system disease death	244, 240, 241.2, 246, 348.7, 241.1, 241, 242, 242.1, 242.2, 253.1, 245, 245.1, 245.2, 250.1, 250.7, 443.7, 250.2, 260.2, 249, 251.1, 251, 251.8, 252.2, 275.5, 252.1, 252, 253.7, 253.2, 258, 253.3, 253, 255.1, 255.3, 255.21, 255, 256, 256.1, 256.4, 627.5, 257, 257.1, 259.3, 259.4, 259, 254, 259.2, 263, 260, 261.1, 261.2, 261.3, 261.4, 261, 262, 278.3, 278.1, 513.3, 278, 270.1, 277.5, 270.2, 271.3, 271.9, 331, 272.1, 272.9, 790.6, 277.1, 277.4, 275, 275.2, 275.1, 275.3, 499, 270.33, 276.5, 276.1, 276.4, 276.6, 258.1
Eye and adnexa disease death	371.3, 374, 374.1, 374.2, 374.3, 375, 375.2, 376, 371.9, 1001, 242.3, 369.5, 371.2, 372, 695.2, 371, 379, 379.1, 370, 370.1, 370.3, 364.1, 364, 364.2, 364.4, 364.5, 360, 371.1, 379.5, 366.2, 366, 366.1, 366.3, 379.3, 363, 363.3, 363.4, 361.1, 361.2, 361, 362, 362.1, 362.3, 362.29, 362.6, 362.7, 362.8, 365, 365.11, 365.2, 379.2, 360.2, 360.3, 377.3, 377, 377.1, 378.5, 378, 378.1, 367, 367.8, 367.1, 367.2, 368.3, 367.4, 368.7, 368, 368.1, 368.9, 368.2, 368.4, 368.5, 367.9, 378.2, 379.4, 379.9
Genitourinary system disease death	580.11, 580.12, 593, 580.14, 580.32, 269, 580.31, 590, 586.4, 599.1, 595, 586.12, 585.1, 585.3, 585.2, 594, 594.1, 594.3, 594.2, 594.8, 588.1, 586.11, 586.3, 586.2, 586, 592.1, 596.5, 596, 596.1, 592.2, 597.1, 592.3, 597, 618.1, 599, 591, 599.4, 600, 601.1, 602, 603.1, 603.2, 603, 601.3, 609.1, 604.1, 604, 601.4, 601.8, 608, 610.8, 610.1, 610.2, 610.3, 613.1, 612.2, 611.3, 613, 612.1, 613.5, 613.7, 613.8, 613.9, 614.3, 614.5, 614.4, 614, 614.1, 615, 618.2, 618.6, 618, 617, 619.1, 628, 624.2, 622.1, 622.2, 622, 621, 623, 674, 619.2, 626, 619.3, 624, 620, 619.4, 626.1, 624.1, 619.5, 627.4, 625, 626.2, 625.1, 626.4, 619, 627.1, 627.2, 627.3, 627, 634, 626.8, 80, 618.5, 597.2
Infectious and parasitic death	8, 8.5, 38.1, 8.7, 8.6, 10, 41, 686, 480.1, 369, 38.2, 711, 529.1, 994.2, 130, 79, 31.1, 31, 657, 639, 465, 41.2, 320, 420, 38, 133, 41.1, 90, 90.2, 90.3, 131, 54, 78, 528, 130.1, 136, 324, 324.1, 323, 386.2, 369.2, 79.1, 480.2, 53, 53.1, 381.1, 70.1, 70.2, 70, 70.3, 71.1, 112, 480.3, 230, 202.2, 195.1, 348.8, 504, 260.1, 798, 577.1, 79.2, 110.1, 110.2, 112.3, 420.3, 117.2, 117.1, 117.3, 117, 117.4, 580.2, 480, 134, 134.1, 132.1, 132, 41.4
Malignant neoplasms death	145.1, 145.2, 145.4, 145.5, 145, 149, 145.3, 149.1, 149.2, 149.3, 150, 151, 159.2, 153.2, 153.3, 153, 155, 155.1, 159.3, 157, 159, 149.9, 149.4, 165.1, 164, 165, 170.1, 172.11, 172.2, 159.4, 170.2, 174.1, 184.2, 184.1, 180.1, 182, 187.1, 185, 187.2, 189.11, 189.12, 189.4, 189.2, 190, 191.1, 193, 194, 195.3, 198.1, 198, 198.2, 198.3, 198.4, 198.7, 198.5, 198.6, 201, 204.4, 270.35, 200, 204, 204.1, 204.2, 204.3, 202, 277
Mental disorder death	290.1, 333.3, 290.3, 290.2, 291.4, 295.3, 291.1, 296, 300.1, 292.2, 290, 316, 317, 317.1, 318, 295.1, 296.22, 301.1, 295.2, 296.1, 301, 300.4, 300.3, 300.8, 300.9, 304, 303.1, 303, 295, 303.4, 303.3, 300, 306, 305.2, 327, 327.4, 327.1, 327.6, 327.5, 302, 302.1, 605, 301.2, 312.3, 315, 315.3, 315.2, 315.1, 313.3, 313.1, 312, 313, 313.2
Musculoskeletal and connective tissue disease death	711.1, 713, 716.9, 711.2, 446.3, 714, 714.2, 715.2, 274.1, 588, 274.2, 741, 716.3, 741.6, 716.8, 836, 716, 716.2, 740.9, 713.5, 740.2, 740.1, 736.2, 735.3, 735.2, 736, 739, 735.1, 735, 835, 742.2, 742.9, 742.1, 742.8, 728.2, 741.1, 742, 741.5, 741.4, 745, 741.2, 726.1, 446, 446.6, 446.4, 446.2, 446.8, 446.7, 446.5, 695.42, 709.5, 709.4, 709.2, 709.6, 711.3, 717, 772, 729.3, 709.7, 737.1, 721.8, 724.9, 737.2, 737.3, 732, 732.1, 738.4, 723.1, 737, 715, 715.3, 715.1, 710.19, 710, 721, 721.2, 721.1, 720, 743.4, 743.2, 724.8, 805, 722.7, 723, 765, 722.1, 722.6, 722.3, 760, 766, 761, 764, 770, 712, 728, 728.1, 727.7, 772.4, 772.1, 729, 727.1, 727, 727.5, 727.6, 727.4, 781, 727.2, 726.3, 728.7, 726, 726.2, 726.4, 729.1, 773, 771.1, 743.11, 733, 733.8, 733.2, 743.9, 710.11, 710.12, 733.4, 731.1, 710.3, 756.5, 732.7, 738, 858, 722.8, 769, 830
Neural system disease death	1011, 344, 323.8, 325, 334.1, 334.2, 357, 332, 349, 333.8, 333.4, 333, 333.1, 333.2, 331.9, 334, 335, 341, 323.2, 345, 345.1, 345.3, 340, 340.1, 339, 306.9, 433.3, 362.4, 433, 291.8, 327.3, 347, 352.1, 352.2, 352, 353, 353.1, 353.2, 722.9, 907, 351, 355, 356, 316.1, 250.6, 358.1, 358, 359.2, 359.1, 343, 342, 337, 337.1, 752.2, 331.1, 348.4, 348, 348.2, 798.1, 348.9, 967
Respiratory system disease death	464, 465.2, 474.1, 465.4, 483, 481, 1010, 480.5, 503, 519.8, 476, 472, 475, 471, 479, 470, 474.2, 474, 473.1, 473.3, 473, 497, 496.2, 496.1, 496, 495, 495.2, 496.3, 500.2, 500.1, 500, 501, 509.2, 505, 502, 506, 507, 509.3, 519.2, 509.1, 512, 508, 510, 513.8, 519, 714.1
Skin and subcutaneous disease	695.8, 686.2, 681, 681.1, 681.3, 681.5, 681.6, 681.7, 686.3, 686.5, 701.1, 702, 701, 695.1, 939, 690.1, 949.1, 930, 931, 695.9, 687.1, 695.7, 698, 705.1, 690, 696.3, 696.4, 696.2, 689, 947, 938.1, 938, 938.2, 702.1, 694.2, 703, 703.1, 731,

death	704.1, 704.8, 704, 704.2, 706.1, 695.3, 705.3, 705, 694.1, 702.2, 701.6, 700, 706.8, 707.1, 701.2, 701.4, 701.5, 702.4, 686.4, 695.41, 701.3, 709.3, 694.3, 707
Unnatural cause death	915, 916, 870.3, 870.1, 870.4, 870.2, 870.5, 819, 873, 817, 818, 1009, 1006, 872, 870.6, 952, 1007, 870, 1008, 809, 807, 803.3, 870.8, 802, 871, 803.1, 803, 803.2, 804, 840.3, 840, 800.2, 800.1, 800, 800.4, 800.3, 801, 871.4, 871.3, 801.1, 1015, 1000, 1014, 960, 960.2, 961.1, 961, 962, 962.3, 963, 965, 965.3, 969, 966, 971, 963.1, 964, 964.1, 972, 973, 975, 976, 974, 979, 981, 987, 983, 989, 985, 984, 986, 988, 913, 780, 1013, 946, 949, 783, 958, 958.2, 1012, 942, 941, 850, 958.1, 854, 81, 857, 859, 860, 510.2, 851, 874, 656.2, 297.2, 297, 960.1

The time of death and the underlying cause of death were recorded in the UK Biobank mortality data. The underlying cause of death coded according to ICD-10 were mapped to PheCodes, and categorized into 16 major causes of death.

Figure S1. Flowchart of study participants in PheWAS.

PheWAS = Phenome-wide association study; PSM = Propensity score matching.

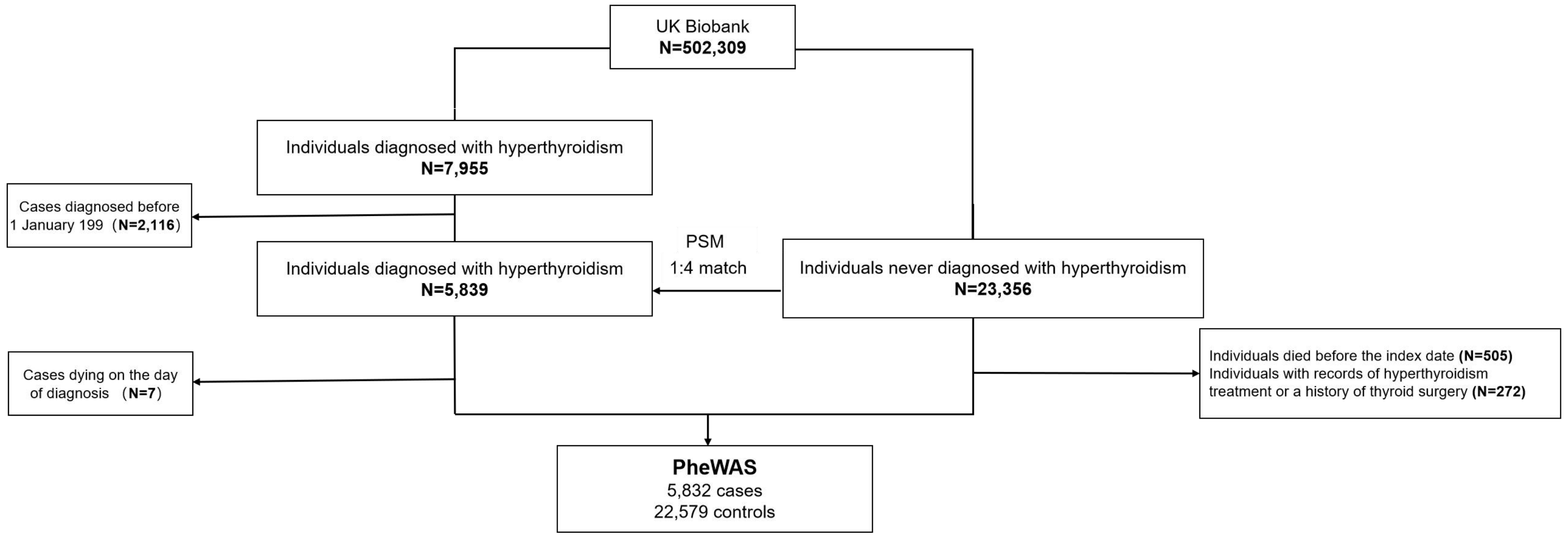

Figure S2. Results of the PheWAS analysis of hazard ratios for subsequent diseases among individuals with hyperthyroidism compared with matched controls.

The x-axis represents the estimated effect of prior diagnosed hyperthyroidism on subsequent conditions, and the y-axis represents the p-value for the associations. Each point corresponds to a phenotype that significantly associated with a prior diagosed hyperthyroidism after Bonferroni correction. The color of the point indicates disease categories by physiological system.

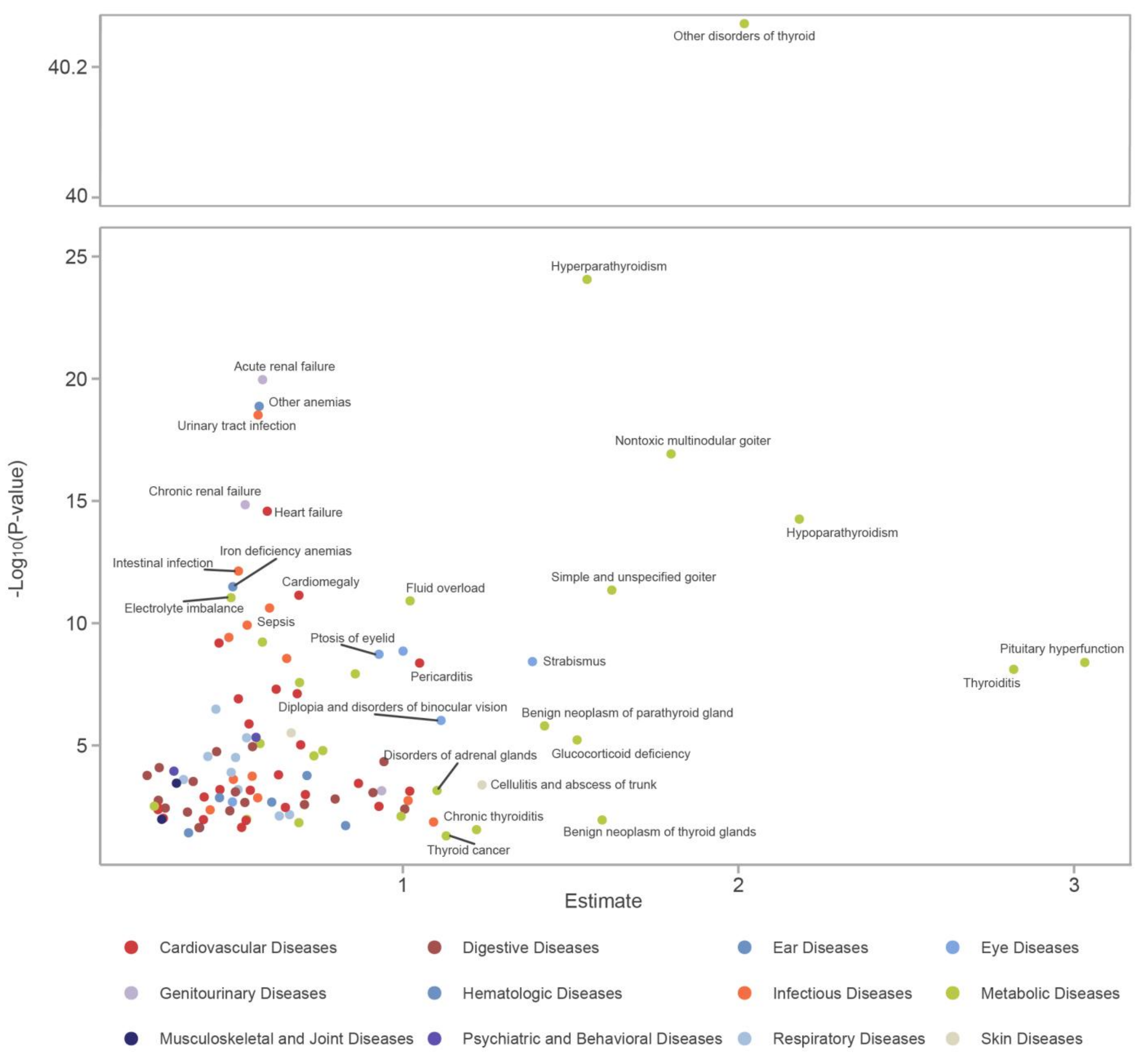

Table S2. Results of the PheWAS analysis of hazard ratios with 95% confidence intervals for subsequent diseases.

PheCode	Phenotype	HR (95% CI)	P-value	Time since diagnosis of hyperthyroidism, median (IQR), y	Cases/Participants[a]			Other Evidence[b]
					Overall	HT population	control population	
**Cardiovascular Diseases**								
427.2	Atrial fibrillation and flutter	1.32 (1.17-1.49)	4.19E-03	6.6 (2-13.4)	1,620/27,103	369/5,161	1,251/21,942	Naser et al.[2], Bekiaridou et al[3], Dekkers et al[4]
411.8	Other chronic ischemic heart disease, unspecified	1.57 (1.39-1.79)	6.44E-10	5.6 (2.1-11.2)	1,319/27,445	355/5,444	964/22,001	Sohn et al[5], Kim et al[6]
411.4	Coronary atherosclerosis	1.33 (1.17-1.52)	9.52E-03	6.8 (2-12.9)	1,242/27,373	300/5,449	942/21,924	Corana et al[7]
428	Heart failure	1.81 (1.58-2.08)	2.62E-15	5.9 (1.5-12.3)	1,048/27,915	318/5,584	730/22,331	Liu et al[8], Biondi et al[9]
411.2	Myocardial infarction	1.31 (1.12-1.53)	3.21E-03	7 (1.8-12.9)	902/27,514	215/5,506	687/22,008	Kim et al[6], Dekkers et al[4]
458.9	Hypotension NOS	1.67 (1.42-1.96)	1.23E-07	5 (1.1-11.5)	789/28,112	223/5,699	566/22,413	
426.3	Bundle branch block	1.73 (1.45-2.07)	1.31E-06	7.9 (3-13.7)	605/28,079	178/5,689	427/22,390	
433.3	Cerebral ischemia	1.59 (1.32-1.92)	6.37E-04	5.8 (1.6-13)	580/28,014	162/5,694	418/22,320	Dekkers et al[4], Squizzato et al[10], Sohn et al[5]
416	Cardiomegaly	2.02 (1.69-2.41)	7.17E-12	7.6 (2.7-12.8)	579/28,134	193/5,701	386/22,433	Biondi et al[9]
433.2	Occlusion of cerebral arteries	1.49 (1.23-1.8)	1.08E-02	6.5 (2.8-13.1)	566/27,848	153/5,653	413/22,195	Kim et al[6]
414	Other forms of chronic heart disease	1.88 (1.55-2.27)	4.99E-08	5.4 (1.6-12)	510/28,189	163/5,736	347/22,453	
458.1	Orthostatic hypotension	1.51 (1.22-1.87)	1.28E-03	5.6 (1.5-12)	443/28,244	119/5,759	324/22,485	
395.1	Nonrheumatic mitral valve disorders	1.99 (1.61-2.46)	7.62E-08	6.7 (2.4-12.7)	413/28,161	134/5,713	279/22,448	Channik et al[11]
426.2	Atrioventricular [AV] block	1.72 (1.38-2.16)	6.79E-04	5.3 (1.2-11)	389/28,200	115/5,745	274/22,455	Ata et al[12]
443.9	Peripheral vascular disease, unspecified	1.69 (1.32-2.17)	1.16E-02	8.3 (3.1-13.7)	314/28,204	92/5,752	222/22,452	Stuijver et al[13]
394	Rheumatic disease of the heart valves	2.02 (1.58-2.58)	9.44E-06	6.4 (1.6-12.4)	308/28,278	101/5,777	207/22,501	
394.2	Mitral valve disease	1.86 (1.46-2.37)	1.58E-04	5 (1.1-10.8)	308/28,269	99/5,775	209/22,494	Channik et al[11], Biondi et al[9]
427.1	Paroxysmal tachycardia, unspecified	1.69 (1.31-2.18)	2.24E-02	5.2 (1.7-10.6)	301/28,088	89/5,685	212/22,403	
415.2	Chronic pulmonary heart disease	1.92 (1.44-2.55)	3.41E-03	5.6 (1.5-12)	223/28,292	72/5,780	151/22,512	Araruna et al[14]
433.8	Late effects of cerebrovascular disease	1.99 (1.48-2.68)	1.02E-03	6.5 (2.4-11.1)	213/28,286	69/5,797	144/22,489	
420.2	Pericarditis	2.82 (2.09-3.82)	4.25E-09	5.6 (1.7-12.4)	182/28,267	75/5,782	107/22,485	Robinson et al[15], Gupta et al[16], Inami et al[17]
401.2	Hypertensive heart and/or renal disease	2.37 (1.68-3.35)	3.55E-04	5.3 (2.7-7.9)	146/28,253	55/5,768	91/22,485	Berta et al[18]
394.7	Disease of tricuspid valve	2.52 (1.68-3.78)	3.10E-03	7.2 (3.3-9.7)	107/28,345	39/5,817	68/22,528	
427.8	Sinoatrial node dysfunction (Bradycardia)	2.75 (1.81-4.19)	7.38E-04	7.3 (2-15)	95/28,330	38/5,810	57/22,520	
**Digestive Diseases**								
530.1	Esophagitis, GERD and related diseases	1.27 (1.16-1.4)	1.68E-04	6.7 (2.5-12.4)	2,780/25,324	639/4,958	2,141/20,366	Xu et al[19]
550.2	Diaphragmatic hernia	1.32 (1.19-1.47)	8.07E-05	7.6 (2.3-13.2)	2,112/26,189	507/5,214	1,605/20,975	
563	Constipation	1.33 (1.18-1.49)	1.75E-03	6 (2.1-13)	1,645/27,601	399/5,513	1,246/22,088	
535	Gastritis and duodenitis	1.34 (1.18-1.53)	3.63E-03	6.3 (2.6-11.8)	1,301/27,180	321/5,472	980/21,708	Ferrari et al[20], Tozzoli et al[21]
535.8	Other specified gastritis	1.45 (1.25-1.68)	2.97E-04	7.5 (2.7-13.3)	932/27,719	246/5,627	686/22,092	
578.9	Hemorrhage of gastrointestinal tract	1.45 (1.23-1.7)	5.30E-03	7.6 (2.7-13.1)	796/28,039	207/5,698	589/22,341	
558	Noninfectious gastroenteritis	1.57 (1.34-1.84)	1.78E-05	4.7 (1.6-9.7)	776/27,387	215/5,496	561/21,891	
785	Abdominal pain	1.47 (1.21-1.77)	2.29E-02	6.5 (3.1-10.8)	546/27,609	147/5,609	399/22,000	

571.5	Other chronic nonalcoholic liver disease	1.73 (1.42-2.1)	1.11E-05	7 (3.1-12.4)	508/28,162	149/5,743	359/22,419	
569	Other disorders of intestine	1.62 (1.3-2.01)	4.71E-03	6 (2.4-12.4)	417/28,149	119/5,752	298/22,397	
531.2	Gastric ulcer	1.71 (1.36-2.14)	2.14E-03	6.3 (1.5-12.9)	371/27,903	109/5,689	262/22,214	
550.5	Ventral hernia	1.66 (1.28-2.16)	7.91E-04	6.3 (3.1-11.6)	285/28,176	83/5,773	202/22,403	
575.8	Other disorders of biliary tract	2.08 (1.53-2.84)	2.61E-03	7.4 (2.6-13.9)	189/28,278	63/5,788	126/22,490	Daher et al[22]
557.1	Celiac disease	2.56 (1.8-3.64)	4.57E-05	12.5 (5.1-18.2)	143/28,213	53/5,767	90/22,446	Daher et al[22], Ferrar et ali[20]
577	Diseases of pancreas	2.47 (1.69-3.61)	8.47E-04	6.7 (1.8-11.6)	121/28,339	45/5,814	76/22,525	Nuovo et al[23]
555.1	Regional enteritis	2.2 (1.42-3.41)	1.54E-03	6.1 (2-12.4)	90/28,226	32/5,776	58/22,450	Xian et al[24], Bonapace and Srinivasan[25]
441	Vascular insufficiency of intestine	2.79 (1.78-4.37)	3.98E-03	7 (2-11.1)	88/28,352	35/5,823	53/22,529	
**Ear diseases**								
389	Hearing loss	1.44 (1.2-1.72)	3.73E-02	8.6 (2.3-14.2)	651/28,159	166/5,754	485/22,405	Wang et al[26]
**Ocular diseases**								
375	Disorders of lacrimal system	1.62 (1.24-2.12)	2.04E-03	8.2 (3.1-13.9)	272/28,157	78/5,756	194/22,401	Aydogan et al[27], Gürez et al[28]
374	Other disorders of eyelids	2.53 (1.94-3.29)	1.86E-09	4.5 (2.4-9.2)	243/28,152	93/5,750	150/22,402	Leonard et al[29]
374.3	Ptosis of eyelid	2.71 (2.05-3.6)	1.38E-09	5.7 (3.1-10.2)	215/28,228	83/5,767	132/22,461	Leonard et al[29]
368.2	Diplopia and disorders of binocular vision	3.08 (2.14-4.45)	9.46E-07	3.9 (1.6-8.9)	119/28,305	52/5,798	67/22,507	Leonard[2] et al[9]
378.1	Strabismus (not specified as paralytic)	3.98 (2.67-5.92)	3.71E-09	2.8 (1.3-4.6)	101/28,301	49/5,801	52/22,500	Leonard et al[29]
**Genitourinary Diseases**								
585.1	Acute renal failure	1.79 (1.59-2.01)	1.09E-20	7.7 (2.2-13.4)	1,467/27,936	440/5,579	1,027/22,357	You et al[30]
585.3	Chronic renal failure [CKD]	1.7 (1.51-1.92)	1.42E-15	8.7 (3.9-13.5)	1,416/27,797	402/5,524	1,014/22,273	You et al[30], Gopinatch et al[31]
580.2	Nephrotic syndrome without mention of glomerulonephritis	2.63 (1.62-4.26)	7.11E-04	8.6 (4.3-13.4)	76/28,329	30/5,799	46/22,530	Neves et al[32]
**Hematologic Diseases**								
285	Other anemias	1.79 (1.59-2.01)	1.34E-19	5.4 (1.5-10.9)	1,489/27,483	434/5,472	1,055/22,011	M'Rabet-Bensalah et al[33], Szczepanek-Parulska et al[34]
280.1	Iron deficiency anemias	1.63 (1.44-1.84)	3.22E-12	7 (2.5-12.7)	1,298/27,528	364/5,523	934/22,005	
288.11	Neutropenia	1.61 (1.26-2.06)	1.37E-03	4.6 (0.4-10.2)	323/28,183	91/5,738	232/22,445	
287.3	Thrombocytopenia	2.06 (1.56-2.72)	1.69E-04	3 (1.3-9.8)	230/28,276	78/5,787	152/22,489	Hymes et al[35]
281.1	Megaloblastic anemia	1.88 (1.35-2.61)	2.08E-03	5.6 (2.5-8.3)	172/28,206	54/5,758	118/22,448	Conrad et al[36]
285.2	Anemia of chronic disease	2.25 (1.51-3.36)	1.89E-02	6.9 (2.3-12.3)	110/28,327	39/5,803	71/22,524	
**Infectious Diseases**								
591	Urinary tract infection	1.77 (1.58-1.99)	3.05E-19	6.2 (1.8-12.6)	1,466/27,475	429/5,503	1,037/21,972	
8	Intestinal infection	1.66 (1.46-1.88)	7.36E-13	7.2 (2.2-13.7)	1,248/27,914	358/5,622	890/22,292	
480.1	Bacterial pneumonia	1.61 (1.41-1.84)	3.80E-10	5.5 (1.6-11.7)	1,141/27,930	323/5,631	818/22,299	
480	Pneumonia	1.71 (1.48-1.97)	1.19E-10	6.3 (1.9-12.4)	940/27,678	277/5,624	663/22,054	
994.2	Sepsis	1.86 (1.58-2.17)	2.39E-11	4.7 (0.9-12.2)	760/28,155	234/5,717	526/22,438	Hong et al[37]
41	Bacterial infection NOS	1.63 (1.35-1.99)	2.43E-04	4.3 (1.1-9.8)	530/27,782	150/5,652	380/22,130	
41.4	E. coli	1.93 (1.6-2.33)	2.76E-09	4.9 (1.8-11.4)	522/28,186	168/5,750	354/22,436	
79	Viral infection	1.54 (1.23-1.92)	4.29E-03	6.2 (2-14.5)	426/27,043	115/5,517	311/21,526	
112	Candidiasis	1.74 (1.4-2.15)	1.81E-04	4.2 (1-10.6)	419/28,220	124/5,755	295/22,465	
41.2	Streptococcus infection	1.76 (1.32-2.36)	1.38E-03	5.5 (2.1-12.4)	228/28,043	67/5,754	161/22,289	

136	Other infectious and parasitic diseases	2.8 (1.81-4.33)	1.79E-03	4.7 (2.6-11.2)	91/28,351	36/5,815	55/22,536	
481	Influenza	3 (1.79-5.05)	1.35E-02	5.7 (0.6-9.5)	64/28,332	26/5,811	38/22,521	
**Metabolic Diseases**								
244	Hypothyroid conditions	14.79 (13.18-16.59)	0.00E+00	4.5 (1.6-9.9)	2,243/25,346	1,516/4,174	727/21,172	Moroto et al[38], Metso et al[39]
250.2	Type 2 diabetes	1.3 (1.16-1.46)	3.04E-03	8 (3.3-13.8)	1,744/26,894	413/5,311	1,331/21,583	Popoviciu et al[40], Song et al[41]
276.1	Electrolyte imbalance	1.64 (1.44-1.85)	9.04E-12	6.6 (1.4-13.4)	1,283/27,953	362/5,595	921/22,358	Pompeo et al[42]
276.5	Hypovolemia	1.79 (1.52-2.1)	5.89E-10	5.1 (1.3-11.6)	744/28,133	224/5,709	520/22,424	
261.4	Vitamin D deficiency	1.77 (1.45-2.17)	8.34E-06	5.7 (1.7-12.5)	479/28,197	143/5,739	336/22,458	Vieira et al[43]
275.5	Disorders of calcium/phosphorus metabolism	2 (1.62-2.46)	2.67E-08	5.7 (1.8-12.9)	421/28,193	137/5,697	284/22,496	Popelier et al[44]
276.4	Acid-base balance disorder	1.68 (1.31-2.16)	1.05E-02	6 (1.8-12)	316/28,277	92/5,779	224/22,498	Kimmoun et al[45]
275.3	Disorders of magnesium metabolism	2.38 (1.84-3.06)	1.18E-08	5.3 (0.9-13.4)	267/28,330	101/5,805	166/22,525	Dolev et al[46]
261.2	Vitamin B-complex deficiencies	2.08 (1.59-2.72)	2.66E-05	6 (2.1-13.4)	252/28,271	86/5,777	166/22,494	Benites-Zapata et al[47]
276.6	Fluid overload	2.79 (2.14-3.64)	1.62E-05	5.6 (1.2-11.4)	244/28,294	84/5,779	160/22,515	
251.1	Hypoglycemia	2.12 (1.61-2.78)	1.22E-11	6.4 (1.5-12.7)	243/28,320	98/5,795	145/22,525	
246	Other disorders of thyroid	8.64 (6.39-11.67)	5.42E-41	3.4 (1-11.5)	212/28,192	142/5,694	70/22,498	
252.1	Hyperparathyroidism	4.68 (3.53-6.2)	8.64E-25	3 (0.9-8.3)	206/28,149	110/5,672	96/22,477	Bondeson et al[48]
250.1	Type 1 diabetes	2.02 (1.45-2.8)	1.44E-02	5.7 (2.5-10.4)	174/28,137	56/5,712	118/22,425	Wang et al[49], Ferrari et al[20]
241.2	Nontoxic multinodular goiter	8.24 (5.39-12.62)	1.18E-17	1.9 (0.7-6.6)	103/28,286	68/5,767	35/22,519	
240	Simple and unspecified goiter	6.68 (4.27-10.47)	4.42E-12	3.5 (0.7-7.1)	85/28,243	54/5,726	31/22,517	
241.1	Nontoxic uninodular goiter	2.74 (1.73-4.34)	7.91E-03	1.8 (1.1-5.4)	81/28,319	32/5,797	49/22,522	
252.2	Hypoparathyroidism	9.61 (5.72-16.13)	5.52E-15	4.8 (2.5-10.9)	74/28,351	51/5,817	23/22,534	
227.2	Benign neoplasm of parathyroid gland	4.26 (2.64-6.86)	1.59E-06	0.8 (0.3-3.3)	70/28,311	36/5,793	34/22,518	
255.21	Glucocorticoid deficiency	4.55 (2.69-7.69)	5.95E-06	3.6 (0.4-9.5)	56/28,331	30/5,806	26/22,525	
255	Disorders of adrenal glands	2.96 (1.68-5.2)	6.94E-04	7.2 (2.2-17)	52/28,350	23/5,817	29/22,533	Silva et al[50]
193	Thyroid cancer	4.21 (2.26-7.83)	4.97E-02	1.3 (0.4-2.5)	42/28,322	21/5,795	21/22,527	Yuan et al[51], Kitahara et al[52], Tran et al[53]
245.2	Chronic thyroiditis	4.99 (2.55-9.76)	4.01E-09	2.9 (0.8-8.8)	39/28,340	32/5,807	7/22,533	
253.1	Pituitary hyperfunction	20.45 (8.54-48.94)	7.70E-09	2.2 (0.9-4.7)	36/28,306	30/5,791	6/22,515	
245	Thyroiditis	18.99 (7.9-45.66)	2.77E-02	1.8 (1-4.5)	35/28,340	20/5,809	15/22,531	Conrad et al[36]
226	Benign neoplasm of thyroid glands	5.82 (2.7-12.54)	1.11E-02	1.5 (0.6-3.4)	29/28,340	16/5,811	13/22,529	
**Musculoskeletal And Joint Diseases**								
743.11	Osteoporosis NOS	1.39 (1.22-1.58)	1.05E-02	8 (3.2-13.1)	1,281/26,955	311/5,426	970/21,529	Qi et al[54]
760	Back pain	1.32 (1.15-1.5)	3.49E-04	6.4 (2.1-12.5)	1271/27,732	322/5,526	949/22,206	
721	Spondylosis and allied disorders	1.49 (1.23-1.8)	2.29E-02	6.3 (2.5-13.1)	545/27,869	147/5,680	398/22,189	Rosen et al[55]
**Psychiatric And Behavioral Diseases**								
300.1	Anxiety disorder	1.38 (1.22-1.55)	1.12E-04	7.7 (3.1-14.1)	1,482/27,687	363/5,550	1119/22,137	Kitahara et al[56], Brandt et al[57], Holmberg et al[58], Zader et al[59]
290.2	Delirium due to conditions classified elsewhere	1.76 (1.45-2.14)	4.64E-06	6.3 (1.6-13.6)	524/28,269	154/5,769	370/22,500	Goldfarb et al[60]
**Respiratory Diseases**								
496	Chronic airway obstruction	1.4 (1.23-1.61)	2.47E-04	7.4 (2.8-12.9)	1,156/27,751	291/5,573	865/22,178	
507	Pleurisy; pleural effusion	1.56 (1.35-1.8)	3.27E-07	4.4 (1.4-10.9)	1,008/28,027	278/5,673	730/22,354	

519.8	Other diseases of respiratory system, NEC	1.51 (1.3-1.76)	2.81E-05	4.6 (2-11.7)	877/27,819	236/5,591	641/22,228	Brüssel et al[61]
508	Pulmonary collapse; interstitial and compensatory emphysema	1.69 (1.4-2.03)	3.10E-05	5.1 (1.4-12.9)	592/28,182	169/5,738	423/22,444	
509.1	Respiratory failure	1.69 (1.41-2.03)	4.84E-06	6.1 (1.2-12.4)	568/28,231	168/5,757	400/22,474	
496.2	Chronic bronchitis	1.61 (1.33-1.94)	1.26E-04	8 (2.9-13.7)	561/28,018	160/5,695	401/22,323	
327.3	Sleep apnea	1.67 (1.36-2.06)	6.42E-04	7.9 (3.5-14.1)	443/28,065	129/5,726	314/22,339	
479	Other upper respiratory disease	1.88 (1.4-2.51)	7.64E-03	4.9 (1.4-9)	226/28,055	69/5,734	157/22,321	Li Pi Shan et al[62]
501	Pneumonitis due to inhalation of food or vomitus	1.95 (1.44-2.64)	6.73E-03	5.4 (1.4-15.5)	212/28,342	67/5,812	145/22,530	
**Skin Diseases**								
707.1	Decubitus ulcer	1.93 (1.54-2.42)	3.05E-06	6.2 (2.3-13)	371/28,301	117/5,788	254/22,513	
681.7	Cellulitis and abscess of trunk	3.49 (2.12-5.75)	4.11E-04	8.4 (3.6-13.7)	67/28,332	30/5,819	37/22,513	

HT = Hyperthyroidism; HR = Hazard ratio; CI = Confidence interval.

All p-value reported has been adjusted using Bonferroni correction.

[a]For each analysis, individuals who had previously been diagnosed with the analyzed phenotype before the index date were excluded. The 'Cases/Participants' column lists the number of participants analyzed for each phenotype and the number of those who developed the condition.

[b]For each phenotype significantly associated in the PheWAS, we searched PubMed using search terms such as 'hyperthyroidism AND osteoporosis' to investigate whether any previous research has reported on it.

Table S3. Associations between prior diagnosed hyperthyroidism and causes of death.

Cause of death	Both sex (N = 28,411, HT cases = 5,832)			Males (N = 6,549, HT cases = 1,337)			Females (N = 21,862, HT cases = 4,495)		
	N (HT population)	HR (95% CI)	P-value	N (HT population)	HR (95% CI)	P-value	N (HT population)	HR (95% CI)	P-value
All-cause mortality	766	1.87 (1.71-2.04)	4.65E-45	261	2.25 (1.88-2.68)	2.78E-19	505	1.81 (1.63-2.02)	2.20E-27
Malignant neoplasms death	319	1.49 (1.3-1.7)	5.20E-09	78	1.23 (0.94-1.62)	1.30E-01	241	1.59 (1.37-1.86)	3.20E-09
Cardiovascular disease death	162	1.82 (1.49-2.21)	3.10E-09	84	2.38 (1.8-3.14)	1.20E-09	78	1.47 (1.11-1.94)	6.50E-03
Respiratory system disease death	79	1.76 (1.33-2.35)	9.50E-05	28	2.03 (1.25-3.29)	4.30E-03	51	1.64 (1.15-2.33)	6.10E-03
Neural system disease death	43	1.11 (0.77-1.59)	5.80E-01	14	1.04 (0.55-1.97)	9.00E-01	29	1.15 (0.74-1.79)	5.50E-01
Digestive system disease death	40	2.03 (1.36-3.04)	5.20E-04	16	2.51 (1.32-4.77)	5.10E-03	24	1.76 (1.05-2.96)	3.30E-02
Mental disorder death	26	1.74 (1.06-2.84)	6.80E-02	9	-	-	17	1.66 (0.92-3.02)	9.40E-02

HT = Hyperthyroidism.

- = The model was unable to accurately estimate the effect due to an insufficient sample size.

Stratified Cox regression was performed to examine the association between hyperthyroidism and all-cause mortality. The Fine-Gray competing risk model was used to assess the impact of hyperthyroidism on specific causes of death.

All p-value reported has been adjusted using Bonferroni correction.

Figure S3. Cumulative hazard of multisystem diseases after prior diagnosed hyperthyroidism.

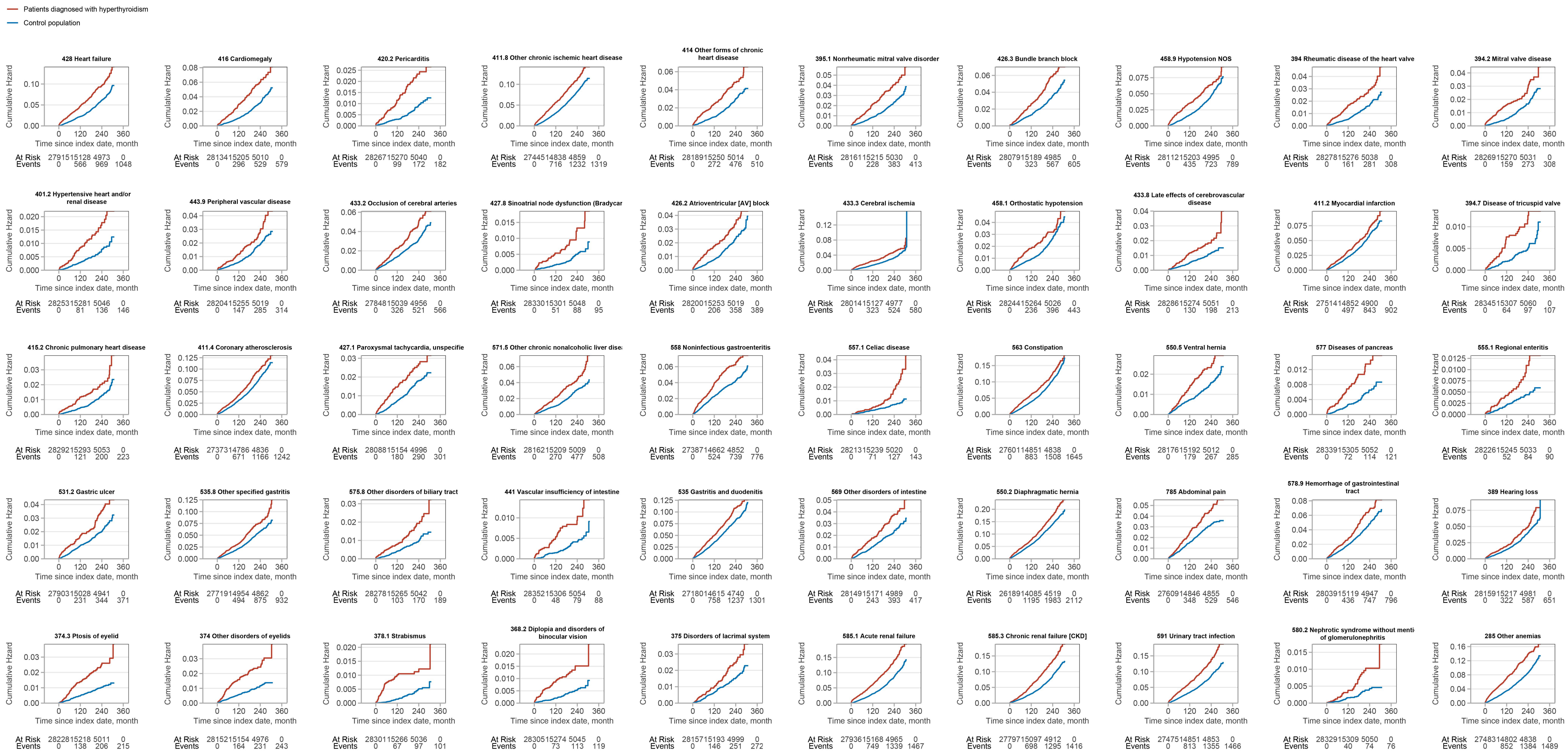

Figure S3 (continued)

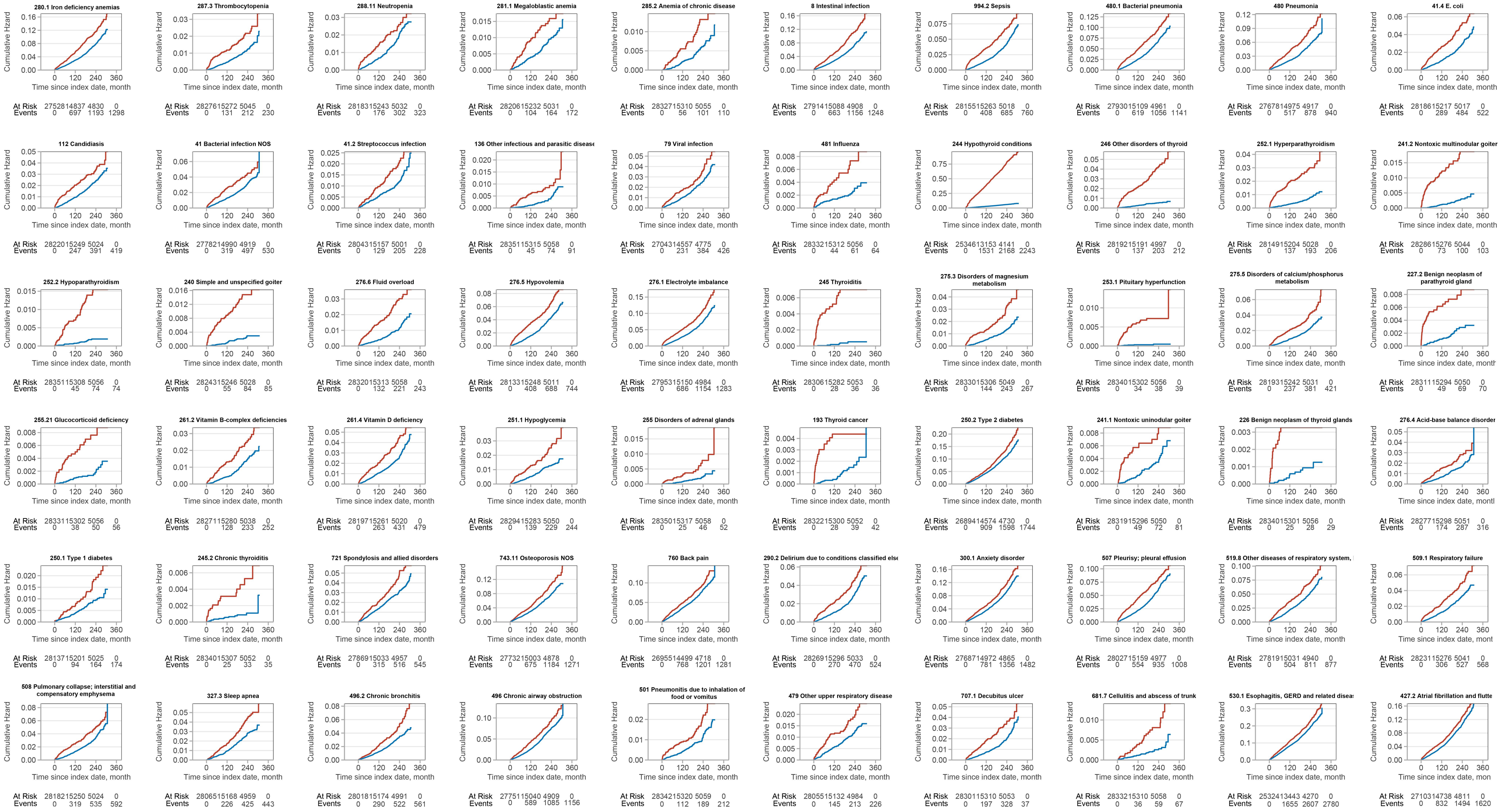

Figure S4. Cumulative hazard of mortality after prior diagnosed hyperthyroidism.

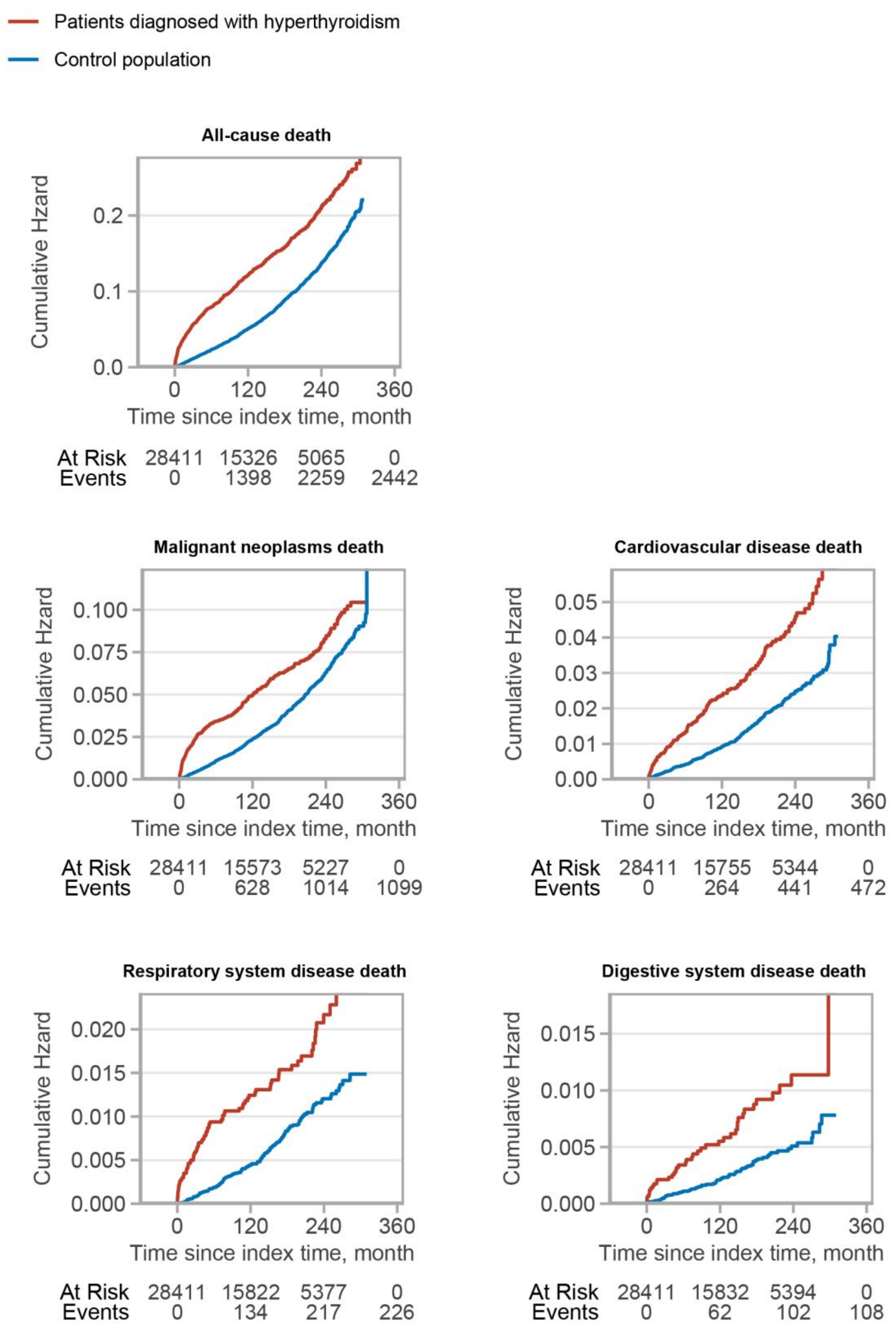

Landmark Analysis

Table S4. Results of landmark analyses to investigate the time-varying effect of prior diagnosed hyperthyroidism on subsequent diseases.

PheCode	Phenotype	Within 2 years since hyperthyroidism		2 to 5 year since hyperthyroidism		5 to10 year since hyperthyroidism		Beyond 10 years since hyperthyroidism	
		Cases/Participants (HT population)	HR (95% CI)	Cases/Participants (HT population)	HR (95% CI)	Cases/Participants (HT population)	HR (95% CI)	Cases/Participants (HT population)	HR (95% CI)
**Cardiovascular Diseases**									
427.2	Atrial fibrillation and flutter	71/5,161	2.36 (1.74-3.2)	64/4,459	1.53 (1.14-2.06)	73/3,707	1.21 (0.93-1.58)	161/2,793	1.09 (0.92-1.31)
411.8	Other chronic ischemic heart disease, unspecified	70/5,444	3.64 (2.61-5.08)	65/4,664	1.93 (1.42-2.61)	89/3,866	1.66 (1.29-2.14)	131/2,863	1.1 (0.9-1.34)
411.4	Coronary atherosclerosis	59/5,449	2.57 (1.84-3.58)	40/4,667	1.06 (0.74-1.5)	73/3,885	1.32 (1.01-1.73)	128/2,875	1.17 (0.96-1.43)
428	Heart failure	70/5,584	3.44 (2.47-4.79)	53/4,796	2.12 (1.51-2.98)	74/3,971	1.94 (1.46-2.58)	121/2,948	1.32 (1.07-1.63)
411.2	Myocardial infarction	45/5,506	2.37 (1.62-3.48)	30/4,709	1.1 (0.73-1.66)	56/3,904	1.44 (1.06-1.97)	84/2,892	1.06 (0.83-1.35)
458.9	Hypotension NOS	64/5,699	5.02 (3.44-7.32)	40/4,890	1.98 (1.34-2.91)	51/4,036	1.7 (1.22-2.38)	68/2,991	0.96 (0.73-1.25)
426.3	Bundle branch block	26/5,689	2.33 (1.42-3.82)	31/4,875	1.74 (1.14-2.67)	49/4,029	2.29 (1.6-3.27)	72/2,980	1.37 (1.04-1.8)
433.3	Cerebral ischemia	41/5,694	4.12 (2.63-6.47)	29/4,868	1.61 (1.04-2.49)	36/4,026	1.51 (1.02-2.25)	56/2,982	1.12 (0.82-1.51)
416	Cardiomegaly	36/5,701	4.07 (2.54-6.54)	31/4,879	2.34 (1.49-3.67)	54/4,029	2.58 (1.81-3.68)	72/2,986	1.36 (1.04-1.79)
433.2	Occlusion of cerebral arteries	27/5,653	1.71 (1.08-2.7)	25/4,841	1.57 (0.98-2.51)	41/3,996	1.66 (1.14-2.41)	60/2,958	1.29 (0.96-1.75)
414	Other forms of chronic heart disease	38/5,736	4.29 (2.68-6.84)	35/4,900	2.37 (1.54-3.64)	33/4,048	2.02 (1.31-3.12)	57/3,007	1.21 (0.89-1.64)
458.1	Orthostatic hypotension	29/5,759	3.35 (2.01-5.59)	23/4,933	1.66 (1.01-2.73)	28/4,074	1.88 (1.19-2.98)	39/3,012	0.93 (0.65-1.34)
395.1	Nonrheumatic mitral valve disorders	27/5,713	3.51 (2.08-5.94)	23/4,875	2.78 (1.62-4.77)	37/4,024	2.38 (1.58-3.6)	47/2,987	1.29 (0.92-1.81)
426.2	Atrioventricular [AV] block	28/5,745	3.98 (2.33-6.8)	23/4,903	2.67 (1.56-4.58)	26/4,052	1.83 (1.14-2.92)	38/3,004	1.02 (0.7-1.47)
443.9	Peripheral vascular disease, unspecified	15/5,752	2.46 (1.23-4.9)	16/4,926	2.69 (1.41-5.14)	18/4,063	1.89 (1.07-3.33)	43/3,005	1.31 (0.92-1.87)
394	Rheumatic disease of the heart valves	22/5,777	3.51 (1.95-6.31)	16/4,932	1.99 (1.09-3.66)	32/4,071	3.81 (2.29-6.33)	31/3,013	1.15 (0.77-1.72)
394.2	Mitral valve disease	25/5,775	4.92 (2.7-8.95)	18/4,936	2.38 (1.31-4.34)	27/4,072	3 (1.8-5)	29/3,009	0.91 (0.61-1.37)
427.1	Paroxysmal tachycardia, unspecified	20/5,685	3.54 (1.93-6.49)	19/4,858	2.68 (1.49-4.83)	23/3,999	1.47 (0.9-2.42)	27/2,953	1.09 (0.71-1.68)
415.2	Chronic pulmonary heart disease	17/5,780	6.12 (2.87-13.07)	11/4,943	1.93 (0.92-4.03)	21/4,080	2.57 (1.47-4.5)	23/3,018	1.08 (0.68-1.73)
433.8	Late effects of cerebrovascular disease	9/5,797	1.58 (0.72-3.46)	15/4,955	2.54 (1.32-4.9)	23/4,081	2.58 (1.49-4.48)	22/3,015	1.58 (0.96-2.59)
420.2	Pericarditis	15/5,782	4.11 (1.98-8.52)	14/4,938	3.58 (1.72-7.42)	19/4,067	4.27 (2.17-8.38)	27/3,009	1.86 (1.17-2.96)
401.2	Hypertensive heart and/or renal disease	9/5,768	5.54 (1.96-15.62)	8/4,928	2.14 (0.9-5.1)	17/4,061	2.96 (1.57-5.57)	21/3,014	1.68 (0.97-2.91)
394.7	Disease of tricuspid valve	7/5,817	3.3 (1.2-9.13)	6/4,964	2.88 (1-8.3)	17/4,090	5.35 (2.55-11.21)	9/3,019	1.1 (0.52-2.32)
427.8	Sinoatrial node dysfunction (Bradycardia)	9/5,810	4.35 (1.68-11.29)	5/4,956	3.67 (1.06-12.71)	9/4,082	2.88 (1.21-6.84)	15/3,020	2.06 (1.1-3.89)
**Digestive Diseases**									
530.1	Esophagitis, GERD and related diseases	112/4,958	1.74 (1.38-2.19)	124/4,232	1.37 (1.11-1.69)	165/3,520	1.29 (1.08-1.55)	238/2,601	1.08 (0.93-1.25)
550.2	Diaphragmatic hernia	105/5,214	2.08 (1.62-2.65)	82/4,434	1.27 (0.98-1.64)	108/3,684	1.1 (0.88-1.36)	212/2,731	1.25 (1.07-1.47)
563	Constipation	77/5,513	2.45 (1.83-3.28)	81/4,748	1.76 (1.34-2.3)	92/3,937	1.32 (1.04-1.68)	149/2,918	0.97 (0.8-1.16)
535	Gastritis and duodenitis	65/5,472	2.28 (1.67-3.12)	58/4,674	1.47 (1.08-2)	78/3,851	1.14 (0.88-1.47)	120/2,849	1.17 (0.95-1.44)
535.8	Other specified gastritis	41/5,627	1.89 (1.29-2.77)	45/4,824	1.72 (1.2-2.45)	49/3,973	1.25 (0.9-1.73)	111/2,942	1.34 (1.08-1.67)
578.9	Hemorrhage of gastrointestinal tract	33/5,698	2.05 (1.34-3.13)	42/4,873	1.71 (1.19-2.47)	42/4,015	1.22 (0.86-1.73)	90/2,977	1.32 (1.03-1.68)

558	Noninfectious gastroenteritis	50/5,496	2.6 (1.81-3.74)	43/4,678	1.31 (0.93-1.86)	62/3,833	1.67 (1.24-2.25)	60/2,833	1.26 (0.94-1.69)
785	Abdominal pain	22/5,609	1.46 (0.89-2.39)	31/4,784	1.75 (1.14-2.68)	43/3,929	1.37 (0.97-1.95)	51/2,897	1.41 (1.02-1.95)
571.5	Other chronic nonalcoholic liver disease	27/5,743	2.37 (1.46-3.84)	28/4,910	1.83 (1.17-2.89)	35/4,041	2.1 (1.38-3.18)	59/2,998	1.38 (1.02-1.86)
569	Other disorders of intestine	23/5,752	2.51 (1.48-4.25)	21/4,904	1.39 (0.84-2.29)	31/4,038	1.79 (1.16-2.75)	44/2,985	1.37 (0.97-1.95)
531.2	Gastric ulcer	29/5,689	3.56 (2.13-5.95)	18/4,845	1.77 (1.01-3.1)	25/4,000	1.25 (0.79-1.96)	37/2,954	1.45 (0.99-2.13)
550.5	Ventral hernia	15/5,773	1.66 (0.91-3.06)	16/4,922	1.47 (0.82-2.63)	24/4,046	2.27 (1.37-3.77)	28/2,996	1.44 (0.93-2.23)
575.8	Other disorders of biliary tract	12/5,788	3.17 (1.46-6.87)	13/4,945	3.56 (1.67-7.58)	10/4,069	1.03 (0.51-2.09)	28/3,019	2.11 (1.33-3.36)
557.1	Celiac disease	5/5,767	2.22 (0.74-6.63)	7/4,920	1.94 (0.78-4.82)	9/4,054	1.64 (0.75-3.58)	32/2,995	3.47 (2.13-5.66)
577	Diseases of pancreas	11/5,814	5.52 (2.13-14.3)	4/4,964	1.21 (0.39-3.71)	14/4,090	2.94 (1.46-5.91)	16/3,023	1.99 (1.09-3.66)
555.1	Regional enteritis	4/5,776	2.51 (0.71-8.93)	6/4,936	2.11 (0.78-5.7)	8/4,072	2.2 (0.92-5.24)	14/3,016	2.16 (1.11-4.23)
441	Vascular insufficiency of intestine	7/5,823	8.31 (2.14-32.31)	7/4,969	2.92 (1.08-7.88)	9/4,089	4.88 (1.81-13.12)	12/3,023	1.54 (0.76-3.14)
**Ear diseases**									
389	Hearing loss	30/5,754	2.4 (1.52-3.79)	24/4,919	1.42 (0.89-2.29)	32/4,058	1.33 (0.89-1.99)	80/3,013	1.28 (0.99-1.66)
**Ocular Diseases**									
375	Disorders of lacrimal system	11/5,756	2.36 (1.11-5.01)	14/4,909	1.75 (0.93-3.3)	16/4,039	1.13 (0.64-1.97)	37/2,987	1.74 (1.18-2.58)
374	Other disorders of eyelids	17/5,750	2.43 (1.31-4.48)	29/4,901	4.63 (2.69-7.96)	22/4,013	2.14 (1.27-3.61)	25/2,957	1.87 (1.15-3.04)
374.3	Ptosis of eyelid	12/5,767	3.03 (1.42-6.49)	21/4,924	4.08 (2.21-7.54)	25/4,041	2.56 (1.54-4.25)	25/2,981	2.13 (1.3-3.48)
368.2	Diplopia and disorders of binocular vision	14/5,798	5.33 (2.36-12.02)	14/4,941	5.03 (2.28-11.09)	11/4,062	3.1 (1.38-6.93)	13/3,002	1.63 (0.85-3.14)
378.1	Strabismus (not specified as paralytic)	15/5,801	19.71 (5.7-68.11)	20/4,941	16 (6-42.63)	10/4,057	2.93 (1.28-6.68)	4/2,997	0.57 (0.2-1.63)
**Genitourinary Diseases**									
585.1	Acute renal failure	90/5,579	3.57 (2.66-4.79)	62/4,819	1.72 (1.27-2.33)	102/4,008	1.95 (1.53-2.48)	186/2,972	1.4 (1.18-1.66)
585.3	Chronic renal failure [CKD]	46/5,524	2.01 (1.4-2.9)	63/4,785	2 (1.47-2.72)	109/3,959	2.09 (1.65-2.66)	184/2,951	1.42 (1.19-1.69)
580.2	Nephrotic syndrome without mention of glomerulonephritis	3/5,799	3 (0.67-13.4)	3/4,954	3.81 (0.77-18.94)	9/4,082	2.14 (0.84-5.46)	15/3,022	2.67 (1.37-5.19)
**Hematologic Diseases**									
285	Other anemias	108/5,472	3.73 (2.85-4.89)	80/4,685	2.2 (1.66-2.9)	101/3,864	1.69 (1.33-2.15)	145/2,863	1.22 (1-1.47)
280.1	Iron deficiency anemias	63/5,523	2.42 (1.75-3.33)	60/4,730	1.99 (1.45-2.73)	96/3,897	1.77 (1.39-2.27)	145/2,868	1.27 (1.05-1.54)
288.11	Neutropenia	27/5,738	4.38 (2.52-7.59)	17/4,903	1.79 (1-3.21)	19/4,046	1.62 (0.95-2.77)	28/3,000	0.96 (0.63-1.45)
287.3	Thrombocytopenia	27/5,787	6.98 (3.65-13.34)	14/4,945	2.25 (1.15-4.38)	13/4,079	1.5 (0.79-2.84)	24/3,018	1.27 (0.8-2.03)
281.1	Megaloblastic anemia	12/5,758	3.61 (1.65-7.91)	10/4,915	2.23 (1.02-4.87)	17/4,046	2.21 (1.21-4.02)	15/2,995	1.12 (0.63-2.01)
285.2	Anemia of chronic disease	7/5,803	26.21 (3.22-213.34)	6/4,951	2.36 (0.86-6.49)	10/4,079	2.13 (0.98-4.62)	16/3,021	1.62 (0.89-2.94)
**Infectious Diseases**									
591	Urinary tract infection	86/5,503	2.82 (2.13-3.75)	80/4,715	2.06 (1.57-2.71)	97/3,891	1.72 (1.35-2.19)	166/2,883	1.43 (1.19-1.71)
8	Intestinal infection	65/5,622	2.36 (1.72-3.23)	52/4,827	1.79 (1.28-2.5)	91/3,991	1.72 (1.34-2.21)	150/2,963	1.41 (1.16-1.7)
480.1	Bacterial pneumonia	79/5,631	3.96 (2.87-5.46)	60/4,828	2.15 (1.56-2.97)	69/4,001	1.38 (1.04-1.83)	115/2,971	1.1 (0.89-1.36)
480	Pneumonia	57/5,624	2.47 (1.77-3.46)	51/4,831	2.21 (1.56-3.14)	64/3,994	1.61 (1.19-2.18)	105/2,961	1.36 (1.09-1.71)
994.2	Sepsis	74/5,717	5.33 (3.73-7.6)	35/4,896	2 (1.32-3.02)	49/4,057	1.76 (1.25-2.49)	76/3,013	1.13 (0.87-1.47)
41	Bacterial infection NOS	37/5,652	2.98 (1.93-4.6)	29/4,835	1.95 (1.24-3.04)	42/3,986	1.91 (1.3-2.81)	42/2,956	0.99 (0.7-1.39)
41.4	E. coli	33/5,750	3.23 (2.02-5.15)	40/4,922	2.81 (1.86-4.23)	40/4,047	2.26 (1.53-3.36)	55/2,988	1.22 (0.9-1.65)
79	Viral infection	28/5,517	2.99 (1.8-4.96)	19/4,703	1.61 (0.93-2.77)	25/3,873	1.6 (1-2.56)	43/2,867	1.13 (0.8-1.6)

112	Candidiasis	42/5,755	5.66 (3.5-9.13)	18/4,926	1.52 (0.88-2.64)	30/4,062	1.75 (1.13-2.7)	34/3,008	0.96 (0.66-1.41)
41.2	Streptococcus infection	14/5,754	2.36 (1.2-4.61)	8/4,919	1.13 (0.51-2.49)	19/4,057	2.25 (1.27-3.97)	26/3,000	1.57 (0.99-2.49)
136	Other infectious and parasitic diseases	7/5,815	6.66 (1.95-22.77)	11/4,968	13.84 (3.86-49.69)	8/4,092	2.95 (1.16-7.49)	10/3,026	1.18 (0.58-2.42)
481	Influenza	9/5,811	4.05 (1.55-10.53)	3/4,958	1.46 (0.39-5.51)	7/4,093	4.98 (1.57-15.72)	7/3,025	2.41 (0.93-6.23)
**Metabolic Diseases**									
244	Hypothyroid conditions	441/4,174	21.79 (17-27.93)	289/3,264	11.78 (9.28-14.95)	351/2,520	15.27 (12-19.43)	435/1,734	12.43 (10.14-15.24)
250.2	Type 2 diabetes	50/5,311	1.51 (1.08-2.11)	77/4,587	1.49 (1.14-1.94)	86/3,795	1.1 (0.86-1.4)	200/2,838	1.3 (1.11-1.54)
276.1	Electrolyte imbalance	90/5,595	4.08 (3-5.53)	56/4,823	1.58 (1.15-2.17)	74/3,996	1.45 (1.1-1.91)	142/2,973	1.25 (1.03-1.52)
276.5	Hypovolemia	58/5,709	4.59 (3.11-6.76)	41/4,901	2.62 (1.75-3.93)	52/4,049	1.61 (1.16-2.24)	73/3,004	1.12 (0.86-1.46)
261.4	Vitamin D deficiency	36/5,739	5.47 (3.28-9.12)	24/4,907	1.77 (1.08-2.89)	29/4,055	1.46 (0.94-2.24)	54/3,016	1.32 (0.96-1.81)
275.5	Disorders of calcium/phosphorus metabolism	31/5,697	4.39 (2.62-7.37)	30/4,884	3.55 (2.15-5.84)	29/4,023	1.64 (1.06-2.53)	47/2,981	1.32 (0.94-1.84)
276.4	Acid-base balance disorder	19/5,779	2.81 (1.55-5.08)	16/4,949	2.38 (1.27-4.46)	29/4,077	2.37 (1.47-3.83)	28/3,022	0.96 (0.63-1.47)
275.3	Disorders of magnesium metabolism	33/5,805	6.53 (3.65-11.67)	15/4,952	2.91 (1.48-5.75)	13/4,080	1.22 (0.65-2.29)	40/3,026	1.86 (1.26-2.74)
261.2	Vitamin B-complex deficiencies	18/5,777	5.1 (2.44-10.67)	16/4,939	2.87 (1.49-5.53)	22/4,068	2.6 (1.49-4.53)	30/3,010	1.27 (0.84-1.93)
276.6	Fluid overload	20/5,779	5.22 (2.63-10.37)	14/4,943	2.14 (1.1-4.17)	21/4,080	2.17 (1.25-3.75)	29/3,018	1.48 (0.96-2.28)
251.1	Hypoglycemia	25/5,795	6.05 (3.18-11.51)	16/4,956	2.81 (1.45-5.44)	24/4,091	4.7 (2.55-8.69)	33/3,026	1.65 (1.09-2.49)
246	Other disorders of thyroid	50/5,694	20.65 (10.15-42.04)	26/4,827	8.23 (4.15-16.33)	19/3,965	4.58 (2.32-9.05)	47/2,926	6.95 (4.27-11.31)
252.1	Hyperparathyroidism	41/5,672	11.93 (6.39-22.28)	22/4,834	6.86 (3.39-13.88)	24/3,980	4.37 (2.43-7.86)	23/2,949	1.99 (1.19-3.31)
250.1	Type 1 diabetes	6/5,712	1.39 (0.55-3.53)	15/4,876	3.72 (1.81-7.62)	9/4,014	1.32 (0.59-2.95)	26/2,978	2.04 (1.26-3.28)
241.2	Nontoxic multinodular goiter	31/5,767	120.1 (16.39-879.97)	13/4,908	9.76 (3.47-27.45)	11/4,046	4.62 (1.91-11.16)	13/2,992	2.93 (1.41-6.1)
240	Simple and unspecified goiter	18/5,726	35.54 (8.24-153.2)	13/4,887	9.3 (3.27-26.41)	9/4,018	4.96 (1.85-13.35)	14/2,964	3.11 (1.51-6.4)
241.1	Nontoxic uninodular goiter	16/5,797	14.75 (4.93-44.19)	5/4,935	2.72 (0.86-8.59)	6/4,072	2.51 (0.89-7.08)	5/3,012	0.78 (0.3-2.05)
252.2	Hypoparathyroidism	9/5,817	8.63 (2.65-28.06)	16/4,963	15.58 (5.21-46.63)	7/4,080	8.84 (2.28-34.23)	19/3,018	7.68 (3.46-17.03)
227.2	Benign neoplasm of parathyroid gland	21/5,793	19.46 (6.67-56.79)	7/4,928	4.47 (1.5-13.31)	3/4,061	1.66 (0.43-6.44)	5/3,009	1.27 (0.46-3.51)
255.21	Glucocorticoid deficiency	10/5,806	20 (4.38-91.28)	8/4,955	8 (2.41-26.57)	5/4,080	2.18 (0.73-6.5)	7/3,018	2.46 (0.95-6.36)
255	Disorders of adrenal glands	6/5,817	21.37 (2.57-177.96)	1/4,969	2 (0.18-22.06)	6/4,094	2.37 (0.84-6.68)	10/3,028	2.19 (0.96-5.02)
193	Thyroid cancer	14/5,795	53.16 (6.99-404.57)	3/4,937	3.81 (0.77-18.94)	4/4,068	7.64 (1.4-41.8)	0/3,008	-
245.2	Chronic thyroiditis	13/5,807	48.2 (6.29-369.1)	10/4,953	12.79 (3.52-46.54)	5/4,073	10 (1.94-51.54)	4/3,016	-
253.1	Pituitary hyperfunction	16/5,791	62.09 (8.23-468.41)	5/4,935	-	4/4,062	7.29 (1.33-39.94)	5/3,000	6 (1.43-25.19)
245	Thyroiditis	9/5,809	11.21 (3.03-41.49)	2/4,950	1.9 (0.35-10.41)	4/4,079	4.66 (1.03-21.05)	5/3,019	3.89 (1.12-13.44)
226	Benign neoplasm of thyroid glands	11/5,811	44 (5.68-340.82)	4/4,951	8 (1.47-43.68)	1/4,076	1 (0.11-8.95)	0/3,015	-
**Musculoskeletal And Joint Diseases**									
743.11	Osteoporosis NOS	66/5,426	2.05 (1.51-2.78)	66/4,638	1.68 (1.25-2.25)	65/3,852	1.03 (0.78-1.35)	114/2,859	1.12 (0.9-1.39)
760	Back pain	48/5,526	1.64 (1.16-2.31)	65/4,767	1.88 (1.39-2.54)	64/3,954	1.18 (0.89-1.57)	145/2,945	1.27 (1.05-1.54)
721	Spondylosis and allied disorders	25/5,680	2.26 (1.38-3.71)	31/4,858	1.44 (0.95-2.18)	32/4,005	1.38 (0.92-2.07)	59/2,966	1.37 (1.01-1.85)
**Psychiatric And Behavioral Diseases**									
300.1	Anxiety disorder	62/5,550	1.91 (1.4-2.61)	68/4,760	1.61 (1.21-2.15)	76/3,941	1.22 (0.94-1.58)	157/2,934	1.24 (1.03-1.49)
290.2	Delirium due to conditions classified elsewhere	40/5,769	4 (2.55-6.26)	22/4,950	1.73 (1.04-2.87)	32/4,090	1.7 (1.11-2.62)	60/3,026	1.3 (0.97-1.75)
**Respiratory Diseases**									

496	Chronic airway obstruction	50/5,573	2.16 (1.52-3.08)	45/4,790	1.93 (1.34-2.76)	78/3,970	1.6 (1.23-2.1)	118/2,950	1.05 (0.85-1.29)
507	Pleurisy; pleural effusion	71/5,673	3.46 (2.5-4.8)	51/4,869	2.09 (1.47-2.96)	70/4,023	1.75 (1.31-2.33)	86/2,982	0.92 (0.73-1.17)
519.8	Other diseases of respiratory system, NEC	46/5,591	2.54 (1.74-3.71)	56/4,805	2.37 (1.69-3.33)	52/3,955	1.25 (0.91-1.72)	82/2,943	1.13 (0.88-1.45)
508	Pulmonary collapse; interstitial and compensatory emphysema	44/5,738	4.27 (2.75-6.62)	33/4,918	2.23 (1.44-3.45)	32/4,056	1.36 (0.9-2.05)	60/3,011	1.16 (0.86-1.55)
509.1	Respiratory failure	44/5,757	3.84 (2.51-5.87)	21/4,936	1.63 (0.98-2.73)	37/4,081	1.44 (0.98-2.12)	66/3,018	1.33 (1-1.77)
496.2	Chronic bronchitis	24/5,695	1.9 (1.16-3.14)	18/4,893	1.47 (0.85-2.54)	46/4,046	2 (1.39-2.87)	72/2,992	1.39 (1.06-1.83)
327.3	Sleep apnea	15/5,726	1.87 (1-3.47)	25/4,892	2.2 (1.34-3.61)	30/4,034	1.64 (1.07-2.52)	59/2,984	1.49 (1.1-2.02)
479	Other upper respiratory disease	17/5,734	2.89 (1.53-5.46)	16/4,893	2.16 (1.17-4)	17/4,025	1.75 (0.98-3.13)	19/2,978	1.38 (0.81-2.33)
501	Pneumonitis due to inhalation of food or vomitus	21/5,812	3.94 (2.13-7.27)	9/4,965	2.28 (1-5.23)	11/4,093	1.31 (0.64-2.71)	26/3,029	1.51 (0.95-2.43)
**Skin Diseases**									
707.1	Decubitus ulcer	24/5,788	3.94 (2.22-7)	22/4,951	2.82 (1.61-4.94)	31/4,088	2.16 (1.37-3.4)	40/3,025	1.23 (0.85-1.76)
681.7	Cellulitis and abscess of trunk	5/5,819	3 (0.91-9.86)	4/4,967	2.44 (0.69-8.68)	7/4,091	3.73 (1.3-10.69)	14/3,027	4.09 (1.92-8.7)

HT = Hyperthyroidism; HR = Hazard ratio; CI = Confidence interval.

Table S5. Results of landmark analyses to investigate the time-varying effect of prior diagnosed hyperthyroidism on causes of death.

Cause of death	Within 2 years since hyperthyroidism		2 to 5 year since hyperthyroidism		5 to10 year since hyperthyroidism		Beyond 10 years since hyperthyroidism	
	Cases/Participants (HT population)	HR (95% CI)	Cases/Participants (HT population)	HR (95% CI)	Cases/Participants (HT population)	HR (95% CI)	Cases/Participants (HT population)	HR (95% CI)
All-cause mortality	268/5,828	5.62 (4.65-6.8)	138/4,976	2.24 (1.81-2.76)	151/4,099	1.63 (1.34-1.97)	209/3,031	1.01 (0.87-1.18)
Malignant neoplasms death	125/5,832	5.4 (4.1-7.12)	54/4,976	1.74 (1.27-2.4)	56/4,099	1.3 (0.96-1.75)	84/3,031	0.94 (0.74-1.19)
Cardiovascular disease death	41/5,832	3.43 (2.2-5.36)	28/4,976	2.65 (1.64-4.27)	39/4,099	2.31 (1.56-3.43)	54/3,031	1.63 (1.19-2.23)
Respiratory system disease death	28/5,832	7.04 (3.56-13.9)	21/4,976	3.76 (2.08-6.79)	11/4,099	1.29 (0.65-2.56)	19/3,031	1.14 (0.69-1.9)
Neural system disease death	15/5,832	2.73 (1.36-5.49)	12/4,976	3.1 (1.47-6.51)	6/4,099	0.84 (0.34-2.06)	10/3,031	0.8 (0.41-1.55)
Digestive system disease death	12/5,832	4.49 (1.89-10.71)	6/4,976	1.79 (0.66-4.87)	8/4,099	2.21 (0.9-5.47)	14/3,031	1.95 (1.03-3.71)
Mental disorder death	12/5,832	10.79 (3.68-31.66)	4/4,976	2.75 (0.72-10.43)	5/4,099	1.8 (0.67-4.84)	5/3,031	0.81 (0.3-2.18)

HT = Hyperthyroidism; HR = Hazard ratio; CI = Confidence interval.

All p-value reported has been adjusted using Bonferroni correction.

Disease Trajectory Analysis

Figure S5. Research flowchart for identifying disease pairs in the disease trajectory following hyperthyroidism.

OR = Odds ratio.

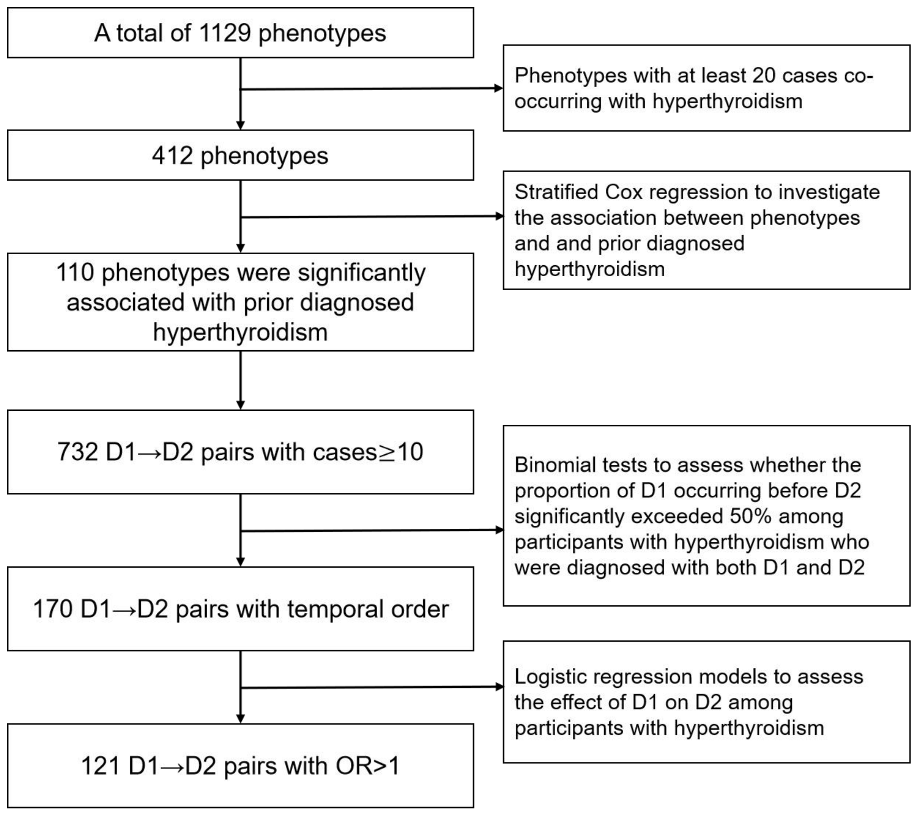

Table S6. Prevalence and odds ratios with 95% confidence intervals for the disease pairs in disease trajectories following hyperthyroidism.

D1	D1 Phenotype	D2	D2 Phenotype	N_D1_D2	N_both	OR (95% CI)	P-value
244	Hypothyroid conditions	250.2	Type 2 diabetes	104	159	3 (2.32-3.88)	9.71E-15
244	Hypothyroid conditions	261.4	Vitamin D deficiency	43	52	2.7 (1.8-4.06)	3.05E-04
244	Hypothyroid conditions	276.1	Electrolyte imbalance	91	106	1.85 (1.41-2.43)	1.42E-03
244	Hypothyroid conditions	276.5	Hypovolemia	66	76	2.82 (1.97-4.04)	2.71E-06
244	Hypothyroid conditions	280.1	Iron deficiency anemias	107	137	2.73 (2.08-3.57)	5.67E-11
244	Hypothyroid conditions	285	Other anemias	93	131	1.9 (1.49-2.44)	6.03E-05
244	Hypothyroid conditions	300.1	Anxiety disorder	110	130	2.73 (2.09-3.57)	3.85E-11
244	Hypothyroid conditions	395.1	Nonrheumatic mitral valve disorders	35	47	2.36 (1.54-3.62)	1.41E-02
244	Hypothyroid conditions	411.4	Coronary atherosclerosis	79	109	2.55 (1.9-3.43)	8.85E-08
244	Hypothyroid conditions	411.8	Other chronic ischemic heart disease, unspecified	95	123	2.25 (1.71-2.97)	1.08E-06
244	Hypothyroid conditions	414	Other forms of chronic heart disease	39	54	2.18 (1.45-3.27)	3.10E-02
244	Hypothyroid conditions	416	Cardiomegaly	48	65	2.19 (1.51-3.17)	5.68E-03
244	Hypothyroid conditions	427.2	Atrial fibrillation and flutter	81	112	2 (1.53-2.62)	8.35E-05
244	Hypothyroid conditions	428	Heart failure	67	98	1.89 (1.4-2.55)	4.55E-03
244	Hypothyroid conditions	433.2	Occlusion of cerebral arteries	40	53	2.79 (1.83-4.25)	3.07E-04
244	Hypothyroid conditions	458.9	Hypotension NOS	70	79	2.52 (1.78-3.56)	2.80E-05
244	Hypothyroid conditions	480.1	Bacterial pneumonia	82	109	1.78 (1.35-2.35)	8.78E-03
244	Hypothyroid conditions	496	Chronic airway obstruction	80	96	2.23 (1.65-3.03)	4.11E-05
244	Hypothyroid conditions	507	Pleurisy; pleural effusion	66	92	2.08 (1.52-2.84)	7.95E-04
244	Hypothyroid conditions	519.8	Other diseases of respiratory system, NEC	56	78	2.19 (1.57-3.05)	6.22E-04
244	Hypothyroid conditions	530.1	Esophagitis, GERD and related diseases	158	237	2.28 (1.87-2.78)	1.13E-13
244	Hypothyroid conditions	535.8	Other specified gastritis	61	84	2.34 (1.69-3.23)	4.45E-05
244	Hypothyroid conditions	550.2	Diaphragmatic hernia	121	181	1.98 (1.58-2.47)	3.13E-07
244	Hypothyroid conditions	563	Constipation	118	139	2.3 (1.78-2.98)	4.19E-08
244	Hypothyroid conditions	571.5	Other chronic nonalcoholic liver disease	47	55	2.69 (1.78-4.06)	4.70E-04
244	Hypothyroid conditions	585.1	Acute renal failure	125	142	2.02 (1.56-2.6)	1.16E-05
244	Hypothyroid conditions	585.3	Chronic renal failure [CKD]	114	134	2.43 (1.85-3.19)	2.26E-08
244	Hypothyroid conditions	591	Urinary tract infection	111	144	2.22 (1.72-2.87)	1.43E-07
244	Hypothyroid conditions	707.1	Decubitus ulcer	39	41	2.63 (1.6-4.3)	2.12E-02
244	Hypothyroid conditions	721	Spondylosis and allied disorders	45	54	2.49 (1.64-3.79)	3.50E-03
244	Hypothyroid conditions	743.11	Osteoporosis NOS	77	102	1.99 (1.5-2.64)	3.80E-04
244	Hypothyroid conditions	760	Back pain	62	91	1.88 (1.41-2.5)	2.95E-03
244	Hypothyroid conditions	8	Intestinal infection	99	122	2.37 (1.81-3.12)	9.32E-08
250.2	Type 2 diabetes	251.1	Hypoglycemia	21	23	14.21 (7.17-28.19)	5.10E-12
250.2	Type 2 diabetes	276.1	Electrolyte imbalance	41	55	2.76 (1.97-3.85)	4.80E-07
250.2	Type 2 diabetes	280.1	Iron deficiency anemias	45	54	3.11 (2.25-4.29)	9.36E-10
250.2	Type 2 diabetes	426.3	Bundle branch block	22	29	2.42 (1.53-3.83)	2.72E-02

250.2	Type 2 diabetes	428	Heart failure	40	55	2.33 (1.65-3.28)	2.20E-04
250.2	Type 2 diabetes	480	Pneumonia	30	43	2.25 (1.54-3.29)	4.29E-03
250.2	Type 2 diabetes	480.1	Bacterial pneumonia	45	55	2.72 (1.93-3.82)	1.52E-06
250.2	Type 2 diabetes	509.1	Respiratory failure	25	30	2.57 (1.64-4.03)	6.68E-03
250.2	Type 2 diabetes	535.8	Other specified gastritis	26	36	2.69 (1.83-3.96)	9.02E-05
250.2	Type 2 diabetes	571.5	Other chronic nonalcoholic liver disease	28	35	5.29 (3.43-8.16)	7.95E-12
250.2	Type 2 diabetes	585.1	Acute renal failure	59	74	2.84 (2.11-3.83)	1.29E-09
250.2	Type 2 diabetes	585.3	Chronic renal failure [CKD]	57	77	3.86 (2.88-5.19)	3.89E-17
250.2	Type 2 diabetes	8	Intestinal infection	42	57	2.69 (1.93-3.73)	6.84E-07
250.2	Type 2 diabetes	994.2	Sepsis	29	34	2.55 (1.69-3.84)	1.26E-03
280.1	Iron deficiency anemias	276.1	Electrolyte imbalance	35	49	2.85 (2.06-3.94)	3.95E-08
280.1	Iron deficiency anemias	276.5	Hypovolemia	25	34	2.63 (1.78-3.88)	2.09E-04
285	Other anemias	458.9	Hypotension NOS	33	47	4.35 (3.09-6.11)	4.59E-15
285	Other anemias	480	Pneumonia	40	48	2.81 (2-3.96)	4.94E-07
285	Other anemias	707.1	Decubitus ulcer	23	29	5.17 (3.28-8.15)	2.78E-10
300.1	Anxiety disorder	276.5	Hypovolemia	21	27	2.24 (1.48-3.39)	2.13E-02
300.1	Anxiety disorder	290.2	Delirium due to conditions classified elsewhere	22	24	3.43 (2.17-5.43)	2.51E-05
300.1	Anxiety disorder	509.1	Respiratory failure	20	24	2.61 (1.65-4.12)	6.32E-03
300.1	Anxiety disorder	585.1	Acute renal failure	35	48	2.01 (1.45-2.8)	5.63E-03
401.2	Hypertensive heart and/or renal disease	585.3	Chronic renal failure [CKD]	22	27	12.71 (6.85-23.61)	1.36E-13
411.2	Myocardial infarction	411.8	Other chronic ischemic heart disease, unspecified	53	81	22.86 (16.08-32.5)	6.79E-66
411.2	Myocardial infarction	480	Pneumonia	23	30	3.29 (2.18-4.96)	2.63E-06
411.4	Coronary atherosclerosis	276.1	Electrolyte imbalance	39	55	3.37 (2.41-4.71)	2.15E-10
411.4	Coronary atherosclerosis	276.6	Fluid overload	20	22	3.58 (2.07-6.19)	8.50E-04
411.4	Coronary atherosclerosis	285	Other anemias	33	47	2.38 (1.69-3.35)	1.24E-04
411.4	Coronary atherosclerosis	411.8	Other chronic ischemic heart disease, unspecified	87	112	25.31 (18.47-34.68)	1.06E-87
411.4	Coronary atherosclerosis	458.9	Hypotension NOS	29	32	3.7 (2.47-5.54)	3.96E-08
411.4	Coronary atherosclerosis	507	Pleurisy; pleural effusion	31	40	3.16 (2.19-4.56)	1.39E-07
411.4	Coronary atherosclerosis	585.1	Acute renal failure	49	64	3.09 (2.26-4.22)	3.27E-10
411.4	Coronary atherosclerosis	585.3	Chronic renal failure [CKD]	40	58	3.36 (2.42-4.66)	6.62E-11
411.8	Other chronic ischemic heart disease, unspecified	251.1	Hypoglycemia	22	29	7.46 (4.31-12.9)	1.11E-10
411.8	Other chronic ischemic heart disease, unspecified	416	Cardiomegaly	26	35	3.81 (2.57-5.67)	5.66E-09
411.8	Other chronic ischemic heart disease, unspecified	480	Pneumonia	33	48	3.11 (2.18-4.44)	6.89E-08
411.8	Other chronic ischemic heart disease, unspecified	585.1	Acute renal failure	46	69	2.73 (2.02-3.68)	9.49E-09
411.8	Other chronic ischemic heart disease, unspecified	585.3	Chronic renal failure [CKD]	48	69	4.1 (3.04-5.52)	3.50E-18
426.3	Bundle branch block	480	Pneumonia	25	30	4.37 (2.89-6.61)	4.32E-10
427.2	Atrial fibrillation and flutter	276.5	Hypovolemia	23	31	2.94 (1.97-4.39)	2.05E-05
427.2	Atrial fibrillation and flutter	585.3	Chronic renal failure [CKD]	50	75	4.36 (3.25-5.86)	2.19E-20
428	Heart failure	480	Pneumonia	54	72	7.77 (5.63-10.71)	1.40E-33

428	Heart failure	707.1	Decubitus ulcer	21	27	5.5 (3.37-8.96)	1.34E-09
433.2	Occlusion of cerebral arteries	433.8	Late effects of cerebrovascular disease	33	34	147.08 (67.32-321.36)	1.03E-33
433.2	Occlusion of cerebral arteries	480	Pneumonia	24	29	4.74 (3.03-7.4)	1.34E-09
433.2	Occlusion of cerebral arteries	563	Constipation	22	29	3.07 (2.02-4.69)	3.14E-05
458.9	Hypotension NOS	276.5	Hypovolemia	25	34	6.86 (4.69-10.03)	6.04E-21
480.1	Bacterial pneumonia	480	Pneumonia	61	93	11.03 (8.09-15.04)	6.79E-50
480.1	Bacterial pneumonia	509.1	Respiratory failure	36	50	16.07 (11.11-23.25)	6.02E-47
496	Chronic airway obstruction	276.1	Electrolyte imbalance	30	39	2.23 (1.54-3.23)	3.93E-03
496	Chronic airway obstruction	585.1	Acute renal failure	44	62	2.54 (1.83-3.54)	5.84E-06
496	Chronic airway obstruction	585.3	Chronic renal failure [CKD]	39	58	2.94 (2.11-4.09)	3.40E-08
507	Pleurisy; pleural effusion	480	Pneumonia	41	60	7.01 (5.05-9.74)	4.85E-29
507	Pleurisy; pleural effusion	707.1	Decubitus ulcer	21	28	7.2 (4.55-11.38)	4.95E-15
530.1	Esophagitis, GERD and related diseases	280.1	Iron deficiency anemias	50	72	2.44 (1.84-3.22)	6.74E-08
530.1	Esophagitis, GERD and related diseases	300.1	Anxiety disorder	63	84	3.24 (2.47-4.25)	2.55E-15
530.1	Esophagitis, GERD and related diseases	41.4	E. coli	30	36	2.76 (1.86-4.09)	7.83E-05
530.1	Esophagitis, GERD and related diseases	458.1	Orthostatic hypotension	21	28	2.76 (1.73-4.4)	3.19E-03
530.1	Esophagitis, GERD and related diseases	585.1	Acute renal failure	49	72	1.72 (1.3-2.28)	2.93E-02
530.1	Esophagitis, GERD and related diseases	591	Urinary tract infection	60	84	2.64 (2.03-3.44)	6.85E-11
530.1	Esophagitis, GERD and related diseases	79	Viral infection	20	25	2.72 (1.71-4.34)	4.11E-03
530.1	Esophagitis, GERD and related diseases	8	Intestinal infection	64	81	2.57 (1.95-3.39)	4.12E-09
535	Gastritis and duodenitis	300.1	Anxiety disorder	37	45	2.47 (1.74-3.5)	6.21E-05
535	Gastritis and duodenitis	585.1	Acute renal failure	33	46	2.18 (1.54-3.08)	1.85E-03
535	Gastritis and duodenitis	585.3	Chronic renal failure [CKD]	39	53	2.8 (2-3.91)	2.63E-07
550.2	Diaphragmatic hernia	275.3	Disorders of magnesium metabolism	25	28	4.38 (2.75-6.98)	8.46E-08
550.2	Diaphragmatic hernia	585.1	Acute renal failure	47	67	1.99 (1.49-2.66)	6.10E-04
558	Noninfectious gastroenteritis	458.9	Hypotension NOS	22	27	4.66 (3.01-7.21)	8.37E-10
558	Noninfectious gastroenteritis	8	Intestinal infection	30	42	4.13 (2.85-5.99)	1.27E-11
585.1	Acute renal failure	276.6	Fluid overload	29	39	17.53 (10.41-29.52)	8.41E-25
585.1	Acute renal failure	480	Pneumonia	52	77	6.72 (4.99-9.05)	9.82E-34
585.1	Acute renal failure	707.1	Decubitus ulcer	35	43	14.25 (9.14-22.24)	1.87E-29
585.3	Chronic renal failure [CKD]	276.1	Electrolyte imbalance	50	77	4.52 (3.4-6.02)	7.40E-23
585.3	Chronic renal failure [CKD]	276.5	Hypovolemia	31	41	3.51 (2.46-5)	5.99E-10
585.3	Chronic renal failure [CKD]	480	Pneumonia	42	61	3.83 (2.78-5.27)	3.49E-14
585.3	Chronic renal failure [CKD]	707.1	Decubitus ulcer	25	30	4.24 (2.68-6.73)	1.29E-07
591	Urinary tract infection	41.4	E. coli	33	38	120.15 (71-203.33)	5.73E-69
591	Urinary tract infection	416	Cardiomegaly	25	33	2.14 (1.42-3.22)	4.70E-02
591	Urinary tract infection	480	Pneumonia	40	56	3.28 (2.38-4.53)	7.91E-11
591	Urinary tract infection	507	Pleurisy; pleural effusion	35	51	2.99 (2.15-4.16)	1.20E-08
591	Urinary tract infection	707.1	Decubitus ulcer	35	38	9.39 (6.01-14.68)	1.49E-20

591	Urinary tract infection	994.2	Sepsis	34	49	8.34 (6.02-11.56)	5.19E-35
743.11	Osteoporosis NOS	585.1	Acute renal failure	36	51	3.21 (2.3-4.46)	8.34E-10
743.11	Osteoporosis NOS	8	Intestinal infection	31	44	2.72 (1.92-3.86)	3.36E-06
785	Abdominal pain	300.1	Anxiety disorder	21	26	3.44 (2.21-5.37)	8.19E-06
79	Viral infection	480	Pneumonia	25	33	8.34 (5.26-13.21)	3.08E-17
8	Intestinal infection	480	Pneumonia	38	53	4.02 (2.87-5.63)	9.27E-14

All p-value reported has been adjusted using Bonferroni correction.

OR = Odds ratio; CI = Confidence interval.

Table S7. Mediation effect of D2 within a length-3 disease trajectory D1→D2→D3 in disease trajectories following hyperthyroidism.

D1	D2	D3	Direct effect		Indirect effect		Total effect		Mediated proportion
			Effect	P-value	Effect	P-value	Effect	P-value	
242	250.2	280.1	0.02	<0.001	0.0011	<0.001	0.021	<0.001	5.1%
242	250.2	426.3	0.011	<0.001	0.00045	<0.001	0.012	<0.001	3.9%
242	250.2	428	0.021	<0.001	0.001	<0.001	0.022	<0.001	4.6%
242	250.2	480.1	0.018	<0.001	0.00097	<0.001	0.019	<0.001	5.1%
242	250.2	585.1	0.028	<0.001	0.0015	<0.001	0.03	<0.001	5.0%
242	250.2	585.3	0.022	<0.001	0.0015	<0.001	0.024	<0.001	6.4%
242	250.2	8	0.021	<0.001	0.00083	<0.001	0.022	<0.001	3.8%
242	401.2	585.3	0.021	<0.001	0.0025	<0.001	0.024	<0.001	10.6%
242	411.2	411.8	0.016	<0.001	0.0026	0.016	0.018	<0.001	14.5%
242	411.4	285	0.027	<0.001	0.00095	0.004	0.028	<0.001	3.4%
242	411.4	411.8	0.014	<0.001	0.004	<0.001	0.018	<0.001	21.7%
242	411.4	507	0.014	<0.001	0.00097	<0.001	0.015	<0.001	6.3%
242	427.2	585.3	0.023	<0.001	0.0011	0.028	0.024	<0.001	4.6%
242	496	585.1	0.028	<0.001	0.0015	<0.001	0.03	<0.001	5.0%
242	496	585.3	0.023	<0.001	0.0013	<0.001	0.024	<0.001	5.5%
242	530.1	280.1	0.02	<0.001	0.00098	0.004	0.021	<0.001	4.6%
242	530.1	300.1	0.012	<0.001	0.0011	0.004	0.013	<0.001	8.4%
242	530.1	591	0.027	<0.001	0.00082	0.004	0.028	<0.001	3.0%
242	530.1	79	0.0058	<0.001	0.00018	0.004	0.006	<0.001	3.1%
242	530.1	8	0.021	<0.001	0.00084	<0.001	0.022	<0.001	3.8%
242	535	300.1	0.012	<0.001	0.0009	<0.001	0.013	<0.001	7.0%
242	535	585.3	0.023	<0.001	0.00092	<0.001	0.024	<0.001	3.8%
242	558	458.9	0.012	<0.001	0.00067	<0.001	0.013	<0.001	5.1%
242	558	8	0.021	<0.001	0.0015	<0.001	0.022	<0.001	6.9%
242	743.11	585.1	0.029	<0.001	0.0012	<0.001	0.03	<0.001	4.0%
242	743.11	8	0.021	<0.001	0.00093	<0.001	0.022	<0.001	4.2%
242	785	300.1	0.012	<0.001	0.00078	<0.001	0.013	<0.001	6.0%
242	250.2	251.1	0.0069	<0.001	0.00046	<0.001	0.0073	<0.001	6.2%
242	250.2	535.8	0.011	<0.001	0.00054	<0.001	0.012	<0.001	4.5%
242	250.2	571.5	0.009	<0.001	0.00073	0.004	0.0097	<0.001	7.5%
242	250.2	994.2	0.016	<0.001	0.0006	<0.001	0.017	<0.001	3.6%
242	433.2	480	0.017	<0.001	0.00079	<0.001	0.018	<0.001	4.4%
242	433.2	433.8	0.0039	0.004	0.0015	<0.001	0.0054	<0.001	27.7%
242	433.2	563	0.012	<0.001	0.0011	<0.001	0.013	<0.001	8.2%
242	530.1	458.1	0.0057	<0.001	0.00029	<0.001	0.006	<0.001	4.9%
250.2	280.1	276.1	0.067	<0.001	0.0096	<0.001	0.077	<0.001	12.5%

250.2	280.1	276.5	0.042	<0.001	0.0054	<0.001	0.047	<0.001	11.5%
250.2	426.3	480	0.046	<0.001	0.0047	<0.001	0.051	<0.001	9.2%
250.2	428	480	0.036	<0.001	0.015	<0.001	0.051	<0.001	29.0%
250.2	428	707.1	0.02	<0.001	0.0069	<0.001	0.026	<0.001	26.1%
250.2	480.1	480	0.032	<0.001	0.019	<0.001	0.051	<0.001	37.6%
250.2	480.1	509.1	0.019	<0.001	0.016	<0.001	0.035	<0.001	47.1%
250.2	585.1	480	0.029	<0.001	0.022	<0.001	0.051	<0.001	43.7%
250.2	585.1	707.1	0.013	<0.001	0.014	<0.001	0.026	<0.001	51.2%
250.2	585.1	276.6	0.007	0.06	0.01	<0.001	0.017	<0.001	58.8%
250.2	585.3	276.1	0.058	<0.001	0.019	<0.001	0.077	<0.001	24.6%
250.2	585.3	276.5	0.035	<0.001	0.012	<0.001	0.047	<0.001	26.1%
250.2	585.3	480	0.04	<0.001	0.012	<0.001	0.051	<0.001	22.6%
250.2	585.3	707.1	0.019	<0.001	0.0071	<0.001	0.026	<0.001	26.9%
250.2	8	480	0.044	<0.001	0.0073	<0.001	0.051	<0.001	14.4%
401.2	585.3	276.1	0.18	<0.001	0.071	<0.001	0.25	<0.001	28.6%
401.2	585.3	276.5	0.059	0.02	0.048	<0.001	0.11	<0.001	44.9%
401.2	585.3	480	0.11	<0.001	0.044	<0.001	0.16	<0.001	28.0%
401.2	585.3	707.1	0.056	0.024	0.027	<0.001	0.083	<0.001	32.6%
411.2	411.8	585.1	0.11	<0.001	0.042	<0.001	0.15	<0.001	28.1%
411.2	411.8	585.3	0.083	<0.001	0.052	<0.001	0.13	<0.001	38.2%
411.2	411.8	251.1	0.021	0.004	0.012	<0.001	0.033	<0.001	36.9%
411.2	411.8	416	0.084	<0.001	0.028	<0.001	0.11	<0.001	25.3%
411.4	285	458.9	0.066	<0.001	0.01	<0.001	0.076	<0.001	13.2%
411.4	285	480	0.049	<0.001	0.01	<0.001	0.059	<0.001	17.5%
411.4	285	707.1	0.014	0.012	0.0065	<0.001	0.021	<0.001	31.8%
411.4	411.8	585.1	0.069	<0.001	0.043	<0.001	0.11	<0.001	38.7%
411.4	411.8	585.3	0.067	<0.001	0.05	<0.001	0.12	<0.001	42.9%
411.4	411.8	251.1	0.008	0.132	0.013	<0.001	0.021	<0.001	62.8%
411.4	411.8	416	0.087	<0.001	0.024	<0.001	0.11	<0.001	21.8%
411.4	507	480	0.04	<0.001	0.019	<0.001	0.059	<0.001	32.2%
411.4	507	707.1	0.01	0.032	0.01	<0.001	0.021	<0.001	48.9%
427.2	585.3	276.1	0.12	<0.001	0.02	<0.001	0.14	<0.001	14.6%
427.2	585.3	276.5	0.045	<0.001	0.014	<0.001	0.059	<0.001	23.9%
427.2	585.3	480	0.087	<0.001	0.012	<0.001	0.1	<0.001	12.2%
427.2	585.3	707.1	0.04	<0.001	0.0077	<0.001	0.048	<0.001	16.1%
496	585.1	480	0.1	<0.001	0.023	<0.001	0.13	<0.001	18.1%
496	585.1	707.1	0.022	<0.001	0.014	<0.001	0.037	<0.001	39.5%
496	585.1	276.6	0.016	0.008	0.011	<0.001	0.027	<0.001	39.9%
496	585.3	276.1	0.097	<0.001	0.017	<0.001	0.11	<0.001	15.0%

496	585.3	276.5	0.04	<0.001	0.011	<0.001	0.052	<0.001	22.1%
496	585.3	480	0.12	<0.001	0.0099	<0.001	0.13	<0.001	7.8%
496	585.3	707.1	0.03	<0.001	0.0065	<0.001	0.037	<0.001	17.7%
530.1	280.1	276.1	0.049	<0.001	0.0075	<0.001	0.057	<0.001	13.2%
530.1	280.1	276.5	0.029	<0.001	0.0043	<0.001	0.034	<0.001	12.8%
530.1	300.1	585.1	0.054	<0.001	0.0046	<0.001	0.059	<0.001	7.8%
530.1	300.1	276.5	0.031	<0.001	0.0029	<0.001	0.034	<0.001	8.8%
530.1	300.1	290.2	0.014	<0.001	0.0031	<0.001	0.017	<0.001	17.8%
530.1	300.1	509.1	0.013	<0.001	0.0025	<0.001	0.015	<0.001	16.2%
530.1	591	507	0.034	<0.001	0.0056	<0.001	0.039	<0.001	14.3%
530.1	591	994.2	0.023	<0.001	0.008	<0.001	0.031	<0.001	26.1%
530.1	591	416	0.022	<0.001	0.0029	<0.001	0.025	<0.001	11.7%
530.1	591	41.4	0.013	<0.001	0.016	<0.001	0.028	<0.001	54.5%
530.1	79	480	0.022	<0.001	0.0026	<0.001	0.025	<0.001	10.5%
530.1	8	480	0.018	<0.001	0.0065	<0.001	0.025	<0.001	26.5%
535	300.1	585.1	0.046	<0.001	0.005	<0.001	0.051	<0.001	9.7%
535	300.1	276.5	0.024	<0.001	0.0032	<0.001	0.027	<0.001	11.7%
535	300.1	290.2	0.014	0.004	0.0032	<0.001	0.017	<0.001	18.7%
535	300.1	509.1	0.011	0.016	0.0026	<0.001	0.014	0.008	18.4%
535	585.3	276.1	0.051	<0.001	0.012	<0.001	0.063	<0.001	19.5%
535	585.3	276.5	0.019	<0.001	0.0081	<0.001	0.027	<0.001	30.1%
535	585.3	480	0.026	<0.001	0.0076	<0.001	0.033	<0.001	22.9%
535	585.3	707.1	0.012	<0.001	0.0047	<0.001	0.017	<0.001	27.8%
558	458.9	276.5	0.056	<0.001	0.0087	<0.001	0.064	<0.001	13.5%
558	8	480	0.03	<0.001	0.014	<0.001	0.044	<0.001	31.5%
743.11	585.1	480	0.048	<0.001	0.017	<0.001	0.066	<0.001	26.5%
743.11	585.1	707.1	0.03	<0.001	0.01	<0.001	0.04	<0.001	26.0%
743.11	585.1	276.6	0.015	<0.001	0.0077	<0.001	0.022	<0.001	34.6%
743.11	8	480	0.058	<0.001	0.008	<0.001	0.066	<0.001	12.1%
785	300.1	585.1	0.011	0.244	0.0068	<0.001	0.018	0.112	37.9%
785	300.1	276.5	0.012	0.148	0.0043	<0.001	0.016	0.032	26.4%
785	300.1	290.2	0.0093	0.156	0.0043	<0.001	0.014	0.024	31.4%
785	300.1	509.1	0.0024	0.764	0.0035	<0.001	0.0059	0.392	58.7%
411.8	585.3	276.1	0.097	<0.001	0.022	<0.001	0.12	<0.001	18.5%
411.8	585.3	276.5	0.053	<0.001	0.015	<0.001	0.068	<0.001	21.4%
285	458.9	276.5	0.074	<0.001	0.016	<0.001	0.09	<0.001	17.3%
411.8	585.1	276.6	0.023	<0.001	0.011	<0.001	0.033	<0.001	32.4%
300.1	585.1	276.6	0.016	<0.001	0.0056	<0.001	0.022	<0.001	26.1%
411.8	585.1	480	0.064	<0.001	0.024	<0.001	0.088	<0.001	27.6%

411.8	585.3	480	0.075	<0.001	0.013	<0.001	0.088	<0.001	15.0%
300.1	585.1	480	0.028	<0.001	0.013	<0.001	0.041	<0.001	30.9%
591	507	480	0.1	<0.001	0.019	<0.001	0.12	<0.001	15.7%
411.8	585.1	707.1	0.023	<0.001	0.015	<0.001	0.038	<0.001	39.3%
411.8	585.3	707.1	0.03	<0.001	0.0084	<0.001	0.038	<0.001	22.1%
300.1	585.1	707.1	0.013	0.004	0.0077	<0.001	0.021	<0.001	37.2%
591	507	707.1	0.078	<0.001	0.0093	<0.001	0.087	<0.001	10.7%

CI = Confidence interval.

All p-value reported has been adjusted using Bonferroni correction.

Figure S6. An overall view of disease trajectory following hyperthyroidism.

In the Sankey diagram, each node represents a phenotype. The color of the node indicates disease categories by physiological system. Links between nodes represent associations between disease pairs, with the width of the links reflecting the frequency of these disease pairs among participants with hyperthyroidism.

Hypothyroidism is usually a temporary condition shortly after treatment for hyperthyroidism, and most patients gradually return to normal, so it was not included in the disease trajectory.

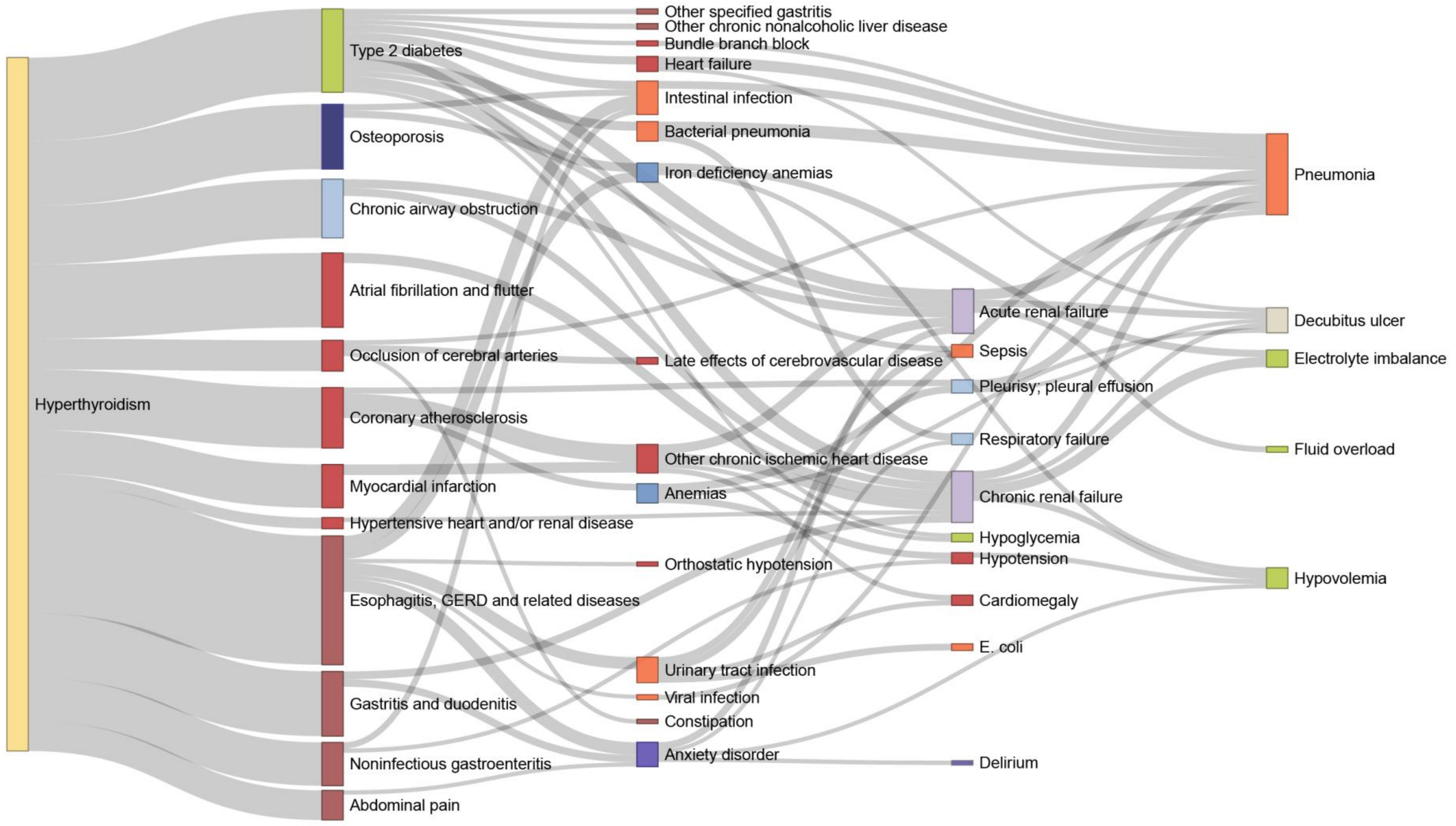

Figure S7. Research flowchart for identifying disease pairs in the disease trajectory leading to death among participants diagnosed with hyperthyroidism.

OR = Odds ratio.

CVDD = Cardiovascular disease death; RSDD = Respiratory system disease death; MND = Malignant neoplasms death; DSDD = Digestive system disease death.

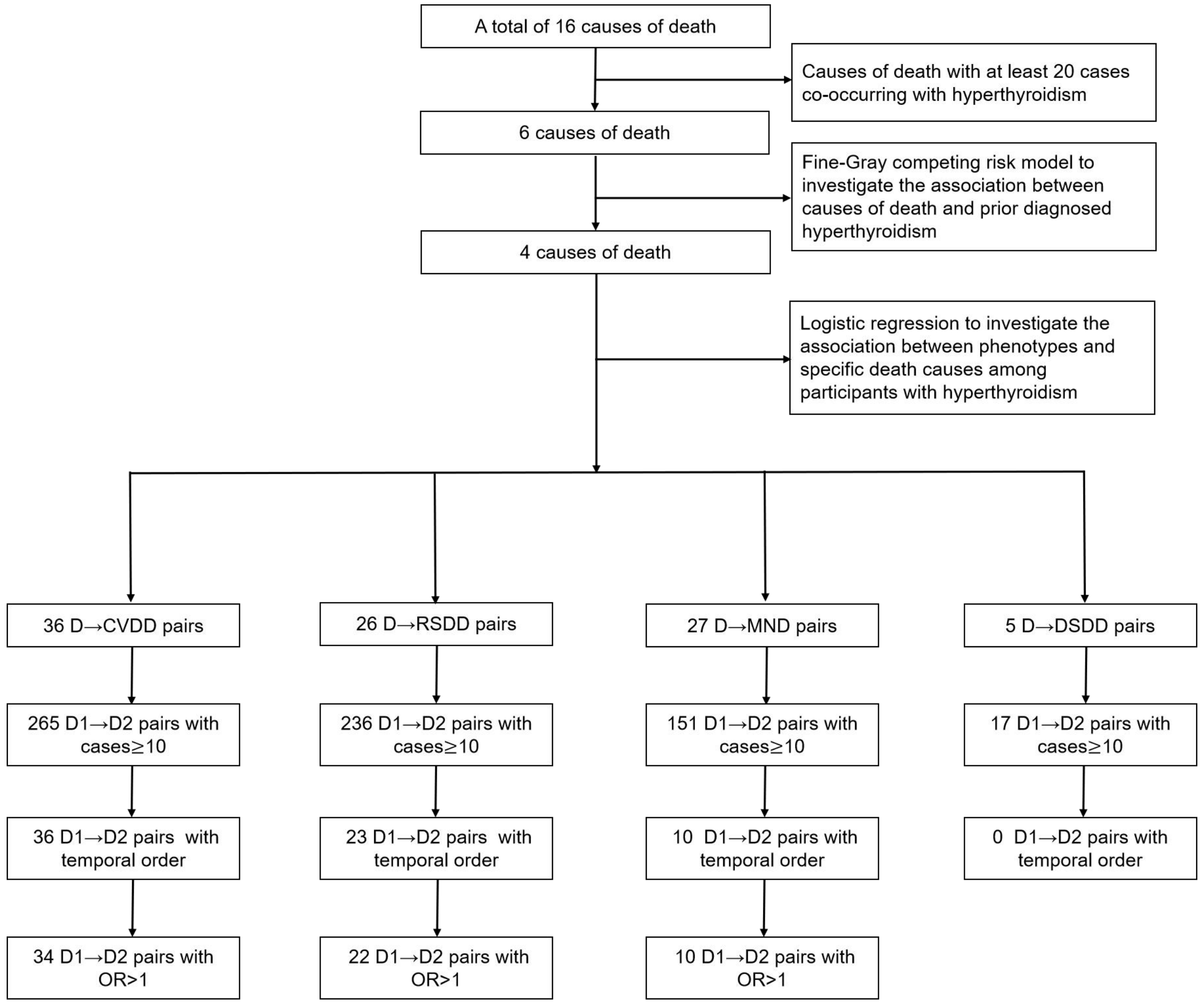

Table S8. Prevalence and odds ratios with 95% confidence intervals for the disease pairs in disease trajectories leading to death.

D1	D1 Phenotype	D2	D2 Phenotype	N_D1_D2	N_both	OR (95% CI)	P-value
**CVDD**							
251.1	Hypoglycemia	CVDD		20	20	6.38 (3.51-11.59)	1.22E-09
276.1	Electrolyte imbalance	CVDD		37	37	3.74 (2.45-5.71)	9.00E-10
276.4	Acid-base balance disorder	CVDD		11	11	3.11 (1.55-6.26)	1.45E-03
276.6	Fluid overload	CVDD		17	17	4.53 (2.49-8.23)	7.14E-07
280.1	Iron deficiency anemias	CVDD		21	21	1.95 (1.18-3.22)	8.83E-03
290.2	Delirium due to conditions classified elsewhere	CVDD		15	15	1.97 (1.07-3.6)	2.87E-02
394	Rheumatic disease of the heart valves	CVDD		10	10	3.2 (1.61-6.36)	9.06E-04
394.2	Mitral valve disease	CVDD		18	18	8.85 (5.09-15.4)	1.20E-14
395.1	Nonrheumatic mitral valve disorders	CVDD		13	13	3.01 (1.58-5.72)	7.79E-04
401.2	Hypertensive heart and/or renal disease	CVDD		11	11	21.22 (11.01-40.91)	7.29E-20
41	Bacterial infection NOS	CVDD		13	13	2.36 (1.24-4.48)	8.79E-03
411.2	Myocardial infarction	CVDD		19	19	6.63 (4.09-10.76)	1.77E-14
411.4	Coronary atherosclerosis	CVDD		23	23	5.91 (3.78-9.22)	5.71E-15
411.8	Other chronic ischemic heart disease, unspecified	CVDD		21	21	3.95 (2.53-6.16)	1.36E-09
414	Other forms of chronic heart disease	CVDD		16	16	3.96 (2.33-6.74)	3.72E-07
415.2	Chronic pulmonary heart disease	CVDD		15	15	7.39 (3.82-14.31)	3.02E-09
416	Cardiomegaly	CVDD		22	22	4.11 (2.51-6.74)	1.98E-08
426.3	Bundle branch block	CVDD		16	16	2.28 (1.28-4.05)	5.16E-03
427.2	Atrial fibrillation and flutter	CVDD		25	25	2.34 (1.44-3.81)	5.82E-04
428	Heart failure	CVDD		43	43	8.91 (5.88-13.5)	6.92E-25
433.2	Occlusion of cerebral arteries	CVDD		28	28	8.72 (5.47-13.9)	8.81E-20
443.9	Peripheral vascular disease, unspecified	CVDD		14	14	3.95 (2.09-7.45)	2.29E-05
458.9	Hypotension NOS	CVDD		20	20	2.86 (1.69-4.84)	9.17E-05
480	Pneumonia	CVDD		30	30	3.78 (2.48-5.78)	7.28E-10
480.1	Bacterial pneumonia	CVDD		25	25	1.99 (1.24-3.19)	4.42E-03
496.2	Chronic bronchitis	CVDD		16	16	2.15 (1.19-3.88)	1.15E-02
501	Pneumonitis due to inhalation of food or vomitus	CVDD		11	11	5.36 (2.66-10.81)	2.68E-06
507	Pleurisy; pleural effusion	CVDD		31	31	3.73 (2.39-5.81)	6.02E-09
509.1	Respiratory failure	CVDD		21	21	3.39 (1.99-5.78)	7.07E-06
519.8	Other diseases of respiratory system, NEC	CVDD		18	18	2.38 (1.4-4.05)	1.34E-03
585.1	Acute renal failure	CVDD		48	48	4.26 (2.88-6.31)	4.64E-13
585.3	Chronic renal failure [CKD]	CVDD		35	35	3.24 (2.11-4.97)	7.36E-08
591	Urinary tract infection	CVDD		31	31	1.97 (1.27-3.06)	2.34E-03
707.1	Decubitus ulcer	CVDD		16	16	3.48 (1.91-6.32)	4.45E-05
8	Intestinal infection	CVDD		22	22	2.22 (1.36-3.64)	1.54E-03
994.2	Sepsis	CVDD		19	19	2.37 (1.42-3.95)	9.08E-04

280.1	Iron deficiency anemias	276.1	Electrolyte imbalance	35	49	2.85 (2.06-3.94)	8.37E-09
401.2	Hypertensive heart and/or renal disease	585.3	Chronic renal failure [CKD]	22	27	12.71 (6.85-23.61)	2.88E-14
411.2	Myocardial infarction	411.8	Other chronic ischemic heart disease, unspecified	53	81	22.86 (16.08-32.5)	1.44E-66
411.2	Myocardial infarction	480	Pneumonia	23	30	3.29 (2.18-4.96)	5.57E-07
411.4	Coronary atherosclerosis	276.1	Electrolyte imbalance	39	55	3.37 (2.41-4.71)	4.56E-11
411.4	Coronary atherosclerosis	276.6	Fluid overload	20	22	3.58 (2.07-6.19)	1.80E-04
411.4	Coronary atherosclerosis	411.8	Other chronic ischemic heart disease, unspecified	87	112	25.31 (18.47-34.68)	2.24E-88
411.4	Coronary atherosclerosis	458.9	Hypotension NOS	29	32	3.7 (2.47-5.54)	8.39E-09
411.4	Coronary atherosclerosis	507	Pleurisy; pleural effusion	31	40	3.16 (2.19-4.56)	2.94E-08
411.4	Coronary atherosclerosis	585.1	Acute renal failure	49	64	3.09 (2.26-4.22)	6.93E-11
411.4	Coronary atherosclerosis	585.3	Chronic renal failure [CKD]	40	58	3.36 (2.42-4.66)	1.40E-11
411.8	Other chronic ischemic heart disease, unspecified	251.1	Hypoglycemia	22	29	7.46 (4.31-12.9)	2.34E-11
411.8	Other chronic ischemic heart disease, unspecified	416	Cardiomegaly	26	35	3.81 (2.57-5.67)	1.20E-09
411.8	Other chronic ischemic heart disease, unspecified	585.1	Acute renal failure	46	69	2.73 (2.02-3.68)	2.01E-09
411.8	Other chronic ischemic heart disease, unspecified	585.3	Chronic renal failure [CKD]	48	69	4.1 (3.04-5.52)	7.41E-19
426.3	Bundle branch block	480	Pneumonia	25	30	4.37 (2.89-6.61)	9.15E-11
427.2	Atrial fibrillation and flutter	585.3	Chronic renal failure [CKD]	50	75	4.36 (3.25-5.86)	4.63E-21
428	Heart failure	480	Pneumonia	54	72	7.77 (5.63-10.71)	2.97E-34
428	Heart failure	707.1	Decubitus ulcer	21	27	5.5 (3.37-8.96)	2.84E-10
433.2	Occlusion of cerebral arteries	480	Pneumonia	24	29	4.74 (3.03-7.4)	2.84E-10
480.1	Bacterial pneumonia	480	Pneumonia	61	93	11.03 (8.09-15.04)	1.44E-50
480.1	Bacterial pneumonia	509.1	Respiratory failure	36	50	16.07 (11.11-23.25)	1.28E-47
507	Pleurisy; pleural effusion	480	Pneumonia	41	60	7.01 (5.05-9.74)	1.03E-29
585.1	Acute renal failure	276.6	Fluid overload	29	39	17.53 (10.41-29.52)	1.78E-25
585.1	Acute renal failure	480	Pneumonia	52	77	6.72 (4.99-9.05)	2.08E-34
585.1	Acute renal failure	707.1	Decubitus ulcer	35	43	14.25 (9.14-22.24)	3.96E-30
585.3	Chronic renal failure [CKD]	480	Pneumonia	42	61	3.83 (2.78-5.27)	7.39E-15
585.3	Chronic renal failure [CKD]	707.1	Decubitus ulcer	25	30	4.24 (2.68-6.73)	2.73E-08
591	Urinary tract infection	414	Other forms of chronic heart disease	20	25	2.11 (1.35-3.31)	3.72E-02
591	Urinary tract infection	416	Cardiomegaly	25	33	2.14 (1.42-3.22)	9.96E-03
591	Urinary tract infection	480	Pneumonia	40	56	3.28 (2.38-4.53)	1.67E-11
591	Urinary tract infection	707.1	Decubitus ulcer	35	38	9.39 (6.01-14.68)	3.15E-21
591	Urinary tract infection	994.2	Sepsis	34	49	8.34 (6.02-11.56)	1.10E-35
8	Intestinal infection	480	Pneumonia	38	53	4.02 (2.87-5.63)	1.96E-14
**RSDD**							
276.1	Electrolyte imbalance	RSDD		18	18	3.71 (2.05-6.73)	1.56E-05
276.4	Acid-base balance disorder	RSDD		13	13	10.45 (5.12-21.3)	1.08E-10
276.5	Hypovolemia	RSDD		14	14	3.34 (1.77-6.3)	1.98E-04
276.6	Fluid overload	RSDD		16	16	11.52 (5.91-22.45)	7.08E-13

300.1	Anxiety disorder	RSDD		15	15	3.72 (2.02-6.84)	2.36E-05
41	Bacterial infection NOS	RSDD		11	11	4.43 (2.16-9.06)	4.73E-05
41.4	E. coli	RSDD		12	12	4.44 (2.25-8.8)	1.86E-05
411.8	Other chronic ischemic heart disease, unspecified	RSDD		10	10	2.15 (1.11-4.16)	2.38E-02
415.2	Chronic pulmonary heart disease	RSDD		11	11	10.6 (4.96-22.66)	1.13E-09
427.2	Atrial fibrillation and flutter	RSDD		16	16	3.04 (1.62-5.71)	5.25E-04
428	Heart failure	RSDD		15	15	3.37 (1.74-6.51)	3.02E-04
458.9	Hypotension NOS	RSDD		10	10	2.98 (1.45-6.12)	3.03E-03
480	Pneumonia	RSDD		25	25	15.08 (8.81-25.82)	4.52E-23
480.1	Bacterial pneumonia	RSDD		29	29	13.2 (7.5-23.2)	3.28E-19
496	Chronic airway obstruction	RSDD		14	14	4.46 (2.34-8.48)	5.34E-06
496.2	Chronic bronchitis	RSDD		13	13	6.54 (3.31-12.92)	6.21E-08
507	Pleurisy; pleural effusion	RSDD		14	14	2.94 (1.57-5.53)	8.04E-04
508	Pulmonary collapse; interstitial and compensatory emphysema	RSDD		15	15	6.46 (3.37-12.38)	1.93E-08
509.1	Respiratory failure	RSDD		20	20	12 (6.67-21.59)	1.09E-16
519.8	Other diseases of respiratory system, NEC	RSDD		14	14	5.3 (2.85-9.85)	1.33E-07
563	Constipation	RSDD		19	19	3.13 (1.79-5.45)	6.01E-05
585.1	Acute renal failure	RSDD		19	19	2.63 (1.48-4.67)	9.52E-04
585.3	Chronic renal failure [CKD]	RSDD		17	17	2.77 (1.52-5.04)	8.31E-04
591	Urinary tract infection	RSDD		15	15	1.95 (1.06-3.59)	3.16E-02
707.1	Decubitus ulcer	RSDD		14	14	8.08 (4.1-15.92)	1.61E-09
994.2	Sepsis	RSDD		14	14	3.2 (1.69-6.08)	3.68E-04
300.1	Anxiety disorder	276.5	Hypovolemia	21	27	2.24 (1.48-3.39)	2.88E-03
300.1	Anxiety disorder	509.1	Respiratory failure	20	24	2.61 (1.65-4.12)	8.56E-04
300.1	Anxiety disorder	585.1	Acute renal failure	35	48	2.01 (1.45-2.8)	7.62E-04
300.1	Anxiety disorder	994.2	Sepsis	21	26	2.03 (1.33-3.11)	2.59E-02
411.8	Other chronic ischemic heart disease, unspecified	585.3	Chronic renal failure [CKD]	48	69	4.1 (3.04-5.52)	4.74E-19
427.2	Atrial fibrillation and flutter	585.3	Chronic renal failure [CKD]	50	75	4.36 (3.25-5.86)	2.96E-21
428	Heart failure	480	Pneumonia	54	72	7.77 (5.63-10.71)	1.90E-34
428	Heart failure	707.1	Decubitus ulcer	21	27	5.5 (3.37-8.96)	1.81E-10
480.1	Bacterial pneumonia	480	Pneumonia	61	93	11.03 (8.09-15.04)	9.19E-51
480.1	Bacterial pneumonia	509.1	Respiratory failure	36	50	16.07 (11.11-23.25)	8.15E-48
496	Chronic airway obstruction	276.1	Electrolyte imbalance	30	39	2.23 (1.54-3.23)	5.32E-04
496	Chronic airway obstruction	585.1	Acute renal failure	44	62	2.54 (1.83-3.54)	7.90E-07
507	Pleurisy; pleural effusion	480	Pneumonia	41	60	7.01 (5.05-9.74)	6.56E-30
585.1	Acute renal failure	276.6	Fluid overload	29	39	17.53 (10.41-29.52)	1.14E-25
585.1	Acute renal failure	480	Pneumonia	52	77	6.72 (4.99-9.05)	1.33E-34
585.1	Acute renal failure	707.1	Decubitus ulcer	35	43	14.25 (9.14-22.24)	2.53E-30

585.3	Chronic renal failure [CKD]	276.5	Hypovolemia	31	41	3.51 (2.46-5)	8.10E-11
585.3	Chronic renal failure [CKD]	480	Pneumonia	42	61	3.83 (2.78-5.27)	4.72E-15
585.3	Chronic renal failure [CKD]	707.1	Decubitus ulcer	25	30	4.24 (2.68-6.73)	1.74E-08
591	Urinary tract infection	41.4	E. coli	33	38	120.15 (71-203.33)	7.75E-70
591	Urinary tract infection	480	Pneumonia	40	56	3.28 (2.38-4.53)	1.07E-11
591	Urinary tract infection	707.1	Decubitus ulcer	35	38	9.39 (6.01-14.68)	2.01E-21
**MND**							
112	Candidiasis	MND		31	31	5.54 (3.55-8.65)	5.07E-14
275.3	Disorders of magnesium metabolism	MND		17	17	3.03 (1.73-5.28)	9.64E-05
275.5	Disorders of calcium/phosphorus metabolism	MND		29	29	4.68 (2.98-7.35)	2.28E-11
276.1	Electrolyte imbalance	MND		59	59	3.1 (2.25-4.29)	6.22E-12
276.4	Acid-base balance disorder	MND		16	16	3.16 (1.8-5.58)	6.69E-05
276.5	Hypovolemia	MND		48	48	4.29 (2.99-6.16)	2.96E-15
285	Other anemias	MND		61	61	3.11 (2.26-4.27)	2.28E-12
285.2	Anemia of chronic disease	MND		10	10	4.7 (2.2-10.04)	6.56E-05
287.3	Thrombocytopenia	MND		18	18	5 (2.84-8.82)	2.68E-08
288.11	Neutropenia	MND		31	31	11.05 (6.81-17.93)	2.14E-22
41	Bacterial infection NOS	MND		18	18	1.92 (1.13-3.25)	1.51E-02
41.2	Streptococcus infection	MND		12	12	3.4 (1.76-6.56)	2.74E-04
41.4	E. coli	MND		23	23	2.31 (1.44-3.72)	5.37E-04
458.9	Hypotension NOS	MND		37	37	3.38 (2.3-4.97)	6.61E-10
480	Pneumonia	MND		28	28	1.96 (1.33-2.88)	6.86E-04
480.1	Bacterial pneumonia	MND		59	59	3.66 (2.63-5.08)	1.04E-14
501	Pneumonitis due to inhalation of food or vomitus	MND		12	12	2.68 (1.38-5.22)	3.68E-03
507	Pleurisy; pleural effusion	MND		70	70	6.44 (4.67-8.88)	6.35E-30
508	Pulmonary collapse; interstitial and compensatory emphysema	MND		27	27	2.7 (1.72-4.24)	1.68E-05
509.1	Respiratory failure	MND		25	25	2.33 (1.47-3.68)	2.90E-04
519.8	Other diseases of respiratory system, NEC	MND		29	29	2.47 (1.64-3.71)	1.39E-05
563	Constipation	MND		60	60	3.12 (2.27-4.29)	2.26E-12
585.1	Acute renal failure	MND		71	71	3.32 (2.45-4.5)	1.12E-14
591	Urinary tract infection	MND		57	57	2.41 (1.75-3.33)	7.88E-08
707.1	Decubitus ulcer	MND		22	22	3.06 (1.86-5.03)	1.04E-05
8	Intestinal infection	MND		41	41	2.15 (1.49-3.09)	3.72E-05
994.2	Sepsis	MND		54	54	5.68 (4.01-8.05)	1.70E-22
285	Other anemias	480	Pneumonia	40	48	2.81 (2-3.96)	2.91E-08
285	Other anemias	707.1	Decubitus ulcer	23	29	5.17 (3.28-8.15)	1.64E-11
480.1	Bacterial pneumonia	480	Pneumonia	61	93	11.03 (8.09-15.04)	4.00E-51
480.1	Bacterial pneumonia	509.1	Respiratory failure	36	50	16.07 (11.11-23.25)	3.54E-48

585.1	Acute renal failure	480	Pneumonia	52	77	6.72 (4.99-9.05)	5.77E-35
585.1	Acute renal failure	707.1	Decubitus ulcer	35	43	14.25 (9.14-22.24)	1.10E-30
591	Urinary tract infection	41.4	E. coli	33	38	120.15 (71-203.33)	3.37E-70
591	Urinary tract infection	480	Pneumonia	40	56	3.28 (2.38-4.53)	4.65E-12
591	Urinary tract infection	707.1	Decubitus ulcer	35	38	9.39 (6.01-14.68)	8.76E-22
8	Intestinal infection	480	Pneumonia	38	53	4.02 (2.87-5.63)	5.45E-15

OR = Odds ratio; CI = Confidence interval.

CVDD = Cardiovascular disease death; RSDD = Respiratory system disease death; MND = Malignant neoplasms death; DSDD = Digestive system disease death.

All p-value reported has been adjusted using Bonferroni correction.

Table S9. Mediation effect of D2 within a length-3 disease trajectory D1→D2→D3 in disease trajectories leading to death.

D1	D2	D3	Direct effect		Indirect effect		Total effect		Mediated proportion
			Effect	P-value	Effect	P-value	Effect	P-value	
**CVDD**									
242	411.2	411.8	0.016	<0.001	0.0026	0.012	0.018	<0.001	14.5%
242	411.4	411.8	0.014	<0.001	0.004	0.004	0.018	<0.001	21.7%
242	401.2	585.3	0.021	<0.001	0.0025	<0.001	0.024	<0.001	10.6%
242	411.4	507	0.014	<0.001	0.00097	<0.001	0.015	<0.001	6.3%
242	427.2	585.3	0.023	<0.001	0.0011	0.032	0.024	<0.001	4.6%
242	280.1	276.1	0.019	<0.001	0.0024	<0.001	0.021	<0.001	11.6%
242	411.4	276.1	0.02	<0.001	0.00083	0.004	0.021	<0.001	3.9%
242	411.4	458.9	0.012	<0.001	0.00074	<0.001	0.013	<0.001	5.6%
242	426.3	480	0.017	<0.001	0.0015	<0.001	0.018	<0.001	8.4%
242	428	480	0.014	<0.001	0.0041	<0.001	0.018	<0.001	22.5%
242	428	707.1	0.0069	<0.001	0.0019	<0.001	0.0088	<0.001	21.7%
242	433.2	480	0.017	<0.001	0.00079	<0.001	0.018	<0.001	4.4%
242	480.1	480	0.013	<0.001	0.0047	<0.001	0.018	<0.001	26.2%
242	480.1	509.1	0.007	<0.001	0.004	<0.001	0.011	<0.001	36.6%
242	591	414	0.012	<0.001	0.0011	<0.001	0.013	<0.001	8.3%
242	591	416	0.015	<0.001	0.0014	<0.001	0.016	<0.001	8.8%
242	591	480	0.015	<0.001	0.0032	<0.001	0.018	<0.001	17.9%
242	591	707.1	0.0064	<0.001	0.0024	<0.001	0.0088	<0.001	27.3%
242	591	994.2	0.013	<0.001	0.0039	<0.001	0.017	<0.001	23.1%
242	8	480	0.016	<0.001	0.0025	<0.001	0.018	<0.001	13.6%
411.2	411.8	585.1	0.11	<0.001	0.042	<0.001	0.15	<0.001	28.1%
411.2	411.8	585.3	0.083	<0.001	0.052	<0.001	0.13	<0.001	38.2%
411.4	411.8	585.1	0.069	<0.001	0.043	<0.001	0.11	<0.001	38.7%
411.4	411.8	585.3	0.067	<0.001	0.05	<0.001	0.12	<0.001	42.9%
401.2	585.3	480	0.11	<0.001	0.044	<0.001	0.16	<0.001	28.0%
401.2	585.3	707.1	0.056	0.02	0.027	<0.001	0.083	<0.001	32.6%
411.2	411.8	251.1	0.021	<0.001	0.012	<0.001	0.033	<0.001	36.9%
411.2	411.8	416	0.084	<0.001	0.028	<0.001	0.11	<0.001	25.3%
411.4	411.8	251.1	0.008	0.152	0.013	<0.001	0.021	<0.001	62.8%
411.4	411.8	416	0.087	<0.001	0.024	<0.001	0.11	<0.001	21.8%
411.4	507	480	0.04	<0.001	0.019	<0.001	0.059	<0.001	32.2%
427.2	585.3	480	0.087	<0.001	0.012	<0.001	0.1	<0.001	12.2%
427.2	585.3	707.1	0.04	<0.001	0.0077	<0.001	0.048	<0.001	16.1%
280.1	276.1	CVDD	0.024	<0.001	0.006	<0.001	0.03	<0.001	20.3%

411.4	276.1	CVDD	0.092	<0.001	0.004	<0.001	0.096	<0.001	4.1%
411.4	458.9	CVDD	0.094	<0.001	0.0028	<0.001	0.096	<0.001	2.9%
426.3	480	CVDD	0.049	<0.001	0.0086	<0.001	0.057	<0.001	14.9%
428	480	CVDD	0.13	<0.001	0.0077	<0.001	0.14	<0.001	5.4%
428	707.1	CVDD	0.14	<0.001	0.0045	<0.001	0.14	<0.001	3.1%
433.2	480	CVDD	0.13	<0.001	0.0061	<0.001	0.14	<0.001	4.5%
480.1	480	CVDD	0.034	<0.001	0.015	<0.001	0.048	<0.001	30.2%
480.1	509.1	CVDD	0.035	<0.001	0.013	<0.001	0.048	<0.001	26.8%
591	414	CVDD	0.038	<0.001	0.0034	<0.001	0.041	<0.001	8.3%
591	416	CVDD	0.037	<0.001	0.0042	<0.001	0.041	<0.001	10.2%
591	480	CVDD	0.034	<0.001	0.0074	<0.001	0.041	<0.001	18.0%
591	707.1	CVDD	0.035	<0.001	0.0063	<0.001	0.041	<0.001	15.3%
591	994.2	CVDD	0.037	<0.001	0.0046	<0.001	0.041	<0.001	11.2%
8	480	CVDD	0.02	<0.001	0.0074	<0.001	0.027	<0.001	27.2%
411.8	585.1	276.6	0.023	<0.001	0.011	<0.001	0.033	<0.001	32.4%
411.8	585.1	480	0.064	<0.001	0.024	<0.001	0.088	<0.001	27.6%
411.8	585.1	707.1	0.023	<0.001	0.015	<0.001	0.038	<0.001	39.3%
411.8	585.3	480	0.075	<0.001	0.013	<0.001	0.088	<0.001	15.0%
411.8	585.3	707.1	0.03	<0.001	0.0084	<0.001	0.038	<0.001	22.1%
585.3	480	CVDD	0.046	<0.001	0.0061	<0.001	0.052	<0.001	11.8%
585.3	707.1	CVDD	0.047	<0.001	0.0044	<0.001	0.052	<0.001	8.5%
411.8	251.1	CVDD	0.091	<0.001	0.004	<0.001	0.095	<0.001	4.2%
411.8	416	CVDD	0.089	<0.001	0.0062	<0.001	0.095	<0.001	6.5%
507	480	CVDD	0.051	<0.001	0.011	<0.001	0.063	<0.001	18.2%
585.1	276.6	CVDD	0.067	<0.001	0.009	<0.001	0.076	<0.001	11.8%
585.1	480	CVDD	0.067	<0.001	0.0094	<0.001	0.076	<0.001	12.3%
585.1	707.1	CVDD	0.07	<0.001	0.0062	<0.001	0.076	<0.001	8.1%
**RSDD**									
242	300.1	585.1	0.029	<0.001	0.00086	<0.001	0.03	<0.001	2.9%
242	411.8	585.3	0.021	<0.001	0.0027	<0.001	0.024	<0.001	11.4%
242	427.2	585.3	0.023	<0.001	0.0011	0.016	0.024	<0.001	4.6%
242	496	585.1	0.028	<0.001	0.0015	<0.001	0.03	<0.001	5.0%
242	300.1	276.5	0.015	<0.001	0.00055	<0.001	0.015	<0.001	3.6%
242	300.1	509.1	0.011	<0.001	0.00044	<0.001	0.011	<0.001	4.0%
242	300.1	994.2	0.016	<0.001	0.00036	<0.001	0.017	<0.001	2.1%
242	428	480	0.014	<0.001	0.0041	<0.001	0.018	<0.001	22.5%
242	428	707.1	0.0069	<0.001	0.0019	<0.001	0.0088	<0.001	21.7%
242	480.1	480	0.013	<0.001	0.0047	<0.001	0.018	<0.001	26.2%
242	480.1	509.1	0.007	0.004	0.004	<0.001	0.011	<0.001	36.6%

242	496	276.1	0.02	<0.001	0.0013	<0.001	0.021	<0.001	6.2%
242	507	480	0.015	<0.001	0.003	<0.001	0.018	<0.001	16.5%
242	591	41.4	0.0056	0.008	0.0075	<0.001	0.013	<0.001	57.2%
242	591	480	0.015	<0.001	0.0032	<0.001	0.018	<0.001	17.9%
242	591	707.1	0.0064	<0.001	0.0024	<0.001	0.0088	<0.001	27.3%
300.1	585.1	276.6	0.016	<0.001	0.0056	<0.001	0.022	<0.001	26.1%
300.1	585.1	480	0.028	<0.001	0.013	<0.001	0.041	<0.001	30.9%
300.1	585.1	707.1	0.013	0.008	0.0077	<0.001	0.021	<0.001	37.2%
411.8	585.3	276.5	0.053	<0.001	0.015	<0.001	0.068	<0.001	21.4%
411.8	585.3	480	0.075	<0.001	0.013	<0.001	0.088	<0.001	15.0%
411.8	585.3	707.1	0.03	<0.001	0.0084	<0.001	0.038	<0.001	22.1%
427.2	585.3	276.5	0.045	<0.001	0.014	<0.001	0.059	<0.001	23.9%
427.2	585.3	480	0.087	<0.001	0.012	<0.001	0.1	<0.001	12.2%
427.2	585.3	707.1	0.04	<0.001	0.0077	<0.001	0.048	<0.001	16.1%
496	585.1	276.6	0.016	0.004	0.011	<0.001	0.027	<0.001	39.9%
496	585.1	480	0.1	<0.001	0.023	<0.001	0.13	<0.001	18.1%
496	585.1	707.1	0.022	<0.001	0.014	<0.001	0.037	<0.001	39.5%
300.1	276.5	RSDD	0.023	<0.001	0.0019	<0.001	0.025	<0.001	7.5%
300.1	509.1	RSDD	0.02	<0.001	0.0057	<0.001	0.025	<0.001	22.6%
300.1	994.2	RSDD	0.024	<0.001	0.0013	<0.001	0.025	<0.001	5.1%
428	480	RSDD	0.032	<0.001	0.02	<0.001	0.052	<0.001	38.2%
428	707.1	RSDD	0.046	<0.001	0.006	<0.001	0.052	<0.001	11.5%
480.1	480	RSDD	0.055	<0.001	0.024	<0.001	0.079	<0.001	30.8%
480.1	509.1	RSDD	0.047	<0.001	0.031	<0.001	0.079	<0.001	39.8%
496	276.1	RSDD	0.056	<0.001	0.0036	<0.001	0.06	<0.001	6.1%
507	480	RSDD	0.013	0.04	0.022	<0.001	0.035	<0.001	63.1%
591	41.4	RSDD	0.011	0.008	0.0087	0.008	0.02	<0.001	44.6%
591	480	RSDD	0.0061	0.144	0.013	<0.001	0.02	<0.001	68.6%
591	707.1	RSDD	0.013	0.004	0.0066	<0.001	0.02	<0.001	34.0%
585.1	276.6	RSDD	0.024	<0.001	0.0074	<0.001	0.032	<0.001	23.2%
585.1	480	RSDD	0.011	0.02	0.021	<0.001	0.032	<0.001	65.9%
585.1	707.1	RSDD	0.024	<0.001	0.008	<0.001	0.032	<0.001	25.1%
585.3	276.5	RSDD	0.017	<0.001	0.0045	<0.001	0.022	<0.001	20.3%
585.3	480	RSDD	0.011	0.012	0.011	<0.001	0.022	<0.001	50.3%
585.3	707.1	RSDD	0.017	<0.001	0.0046	<0.001	0.022	<0.001	20.9%
**MND**									
242	285	480	0.015	<0.001	0.003	<0.001	0.018	<0.001	16.6%
242	285	707.1	0.0069	0.004	0.0019	<0.001	0.0088	<0.001	21.2%
242	480.1	480	0.013	<0.001	0.0047	<0.001	0.018	<0.001	26.2%

242	480.1	509.1	0.007	<0.001	0.004	<0.001	0.011	<0.001	36.6%
242	585.1	480	0.012	<0.001	0.0056	<0.001	0.018	<0.001	31.1%
242	585.1	707.1	0.0054	0.008	0.0034	<0.001	0.0088	<0.001	38.7%
242	591	41.4	0.0056	0.008	0.0075	<0.001	0.013	<0.001	57.2%
242	591	480	0.015	<0.001	0.0032	<0.001	0.018	<0.001	17.9%
242	591	707.1	0.0064	<0.001	0.0024	<0.001	0.0088	<0.001	27.3%
242	8	480	0.016	<0.001	0.0025	<0.001	0.018	<0.001	13.6%
285	480	MND	0.095	<0.001	0.013	<0.001	0.11	<0.001	12.3%
285	707.1	MND	0.1	<0.001	0.0079	<0.001	0.11	<0.001	7.3%
480.1	480	MND	0.11	<0.001	0.026	<0.001	0.13	<0.001	19.9%
480.1	509.1	MND	0.13	<0.001	0.0068	0.024	0.13	<0.001	5.2%
585.1	480	MND	0.11	<0.001	0.02	<0.001	0.13	<0.001	15.5%
585.1	707.1	MND	0.12	<0.001	0.01	<0.001	0.13	<0.001	8.0%
591	41.4	MND	0.068	<0.001	0.015	<0.001	0.084	<0.001	18.0%
591	480	MND	0.069	<0.001	0.015	<0.001	0.084	<0.001	17.8%
591	707.1	MND	0.073	<0.001	0.01	<0.001	0.084	<0.001	12.3%
8	480	MND	0.064	<0.001	0.014	<0.001	0.079	<0.001	18.4%

CI = Confidence interval.

CVDD = Cardiovascular disease death; RSDD = Respiratory system disease death; MND = Malignant neoplasms death; DSDD = Digestive system disease death.

All p-value reported has been adjusted using Bonferroni correction.

Subgroup and Sensitivity Analysis

Table S10. Associations between prior diagnosed hyperthyroidism and subsequent diseases stratified by gender.

		Overall (N = 28,411, HT cases = 5,832)		Males (N = 6,549, HT cases = 1,337)		Females (N = 21,862, HT cases = 4,495)	
PheCode	Phenotype	N (HT population)	HR (95% CI)	N (HT population)	HR (95% CI)	N (HT population)	HR (95% CI)
**Cardiovascular Diseases**							
427.2	Atrial fibrillation and flutter	369	1.32 (1.17-1.49)	107	1.31 (1.06-1.62)	262	1.5 (1.3-1.72)
411.8	Other chronic ischemic heart disease, unspecified	355	1.57 (1.39-1.79)	142	1.76 (1.45-2.13)	213	1.71 (1.46-2)
411.4	Coronary atherosclerosis	300	1.33 (1.17-1.52)	125	1.49 (1.22-1.82)	175	1.4 (1.18-1.66)
428	Heart failure	318	1.81 (1.58-2.08)	122	2.11 (1.7-2.62)	196	1.9 (1.61-2.25)
411.2	Myocardial infarction	215	1.31 (1.12-1.53)	77	1.32 (1.03-1.7)	138	1.51 (1.24-1.83)
458.9	Hypotension NOS	223	1.67 (1.42-1.96)	66	1.77 (1.33-2.35)	157	1.7 (1.41-2.05)
426.3	Bundle branch block	178	1.73 (1.45-2.07)	77	2.28 (1.73-3.01)	101	1.6 (1.27-2.01)
433.3	Cerebral ischemia	162	1.59 (1.32-1.92)	49	1.77 (1.27-2.48)	113	1.67 (1.34-2.07)
416	Cardiomegaly	193	2.02 (1.69-2.41)	80	2.9 (2.2-3.84)	113	1.97 (1.57-2.46)
433.2	Occlusion of cerebral arteries	153	1.49 (1.23-1.8)	50	1.83 (1.32-2.55)	103	1.53 (1.22-1.92)
414	Other forms of chronic heart disease	163	1.88 (1.55-2.27)	69	2.64 (1.96-3.56)	94	1.79 (1.41-2.28)
458.1	Orthostatic hypotension	119	1.51 (1.22-1.87)	37	1.58 (1.08-2.31)	82	1.6 (1.24-2.06)
395.1	Nonrheumatic mitral valve disorders	134	1.99 (1.61-2.46)	39	2.16 (1.47-3.18)	95	2.11 (1.65-2.7)
426.2	Atrioventricular [AV] block	115	1.72 (1.38-2.16)	50	2.06 (1.47-2.88)	65	1.72 (1.29-2.3)
443.9	Peripheral vascular disease, unspecified	92	1.69 (1.32-2.17)	36	2.18 (1.46-3.25)	56	1.64 (1.2-2.24)
394	Rheumatic disease of the heart valves	101	2.02 (1.58-2.58)	33	2.42 (1.58-3.71)	68	1.99 (1.49-2.65)
394.2	Mitral valve disease	99	1.86 (1.46-2.37)	34	2.79 (1.81-4.3)	65	1.77 (1.33-2.37)
427.1	Paroxysmal tachycardia, unspecified	89	1.69 (1.31-2.18)	34	2.37 (1.57-3.6)	55	1.58 (1.16-2.16)
415.2	Chronic pulmonary heart disease	72	1.92 (1.44-2.55)	20	1.72 (1.02-2.89)	52	2.24 (1.6-3.14)
433.8	Late effects of cerebrovascular disease	69	1.99 (1.48-2.68)	25	2.7 (1.65-4.43)	44	1.87 (1.31-2.66)
420.2	Pericarditis	75	2.82 (2.09-3.82)	23	3.43 (1.99-5.91)	52	2.89 (2.03-4.12)
401.2	Hypertensive heart and/or renal disease	55	2.37 (1.68-3.35)	24	3.11 (1.86-5.19)	31	2.6 (1.65-4.1)
394.7	Disease of tricuspid valve	39	2.52 (1.68-3.78)	9	-	30	2.62 (1.66-4.15)
427.8	Sinoatrial node dysfunction (Bradycardia)	38	2.75 (1.81-4.19)	14	3.95 (1.89-8.25)	24	2.46 (1.48-4.07)
**Digestive Diseases**							
530.1	Esophagitis, GERD and related diseases	639	1.27 (1.16-1.4)	138	1.38 (1.14-1.67)	501	1.28 (1.16-1.42)
550.2	Diaphragmatic hernia	507	1.32 (1.19-1.47)	106	1.56 (1.25-1.94)	401	1.33 (1.19-1.49)
563	Constipation	399	1.33 (1.18-1.49)	97	1.62 (1.29-2.05)	302	1.33 (1.17-1.51)
535	Gastritis and duodenitis	321	1.34 (1.18-1.53)	76	1.79 (1.37-2.34)	245	1.29 (1.11-1.49)
535.8	Other specified gastritis	246	1.45 (1.25-1.68)	57	1.74 (1.28-2.36)	189	1.48 (1.25-1.75)
578.9	Hemorrhage of gastrointestinal tract	207	1.45 (1.23-1.7)	56	1.88 (1.38-2.57)	151	1.37 (1.14-1.65)

558	Noninfectious gastroenteritis	215	1.57 (1.34-1.84)	30	1.29 (0.86-1.94)	185	1.67 (1.41-1.99)
785	Abdominal pain	147	1.47 (1.21-1.77)	19	1.71 (1.01-2.91)	128	1.49 (1.22-1.83)
571.5	Other chronic nonalcoholic liver disease	149	1.73 (1.42-2.1)	47	2.72 (1.89-3.9)	102	1.54 (1.23-1.94)
569	Other disorders of intestine	119	1.62 (1.3-2.01)	29	1.71 (1.11-2.63)	90	1.66 (1.3-2.12)
531.2	Gastric ulcer	109	1.71 (1.36-2.14)	20	1.66 (0.99-2.78)	89	1.75 (1.36-2.24)
550.5	Ventral hernia	83	1.66 (1.28-2.16)	22	2.02 (1.22-3.35)	61	1.68 (1.25-2.26)
575.8	Other disorders of biliary tract	63	2.08 (1.53-2.84)	13	3.67 (1.76-7.63)	50	1.9 (1.36-2.65)
557.1	Celiac disease	53	2.56 (1.8-3.64)	11	1.84 (0.9-3.76)	42	2.51 (1.7-3.71)
577	Diseases of pancreas	45	2.47 (1.69-3.61)	20	4.37 (2.35-8.12)	25	1.91 (1.19-3.06)
555.1	Regional enteritis	32	2.2 (1.42-3.41)	6	-	26	2.4 (1.47-3.9)
441	Vascular insufficiency of intestine	35	2.79 (1.78-4.37)	6	-	29	2.8 (1.75-4.5)
**Ear diseases**							
389	Hearing loss	166	1.44 (1.2-1.72)	47	1.46 (1.05-2.04)	119	1.51 (1.23-1.87)
**Ocular diseases**							
375	Disorders of lacrimal system	78	1.62 (1.24-2.12)	11	1.87 (0.92-3.8)	67	1.67 (1.26-2.22)
374	Other disorders of eyelids	93	2.53 (1.94-3.29)	15	2.28 (1.23-4.24)	78	2.72 (2.04-3.62)
374.3	Ptosis of eyelid	83	2.71 (2.05-3.6)	17	4.48 (2.33-8.62)	66	2.47 (1.82-3.35)
368.2	Diplopia and disorders of binocular vision	52	3.08 (2.14-4.45)	10	2.42 (1.1-5.32)	42	3.46 (2.29-5.23)
378.1	Strabismus (not specified as paralytic)	49	3.98 (2.67-5.92)	14	6.6 (2.88-15.12)	35	3.37 (2.15-5.29)
**Genitourinary Diseases**							
585.1	Acute renal failure	440	1.79 (1.59-2.01)	152	2.1 (1.73-2.54)	288	1.88 (1.64-2.16)
585.3	Chronic renal failure [CKD]	402	1.7 (1.51-1.92)	108	1.96 (1.56-2.45)	294	1.76 (1.53-2.01)
580.2	Nephrotic syndrome without mention of glomerulonephritis	30	2.63 (1.62-4.26)	11	3.3 (1.5-7.25)	19	2.55 (1.43-4.55)
**Hematologic Diseases**							
285	Other anemias	434	1.79 (1.59-2.01)	107	2 (1.59-2.52)	327	1.76 (1.55-2)
280.1	Iron deficiency anemias	364	1.63 (1.44-1.84)	82	1.77 (1.37-2.28)	282	1.7 (1.48-1.95)
288.11	Neutropenia	91	1.61 (1.26-2.06)	22	1.66 (1.02-2.72)	69	1.65 (1.25-2.19)
287.3	Thrombocytopenia	78	2.06 (1.56-2.72)	26	2.81 (1.72-4.58)	52	2 (1.44-2.79)
281.1	Megaloblastic anemia	54	1.88 (1.35-2.61)	14	2 (1.06-3.77)	40	1.94 (1.33-2.82)
285.2	Anemia of chronic disease	39	2.25 (1.51-3.36)	10	3.01 (1.33-6.81)	29	2.17 (1.39-3.41)
**Infectious Diseases**							
591	Urinary tract infection	429	1.77 (1.58-1.99)	123	2.37 (1.91-2.95)	306	1.69 (1.48-1.93)
8	Intestinal infection	358	1.66 (1.46-1.88)	66	1.65 (1.24-2.19)	292	1.74 (1.52-1.99)
480.1	Bacterial pneumonia	323	1.61 (1.41-1.84)	112	2.17 (1.73-2.72)	211	1.55 (1.32-1.81)
480	Pneumonia	277	1.71 (1.48-1.97)	95	2.15 (1.68-2.75)	182	1.68 (1.42-2)
994.2	Sepsis	234	1.86 (1.58-2.17)	83	2.22 (1.71-2.89)	151	1.87 (1.54-2.26)
41	Bacterial infection NOS	150	1.63 (1.35-1.99)	43	1.98 (1.38-2.84)	107	1.61 (1.28-2.01)
41.4	E. coli	168	1.93 (1.6-2.33)	39	3.09 (2.06-4.66)	129	1.86 (1.51-2.28)
79	Viral infection	115	1.54 (1.23-1.92)	31	1.79 (1.18-2.72)	84	1.51 (1.18-1.94)

112	Candidiasis	124	1.74 (1.4-2.15)	30	2.07 (1.34-3.2)	94	1.69 (1.32-2.14)
41.2	Streptococcus infection	67	1.76 (1.32-2.36)	26	2.6 (1.6-4.21)	41	1.49 (1.04-2.13)
136	Other infectious and parasitic diseases	36	2.8 (1.81-4.33)	14	4.49 (2.14-9.43)	22	2.26 (1.34-3.8)
481	Influenza	26	3 (1.79-5.05)	5	-	21	2.74 (1.57-4.78)
**Metabolic Diseases**							
244	Hypothyroid conditions	1,516	14.79 (13.18-16.59)	340	35.11 (27.23-45.27)	1,176	11.83 (10.74-13.03)
250.2	Type 2 diabetes	413	1.3 (1.16-1.46)	127	1.51 (1.24-1.84)	286	1.43 (1.25-1.63)
276.1	Electrolyte imbalance	362	1.64 (1.44-1.85)	95	2.07 (1.62-2.64)	267	1.64 (1.42-1.89)
276.5	Hypovolemia	224	1.79 (1.52-2.1)	69	2.29 (1.71-3.06)	155	1.76 (1.46-2.12)
261.4	Vitamin D deficiency	143	1.77 (1.45-2.17)	25	1.87 (1.17-3)	118	1.8 (1.45-2.23)
275.5	Disorders of calcium/phosphorus metabolism	137	2 (1.62-2.46)	38	2.43 (1.63-3.61)	99	1.98 (1.56-2.51)
276.4	Acid-base balance disorder	92	1.68 (1.31-2.16)	33	2.17 (1.43-3.3)	59	1.64 (1.21-2.21)
275.3	Disorders of magnesium metabolism	101	2.38 (1.84-3.06)	29	3.27 (2.02-5.28)	72	2.5 (1.86-3.34)
261.2	Vitamin B-complex deficiencies	86	2.08 (1.59-2.72)	23	2.32 (1.4-3.84)	63	2.25 (1.66-3.06)
276.6	Fluid overload	98	2.79 (2.14-3.64)	36	3.4 (2.2-5.27)	48	1.83 (1.3-2.56)
251.1	Hypoglycemia	84	2.12 (1.61-2.78)	40	3.68 (2.41-5.63)	58	2.58 (1.86-3.59)
246	Other disorders of thyroid	142	8.64 (6.39-11.67)	39	59.01 (18.15-191.83)	103	6.6 (4.85-8.99)
252.1	Hyperparathyroidism	110	4.68 (3.53-6.2)	21	6.81 (3.43-13.52)	89	4.53 (3.35-6.12)
250.1	Type 1 diabetes	56	2.02 (1.45-2.8)	21	2.49 (1.46-4.22)	35	2.06 (1.37-3.08)
241.2	Nontoxic multinodular goiter	68	8.24 (5.39-12.62)	9	-	59	7.68 (4.99-11.82)
240	Simple and unspecified goiter	54	6.68 (4.27-10.47)	8	-	46	6.44 (4.04-10.27)
241.1	Nontoxic uninodular goiter	32	2.74 (1.73-4.34)	7	-	25	2.26 (1.39-3.68)
252.2	Hypoparathyroidism	51	9.61 (5.72-16.13)	14	15.66 (5.48-44.72)	37	8.38 (4.76-14.73)
227.2	Benign neoplasm of parathyroid gland	36	4.26 (2.64-6.86)	6	-	30	4.09 (2.46-6.79)
255.21	Glucocorticoid deficiency	30	4.55 (2.69-7.69)	8	-	22	4.06 (2.25-7.35)
255	Disorders of adrenal glands	23	2.96 (1.68-5.2)	5	-	18	3.73 (1.98-7.03)
193	Thyroid cancer	21	4.21 (2.26-7.83)	5	-	16	3.36 (1.72-6.55)
245.2	Chronic thyroiditis	20	4.99 (2.55-9.76)	16	32.44 (7.39-142.27)	16	12.91 (4.72-35.29)
253.1	Pituitary hyperfunction	32	20.45 (8.54-48.94)	4	-	26	20.8 (7.98-54.24)
245	Thyroiditis	30	18.99 (7.9-45.66)	0	-	20	5.14 (2.63-10.05)
226	Benign neoplasm of thyroid glands	16	5.82 (2.7-12.54)	4	-	12	4.76 (2.05-11.05)
**Musculoskeletal And Joint Diseases**							
743.11	Osteoporosis NOS	322	1.39 (1.22-1.58)	72	1.66 (1.27-2.18)	239	1.29 (1.11-1.49)
760	Back pain	311	1.32 (1.15-1.5)	23	1.57 (0.97-2.54)	299	1.48 (1.3-1.69)
721	Spondylosis and allied disorders	147	1.49 (1.23-1.8)	19	1.2 (0.72-2)	128	1.64 (1.33-2.01)
**Psychiatric And Behavioral Diseases**							
300.1	Anxiety disorder	363	1.38 (1.22-1.55)	57	1.57 (1.16-2.13)	306	1.35 (1.19-1.54)
290.2	Delirium due to conditions classified elsewhere	154	1.76 (1.45-2.14)	50	1.97 (1.41-2.76)	104	1.8 (1.43-2.27)
**Respiratory Diseases**							

496	Chronic airway obstruction	291	1.4 (1.23-1.61)	83	1.77 (1.38-2.28)	208	1.47 (1.26-1.72)
507	Pleurisy; pleural effusion	278	1.56 (1.35-1.8)	89	1.63 (1.28-2.08)	189	1.67 (1.41-1.98)
519.8	Other diseases of respiratory system, NEC	236	1.51 (1.3-1.76)	68	2.17 (1.62-2.91)	168	1.43 (1.2-1.7)
508	Pulmonary collapse; interstitial and compensatory emphysema	169	1.69 (1.4-2.03)	45	1.67 (1.19-2.36)	124	1.77 (1.43-2.18)
509.1	Respiratory failure	168	1.69 (1.41-2.03)	48	1.74 (1.25-2.43)	120	1.89 (1.52-2.34)
496.2	Chronic bronchitis	160	1.61 (1.33-1.94)	47	1.88 (1.34-2.64)	113	1.71 (1.37-2.13)
327.3	Sleep apnea	129	1.67 (1.36-2.06)	43	1.71 (1.19-2.44)	86	1.77 (1.37-2.29)
479	Other upper respiratory disease	69	1.88 (1.4-2.51)	16	1.88 (1.04-3.4)	53	1.74 (1.26-2.41)
501	Pneumonitis due to inhalation of food or vomitus	67	1.95 (1.44-2.64)	29	2.42 (1.52-3.83)	38	1.75 (1.2-2.56)
**Skin Diseases**							
707.1	Decubitus ulcer	117	1.93 (1.54-2.42)	39	2.22 (1.5-3.27)	78	1.9 (1.46-2.49)
681.7	Cellulitis and abscess of trunk	30	3.49 (2.12-5.75)	13	12.66 (4.39-36.55)	17	2.14 (1.18-3.86)

HT = Hyperthyroidism; HR = Hazard ratio; CI = Confidence interval.

- = The model was unable to accurately estimate the effect due to an insufficient sample size.

Figure S8. Moderation effect of age at diagnosis characterized by restricted cubic spline.

Cox regression was used with a hyperthyroidism (binary variable) × age interaction term, adjusted for year of birth, sex, baseline BMI, Townsend deprivation index, educational attainment, smoking status, alcohol consumption and physical activity. Splines were generated by using the estimated hazard ratios of Cox model with a hyperthyroidism × age interaction term using the R package interactionRCS.

The x-axis represents the age at diagnosis of hyperthyroidism. The left y-axis shows the hazard ratios estimated for subsequent diseases. The right y-axis represents the number of cases for each disease at each age among participants with hyperthyroidism, corresponding to the bar chart

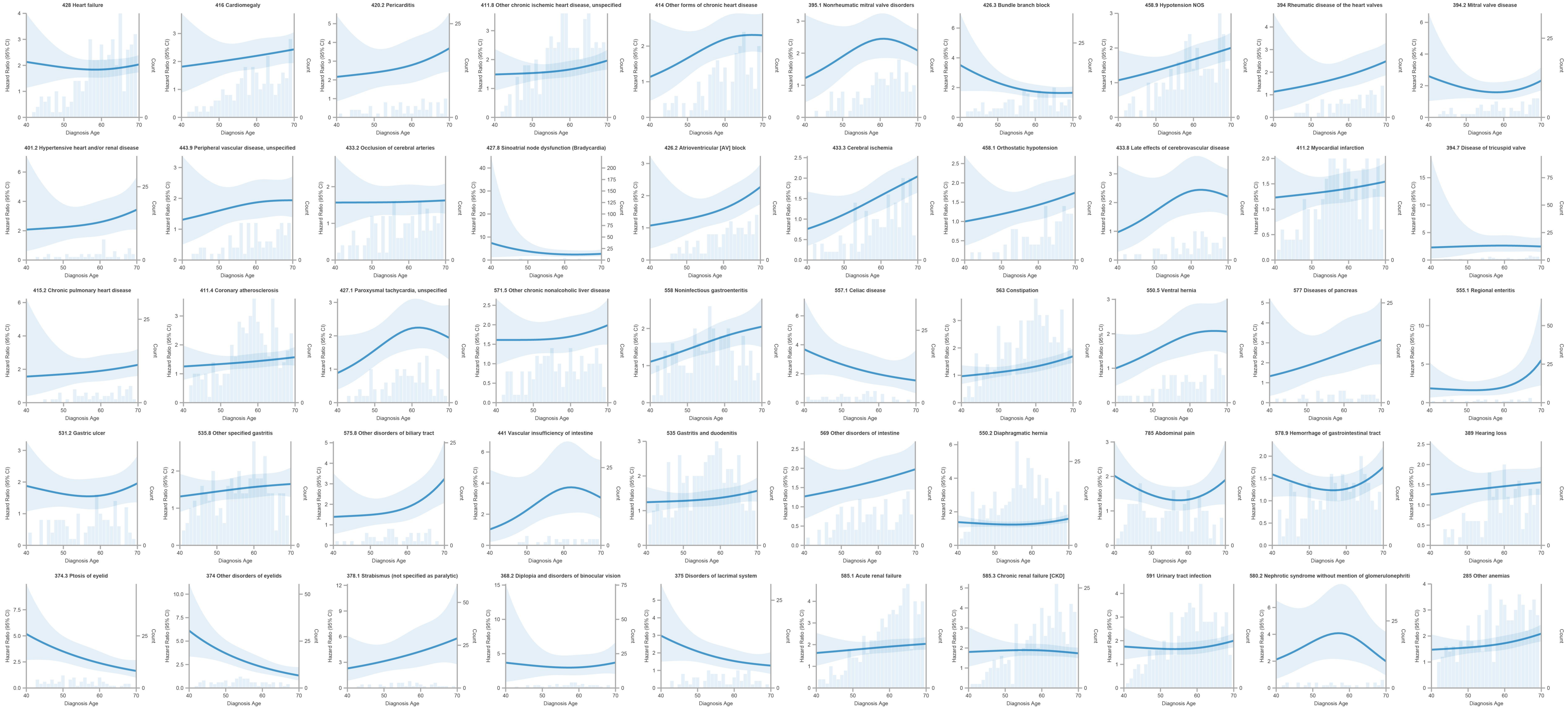

Figure S8 (continued)

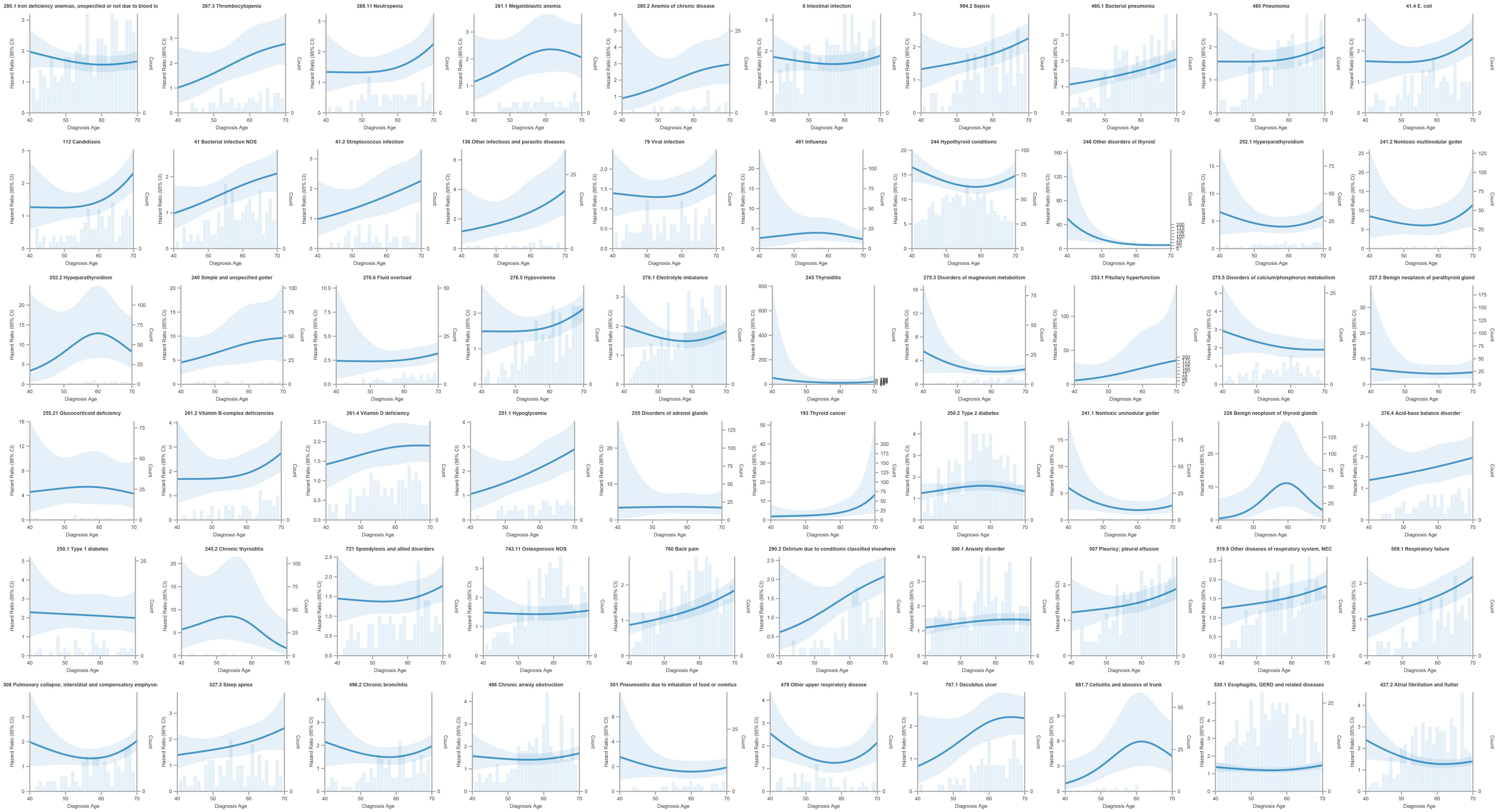

Table S11. Sensitivity analyses to investigate the association between prior diagnosed hyperthyroidism and subsequent diseases with different restrictions.

		Overall (N = 28,411, HT cases = 5,832)		Removing diagnosis records within 6 months following the index date (N = 28,411, HT cases = 5,832)		Subgroup with at least two recorded diagnoses of hyperthyroidism (N = 4,724, HT cases = 961)		Subgroup with no history of other thyroid diseases prior to the index date (N = 19,299, HT cases = 3,952)		Subgroup with no history of malignancy diagnosis prior to the index date (N = 21,540, HT cases = 4,811)	
PheCode	Phenotype	N (HT population)	HR (95% CI)	N (HT population)	HR (95% CI)	N (HT population)	HR (95% CI)	N (HT population)	HR (95% CI)	N (HT population)	HR (95% CI)
**Cardiovascular Diseases**											
427.2	Atrial fibrillation and flutter	369	1.32 (1.17-1.49)	341	1.2 (1.06-1.36)	96	1.9 (1.48-2.44)	238	1.24 (1.06-1.43)	310	1.3 (1.14-1.49)
411.8	Other chronic ischemic heart disease, unspecified	355	1.57 (1.39-1.79)	324	1.41 (1.24-1.61)	78	1.45 (1.11-1.89)	235	1.41 (1.21-1.65)	307	1.59 (1.38-1.82)
411.4	Coronary atherosclerosis	300	1.33 (1.17-1.52)	271	1.21 (1.05-1.39)	83	1.69 (1.3-2.19)	205	1.18 (1.01-1.38)	265	1.37 (1.19-1.59)
428	Heart failure	318	1.81 (1.58-2.08)	278	1.63 (1.42-1.89)	58	1.78 (1.3-2.45)	202	1.73 (1.46-2.05)	257	1.76 (1.51-2.05)
411.2	Myocardial infarction	215	1.31 (1.12-1.53)	196	1.2 (1.02-1.42)	48	1.3 (0.94-1.81)	139	1.13 (0.93-1.36)	181	1.26 (1.06-1.5)
458.9	Hypotension NOS	223	1.67 (1.42-1.96)	193	1.41 (1.19-1.68)	39	1.53 (1.05-2.23)	137	1.45 (1.18-1.76)	168	1.49 (1.24-1.8)
426.3	Bundle branch block	178	1.73 (1.45-2.07)	167	1.66 (1.38-2)	35	1.86 (1.23-2.82)	106	1.48 (1.18-1.86)	147	1.65 (1.35-2.01)
433.3	Cerebral ischemia	162	1.59 (1.32-1.92)	150	1.47 (1.21-1.79)	29	1.65 (1.07-2.56)	95	1.27 (1-1.61)	123	1.39 (1.12-1.72)
416	Cardiomegaly	193	2.02 (1.69-2.41)	179	1.86 (1.55-2.23)	44	2.28 (1.56-3.33)	119	1.87 (1.49-2.33)	158	1.99 (1.63-2.43)
433.2	Occlusion of cerebral arteries	153	1.49 (1.23-1.8)	141	1.41 (1.15-1.72)	37	1.53 (1.04-2.25)	98	1.31 (1.04-1.66)	139	1.53 (1.25-1.87)
414	Other forms of chronic heart disease	163	1.88 (1.55-2.27)	147	1.73 (1.41-2.11)	32	2.52 (1.61-3.95)	97	1.66 (1.3-2.11)	136	1.8 (1.46-2.23)
458.1	Orthostatic hypotension	119	1.51 (1.22-1.87)	109	1.39 (1.1-1.74)	27	1.65 (1.04-2.6)	70	1.31 (0.99-1.73)	87	1.36 (1.05-1.75)
395.1	Nonrheumatic mitral valve disorders	134	1.99 (1.61-2.46)	127	1.93 (1.55-2.4)	32	3.44 (2.13-5.57)	87	2 (1.54-2.61)	111	2.12 (1.67-2.69)
426.2	Atrioventricular [AV] block	115	1.72 (1.38-2.16)	100	1.52 (1.2-1.93)	25	1.79 (1.1-2.91)	75	1.56 (1.19-2.05)	85	1.42 (1.09-1.83)
443.9	Peripheral vascular disease, unspecified	92	1.69 (1.32-2.17)	84	1.52 (1.17-1.98)	17	1.64 (0.92-2.92)	59	1.58 (1.16-2.16)	80	1.69 (1.29-2.22)
394	Rheumatic disease of the heart valves	101	2.02 (1.58-2.58)	90	1.81 (1.39-2.36)	21	1.91 (1.12-3.24)	55	1.59 (1.16-2.19)	77	1.91 (1.44-2.54)
394.2	Mitral valve disease	99	1.86 (1.46-2.37)	83	1.6 (1.23-2.08)	21	1.59 (0.95-2.67)	64	1.86 (1.37-2.52)	78	1.84 (1.39-2.43)
427.1	Paroxysmal tachycardia, unspecified	89	1.69 (1.31-2.18)	85	1.49 (1.14-1.95)	22	2.11 (1.25-3.55)	61	1.7 (1.26-2.31)	80	1.78 (1.35-2.34)
415.2	Chronic pulmonary heart disease	72	1.92 (1.44-2.55)	63	1.69 (1.24-2.3)	16	2.33 (1.24-4.37)	45	1.71 (1.2-2.45)	59	1.77 (1.29-2.44)
433.8	Late effects of cerebrovascular disease	69	1.99 (1.48-2.68)	64	1.88 (1.38-2.57)	15	3.19 (1.61-6.34)	38	1.55 (1.06-2.27)	57	1.8 (1.3-2.5)
420.2	Pericarditis	75	2.82 (2.09-3.82)	67	2.54 (1.84-3.51)	16	4.21 (2.05-8.65)	45	2.3 (1.58-3.34)	66	2.91 (2.08-4.06)
401.2	Hypertensive heart and/or renal disease	55	2.37 (1.68-3.35)	50	2.09 (1.45-3.01)	8	0.98 (0.43-2.26)	38	2.14 (1.41-3.22)	45	2.27 (1.53-3.36)
394.7	Disease of tricuspid valve	39	2.52 (1.68-3.78)	34	2.26 (1.47-3.48)	7	-	18	1.74 (1-3.05)	32	2.56 (1.61-4.07)
427.8	Sinoatrial node dysfunction (Bradycardia)	38	2.75 (1.81-4.19)	36	2.68 (1.75-4.1)	9	-	23	2.44 (1.44-4.14)	33	3.11 (1.94-4.98)
**Digestive Diseases**											
530.1	Esophagitis, GERD and related diseases	639	1.27 (1.16-1.4)	617	1.24 (1.12-1.36)	166	1.46 (1.22-1.76)	459	1.26 (1.13-1.4)	545	1.22 (1.1-1.35)
550.2	Diaphragmatic hernia	507	1.32 (1.19-1.47)	470	1.26 (1.13-1.4)	115	1.38 (1.11-1.72)	354	1.3 (1.15-1.47)	440	1.31 (1.17-1.47)
563	Constipation	399	1.33 (1.18-1.49)	364	1.21 (1.07-1.36)	85	1.39 (1.08-1.79)	253	1.12 (0.97-1.29)	318	1.22 (1.07-1.39)
535	Gastritis and duodenitis	321	1.34 (1.18-1.53)	308	1.34 (1.17-1.53)	75	1.35 (1.04-1.77)	214	1.23 (1.06-1.44)	271	1.28 (1.11-1.48)
535.8	Other specified gastritis	246	1.45 (1.25-1.68)	237	1.45 (1.25-1.69)	60	1.39 (1.03-1.87)	160	1.28 (1.07-1.54)	218	1.44 (1.22-1.69)
578.9	Hemorrhage of gastrointestinal tract	207	1.45 (1.23-1.7)	197	1.41 (1.2-1.67)	53	1.85 (1.33-2.58)	131	1.3 (1.07-1.59)	174	1.39 (1.16-1.66)
558	Noninfectious gastroenteritis	215	1.57 (1.34-1.84)	206	1.49 (1.27-1.76)	56	1.83 (1.32-2.52)	153	1.49 (1.24-1.8)	175	1.47 (1.23-1.76)

785	Abdominal pain	147	1.47 (1.21-1.77)	142	1.49 (1.22-1.81)	41	1.58 (1.1-2.29)	112	1.51 (1.21-1.88)	135	1.53 (1.25-1.87)
571.5	Other chronic nonalcoholic liver disease	149	1.73 (1.42-2.1)	139	1.7 (1.39-2.08)	27	1.63 (1.04-2.58)	98	1.72 (1.36-2.19)	127	1.7 (1.37-2.11)
569	Other disorders of intestine	119	1.62 (1.3-2.01)	108	1.53 (1.22-1.92)	23	1.46 (0.89-2.39)	76	1.36 (1.04-1.78)	99	1.67 (1.31-2.13)
531.2	Gastric ulcer	109	1.71 (1.36-2.14)	97	1.54 (1.21-1.96)	29	1.88 (1.2-2.95)	76	1.66 (1.26-2.18)	96	1.7 (1.32-2.18)
550.5	Ventral hernia	83	1.66 (1.28-2.16)	83	1.72 (1.32-2.23)	13	1.24 (0.66-2.32)	63	1.79 (1.32-2.41)	69	1.69 (1.26-2.25)
575.8	Other disorders of biliary tract	63	2.08 (1.53-2.84)	56	1.78 (1.29-2.46)	9	-	37	1.74 (1.18-2.58)	53	1.92 (1.36-2.7)
557.1	Celiac disease	53	2.56 (1.8-3.64)	53	2.48 (1.74-3.55)	21	3.56 (1.96-6.49)	35	2.17 (1.43-3.3)	50	2.53 (1.75-3.64)
577	Diseases of pancreas	45	2.47 (1.69-3.61)	39	2.19 (1.47-3.26)	7	-	31	2.47 (1.57-3.9)	35	2.3 (1.5-3.53)
555.1	Regional enteritis	32	2.2 (1.42-3.41)	31	2.12 (1.35-3.32)	3	-	22	2.15 (1.27-3.64)	27	2.09 (1.3-3.39)
441	Vascular insufficiency of intestine	35	2.79 (1.78-4.37)	30	2.37 (1.48-3.78)	5	-	21	2.34 (1.33-4.13)	26	2.21 (1.33-3.67)
**Ear diseases**											
389	Hearing loss	166	1.44 (1.2-1.72)	156	1.39 (1.15-1.68)	24	1 (0.62-1.6)	103	1.3 (1.03-1.63)	138	1.45 (1.18-1.77)
**Ocular diseases**											
375	Disorders of lacrimal system	78	1.62 (1.24-2.12)	76	1.66 (1.26-2.17)	32	2.92 (1.84-4.63)	63	1.74 (1.29-2.34)	68	1.58 (1.19-2.11)
374	Other disorders of eyelids	93	2.53 (1.94-3.29)	90	2.53 (1.93-3.31)	55	6.09 (3.97-9.34)	68	2.67 (1.96-3.65)	81	2.61 (1.96-3.49)
374.3	Ptosis of eyelid	83	2.71 (2.05-3.6)	79	2.68 (2.01-3.58)	36	6.73 (3.89-11.65)	58	2.54 (1.82-3.55)	76	3.09 (2.26-4.21)
368.2	Diplopia and disorders of binocular vision	52	3.08 (2.14-4.45)	44	2.58 (1.75-3.81)	19	7.87 (3.55-17.42)	33	2.66 (1.7-4.16)	45	3.03 (2.03-4.53)
378.1	Strabismus (not specified as paralytic)	49	3.98 (2.67-5.92)	47	3.75 (2.5-5.62)	26	6.66 (3.52-12.58)	36	3.7 (2.35-5.84)	48	4.09 (2.7-6.2)
**Genitourinary Diseases**											
585.1	Acute renal failure	440	1.79 (1.59-2.01)	383	1.55 (1.37-1.75)	81	1.62 (1.24-2.11)	271	1.58 (1.37-1.83)	332	1.64 (1.44-1.88)
585.3	Chronic renal failure [CKD]	402	1.7 (1.51-1.92)	385	1.65 (1.46-1.87)	85	1.91 (1.46-2.49)	238	1.46 (1.26-1.7)	330	1.67 (1.46-1.92)
580.2	Nephrotic syndrome without mention of glomerulonephritis	30	2.63 (1.62-4.26)	30	2.52 (1.55-4.1)	9	-	20	2.44 (1.37-4.36)	28	2.83 (1.69-4.75)
**Hematologic Diseases**											
285	Other anemias	434	1.79 (1.59-2.01)	388	1.62 (1.43-1.83)	91	1.77 (1.37-2.29)	273	1.53 (1.32-1.76)	352	1.66 (1.46-1.9)
280.1	Iron deficiency anemias	364	1.63 (1.44-1.84)	339	1.55 (1.36-1.76)	77	1.65 (1.25-2.17)	229	1.41 (1.21-1.64)	300	1.52 (1.32-1.74)
288.11	Neutropenia	91	1.61 (1.26-2.06)	70	1.23 (0.94-1.62)	25	2.61 (1.57-4.34)	63	1.54 (1.15-2.06)	49	1.01 (0.74-1.4)
287.3	Thrombocytopenia	78	2.06 (1.56-2.72)	67	1.83 (1.37-2.46)	13	1.72 (0.89-3.33)	45	1.76 (1.23-2.51)	48	1.57 (1.12-2.22)
281.1	Megaloblastic anemia	54	1.88 (1.35-2.61)	53	1.73 (1.24-2.42)	10	1.79 (0.84-3.81)	28	1.54 (0.99-2.4)	46	1.97 (1.36-2.83)
285.2	Anemia of chronic disease	39	2.25 (1.51-3.36)	38	2.25 (1.49-3.38)	8	-	25	2.26 (1.38-3.71)	30	2.43 (1.51-3.89)
**Infectious Diseases**											
591	Urinary tract infection	429	1.77 (1.58-1.99)	385	1.61 (1.42-1.82)	104	2.1 (1.65-2.68)	268	1.57 (1.36-1.81)	339	1.63 (1.43-1.86)
8	Intestinal infection	358	1.66 (1.46-1.88)	319	1.53 (1.34-1.74)	74	1.7 (1.29-2.24)	224	1.49 (1.28-1.75)	287	1.59 (1.38-1.84)
480.1	Bacterial pneumonia	323	1.61 (1.41-1.84)	289	1.48 (1.29-1.7)	69	1.64 (1.23-2.19)	221	1.59 (1.36-1.87)	253	1.49 (1.28-1.73)
480	Pneumonia	277	1.71 (1.48-1.97)	251	1.58 (1.36-1.84)	61	1.83 (1.34-2.49)	177	1.5 (1.25-1.79)	215	1.6 (1.36-1.89)
994.2	Sepsis	234	1.86 (1.58-2.17)	193	1.55 (1.31-1.84)	39	1.54 (1.06-2.24)	145	1.66 (1.36-2.02)	172	1.65 (1.37-1.98)
41	Bacterial infection NOS	150	1.63 (1.35-1.99)	127	1.41 (1.14-1.74)	28	1.46 (0.94-2.26)	97	1.49 (1.17-1.89)	120	1.58 (1.27-1.97)
41.4	E. coli	168	1.93 (1.6-2.33)	152	1.77 (1.46-2.16)	38	1.92 (1.3-2.85)	104	1.73 (1.37-2.19)	124	1.72 (1.38-2.14)
79	Viral infection	115	1.54 (1.23-1.92)	104	1.39 (1.1-1.75)	22	1.55 (0.93-2.57)	67	1.31 (0.99-1.74)	86	1.36 (1.06-1.76)

112	Candidiasis	124	1.74 (1.4-2.15)	103	1.45 (1.15-1.83)	23	1.63 (1-2.68)	69	1.43 (1.08-1.89)	87	1.45 (1.13-1.87)
41.2	Streptococcus infection	67	1.76 (1.32-2.36)	56	1.56 (1.14-2.13)	14	2.05 (1.07-3.94)	40	1.35 (0.94-1.94)	53	1.62 (1.16-2.26)
136	Other infectious and parasitic diseases	36	2.8 (1.81-4.33)	31	2.43 (1.54-3.83)	9	-	25	2.61 (1.56-4.36)	32	2.36 (1.49-3.74)
481	Influenza	26	3 (1.79-5.05)	20	2.5 (1.4-4.47)	2	-	13	2.23 (1.11-4.47)	21	3.43 (1.85-6.35)
**Metabolic Diseases**											
244	Hypothyroid conditions	1,516	14.79 (13.18-16.59)	1429	13.14 (11.73-14.73)	451	16.7 (13.39-20.83)	1,443	14.68 (13.05-16.51)	1355	14.55 (12.84-16.48)
250.2	Type 2 diabetes	413	1.3 (1.16-1.46)	405	1.29 (1.15-1.45)	106	1.7 (1.35-2.15)	303	1.36 (1.19-1.55)	356	1.24 (1.09-1.4)
276.1	Electrolyte imbalance	362	1.64 (1.44-1.85)	320	1.42 (1.25-1.63)	78	1.85 (1.4-2.43)	227	1.51 (1.29-1.77)	275	1.5 (1.3-1.74)
276.5	Hypovolemia	224	1.79 (1.52-2.1)	206	1.59 (1.34-1.89)	39	1.5 (1.03-2.18)	133	1.46 (1.19-1.79)	163	1.58 (1.31-1.91)
261.4	Vitamin D deficiency	143	1.77 (1.45-2.17)	127	1.57 (1.27-1.94)	29	1.85 (1.18-2.9)	99	1.85 (1.45-2.36)	107	1.59 (1.26-2.01)
275.5	Disorders of calcium/phosphorus metabolism	137	2 (1.62-2.46)	123	1.77 (1.42-2.21)	28	2.46 (1.52-3.98)	88	1.75 (1.36-2.26)	96	1.64 (1.28-2.1)
276.4	Acid-base balance disorder	92	1.68 (1.31-2.16)	82	1.52 (1.16-1.98)	15	1.34 (0.74-2.44)	49	1.44 (1.03-2)	66	1.45 (1.08-1.95)
275.3	Disorders of magnesium metabolism	101	2.38 (1.84-3.06)	83	1.92 (1.46-2.53)	20	2.82 (1.55-5.13)	52	1.8 (1.28-2.53)	72	2.02 (1.5-2.72)
261.2	Vitamin B-complex deficiencies	86	2.08 (1.59-2.72)	80	1.99 (1.51-2.62)	20	2.39 (1.36-4.2)	52	1.83 (1.31-2.56)	73	1.91 (1.43-2.56)
276.6	Fluid overload	98	2.79 (2.14-3.64)	80	2.03 (1.54-2.7)	17	2.3 (1.25-4.23)	54	2.02 (1.45-2.83)	75	2.14 (1.59-2.89)
251.1	Hypoglycemia	84	2.12 (1.61-2.78)	86	2.48 (1.88-3.29)	14	1.75 (0.92-3.31)	61	2.47 (1.78-3.43)	67	2.22 (1.62-3.03)
246	Other disorders of thyroid	142	8.64 (6.39-11.67)	127	6.95 (5.17-9.34)	44	12.84 (6.91-23.85)	101	8.22 (5.8-11.66)	121	8.09 (5.82-11.25)
252.1	Hyperparathyroidism	110	4.68 (3.53-6.2)	95	4.26 (3.16-5.74)	24	4.43 (2.46-7.96)	88	4.82 (3.52-6.61)	88	4.4 (3.2-6.06)
250.1	Type 1 diabetes	56	2.02 (1.45-2.8)	53	1.92 (1.36-2.69)	16	3.38 (1.72-6.63)	40	2.15 (1.45-3.18)	50	1.94 (1.36-2.75)
241.2	Nontoxic multinodular goiter	68	8.24 (5.39-12.62)	52	4.82 (3.2-7.26)	18	9.85 (4.11-23.6)	53	8.73 (5.34-14.25)	54	8.13 (4.98-13.28)
240	Simple and unspecified goiter	54	6.68 (4.27-10.47)	47	4.55 (2.94-7.05)	11	4.15 (1.76-9.8)	48	7.65 (4.64-12.62)	50	7.42 (4.55-12.1)
241.1	Nontoxic uninodular goiter	32	2.74 (1.73-4.34)	29	2.52 (1.57-4.04)	7	-	28	3.47 (2.07-5.81)	26	2.52 (1.51-4.19)
252.2	Hypoparathyroidism	51	9.61 (5.72-16.13)	47	7.99 (4.81-13.3)	11	14.12 (3.93-50.65)	43	13.42 (7.07-25.48)	41	7.98 (4.57-13.92)
227.2	Benign neoplasm of parathyroid gland	36	4.26 (2.64-6.86)	23	2.53 (1.47-4.34)	5	-	30	5.16 (2.97-8.95)	30	3.81 (2.27-6.38)
255.21	Glucocorticoid deficiency	30	4.55 (2.69-7.69)	23	3.32 (1.86-5.93)	6	-	17	4.18 (2.11-8.27)	25	5.38 (2.9-9.98)
255	Disorders of adrenal glands	23	2.96 (1.68-5.2)	23	2.86 (1.6-5.14)	7	-	16	2.6 (1.34-5.06)	18	2.43 (1.29-4.56)
193	Thyroid cancer	21	4.21 (2.26-7.83)	15	2.31 (1.22-4.39)	7	-	16	3.64 (1.84-7.2)	18	4.27 (2.11-8.64)
245.2	Chronic thyroiditis	20	4.99 (2.55-9.76)	30	22.05 (8.51-57.11)	7	-	24	15.15 (6.18-37.11)	25	14.29 (5.85-34.92)
253.1	Pituitary hyperfunction	32	20.45 (8.54-48.94)	26	14.5 (6.29-33.42)	6	-	22	21.12 (7.27-61.34)	20	11.34 (4.54-28.3)
245	Thyroiditis	30	18.99 (7.9-45.66)	16	2.84 (1.49-5.42)	7	-	15	5.1 (2.33-11.12)	19	5.84 (2.77-12.32)
226	Benign neoplasm of thyroid glands	16	5.82 (2.7-12.54)	13	4.33 (1.98-9.5)	5	-	15	6 (2.7-13.36)	14	6.47 (2.71-15.46)
**Musculoskeletal And Joint Diseases**											
743.11	Osteoporosis NOS	322	1.39 (1.22-1.58)	294	1.28 (1.11-1.46)	52	0.98 (0.72-1.33)	198	1.19 (1.01-1.39)	260	1.26 (1.09-1.46)
760	Back pain	311	1.32 (1.15-1.5)	312	1.36 (1.19-1.56)	86	1.96 (1.51-2.55)	214	1.33 (1.13-1.56)	269	1.39 (1.2-1.6)
721	Spondylosis and allied disorders	147	1.49 (1.23-1.8)	141	1.43 (1.17-1.74)	35	1.98 (1.32-2.98)	93	1.29 (1.02-1.63)	119	1.49 (1.2-1.85)
**Psychiatric And Behavioral Diseases**											
300.1	Anxiety disorder	363	1.38 (1.22-1.55)	344	1.34 (1.18-1.51)	99	1.95 (1.53-2.49)	242	1.36 (1.17-1.58)	308	1.37 (1.2-1.57)
290.2	Delirium due to conditions classified elsewhere	154	1.76 (1.45-2.14)	134	1.58 (1.28-1.94)	26	1.56 (0.98-2.47)	89	1.44 (1.12-1.84)	120	1.63 (1.31-2.04)
**Respiratory Diseases**											

496	Chronic airway obstruction	291	1.4 (1.23-1.61)	281	1.41 (1.23-1.63)	65	1.62 (1.21-2.18)	195	1.3 (1.1-1.53)	237	1.36 (1.17-1.58)
507	Pleurisy; pleural effusion	278	1.56 (1.35-1.8)	243	1.35 (1.16-1.57)	59	1.48 (1.09-2.01)	168	1.29 (1.08-1.55)	206	1.4 (1.19-1.65)
519.8	Other diseases of respiratory system, NEC	236	1.51 (1.3-1.76)	217	1.4 (1.19-1.64)	47	1.37 (0.98-1.92)	151	1.36 (1.13-1.64)	191	1.5 (1.27-1.78)
508	Pulmonary collapse; interstitial and compensatory emphysema	169	1.69 (1.4-2.03)	153	1.53 (1.26-1.86)	29	1.34 (0.87-2.07)	101	1.41 (1.12-1.78)	125	1.49 (1.21-1.85)
509.1	Respiratory failure	168	1.69 (1.41-2.03)	149	1.52 (1.25-1.85)	24	1.13 (0.71-1.8)	111	1.59 (1.27-1.99)	136	1.66 (1.35-2.04)
496.2	Chronic bronchitis	160	1.61 (1.33-1.94)	147	1.58 (1.3-1.92)	31	1.49 (0.97-2.28)	106	1.54 (1.23-1.94)	134	1.62 (1.32-2)
327.3	Sleep apnea	129	1.67 (1.36-2.06)	127	1.67 (1.35-2.06)	28	1.83 (1.17-2.88)	87	1.55 (1.21-1.99)	113	1.6 (1.28-2)
479	Other upper respiratory disease	69	1.88 (1.4-2.51)	59	1.63 (1.2-2.23)	18	2.28 (1.27-4.1)	50	1.87 (1.33-2.63)	57	1.68 (1.22-2.31)
501	Pneumonitis due to inhalation of food or vomitus	67	1.95 (1.44-2.64)	55	1.61 (1.16-2.24)	13	2.62 (1.28-5.36)	45	1.82 (1.27-2.63)	50	1.67 (1.17-2.37)
**Skin Diseases**											
707.1	Decubitus ulcer	117	1.93 (1.54-2.42)	102	1.71 (1.35-2.18)	20	2.26 (1.29-3.99)	68	1.58 (1.19-2.1)	86	1.66 (1.28-2.17)
681.7	Cellulitis and abscess of trunk	30	3.49 (2.12-5.75)	28	3.64 (2.16-6.12)	8	-	15	2.58 (1.34-4.98)	25	3.81 (2.14-6.79)

HT = Hyperthyroidism; HR = Hazard ratio; CI = Confidence interval.

- = The model was unable to accurately estimate the effect due to an insufficient sample size.

Table S12. Sensitivity analyses to investigate the association between prior diagnosed hyperthyroidism and death causes with different restrictions.

Cause of death	Overall (N = 28,411, HT cases = 5,832)			Subgroup with at least two recorded diagnoses of hyperthyroidism (N = 4,724, HT cases = 961)			Subgroup with no history of other thyroid diseases prior to the index date (N = 19,299, HT cases = 3,952)			Subgroup with no history of malignancy diagnosis prior to the index date (N = 21,540, HT cases = 4,811)		
	N (HT population)	HR (95% CI)	P-value	N (HT population)	HR (95% CI)	P-value	N (HT population)	HR (95% CI)	P-value	N (HT population)	HR (95% CI)	P-value
All-cause mortality	766	1.87 (1.71-2.04)	4.65E-45	120	1.54 (1.25-1.91)	7.10E-05	483	1.69 (1.52-1.88)	1.70E-21	511	1.54 (1.38-1.71)	1.39E-15
Malignant neoplasms death	319	1.49 (1.3-1.7)	5.20E-09	60	1.69 (1.25-2.29)	6.40E-04	208	1.4 (1.19-1.65)	4.90E-05	148	0.92 (0.77-1.11)	4.10E-01
Cardiovascular disease death	162	1.82 (1.49-2.21)	3.10E-09	22	1.52 (0.9-2.56)	1.20E-01	102	1.68 (1.31-2.15)	3.80E-05	139	2.02 (1.62-2.51)	3.20E-10
Respiratory system disease death	79	1.76 (1.33-2.35)	9.50E-05	16	1.75 (0.91-3.39)	9.50E-02	52	1.87 (1.31-2.66)	5.50E-04	64	1.88 (1.36-2.59)	1.20E-04
Neural system disease death	43	1.11 (0.77-1.59)	5.80E-01	8	-	-	28	1.13 (0.72-1.78)	5.90E-01	38	1.11 (0.76-1.64)	5.80E-01
Digestive system disease death	40	2.03 (1.36-3.04)	5.20E-04	4	-	-	23	1.75 (1.03-2.96)	3.80E-02	26	2.08 (1.26-3.42)	3.90E-03
Mental disorder death	26	1.74 (1.06-2.84)	2.80E-02	1	-	-	15	1.68 (0.89-3.18)	1.10E-01	19	1.7 (0.96-3)	6.70E-02

HT = Hyperthyroidism; HR = Hazard ratio; CI = Confidence interval.

- = The model was unable to accurately estimate the effect due to an insufficient sample size.

Stratified Cox regression was performed to examine the association between hyperthyroidism and all-cause mortality. The Fine-Gray competing risk model was used to assess the impact of hyperthyroidism on specific causes of death.

All p-value reported has been adjusted using Bonferroni correction.

Supplementary References

1. Bassett DR. International physical activity questionnaire: 12-country reliability and validity. Med Sci Sports Exerc. 2003;35(8):1396.
2. Naser JA, Pislaru SV, Stan MN, Lin G. Incidence, Risk Factors, and Outcomes of Incident Atrial Fibrillation in Patients With Graves Disease. Mayo Clin Proc. 2023;98(6):883-891.
3. Bekiaridou A, Kartas A, Moysidis DV, Papazoglou AS, Baroutidou A, Papanastasiou A, et al. The bidirectional relationship of thyroid disease and atrial fibrillation: Established knowledge and future considerations. Rev Endocr Metab Disord. 2022;23(3):621-630.
4. Dekkers OM, Horváth-Puhó E, Cannegieter SC, Vandenbroucke JP, Sørensen HT, Jørgensen JOL. Acute cardiovascular events and all-cause mortality in patients with hyperthyroidism: a population-based cohort study. Eur J Endocrinol. 2017;176(1):1-9.
5. Sohn SY, Lee E, Lee MK, Lee JH. The Association of Overt and Subclinical Hyperthyroidism with the Risk of Cardiovascular Events and Cardiovascular Mortality: Meta-Analysis and Systematic Review of Cohort Studies. Endocrinol Metab (Seoul). 2020;35(4):786-800.
6. Kim HJ, Kang T, Kang MJ, Ahn HS, Sohn SY. Incidence and Mortality of Myocardial Infarction and Stroke in Patients with Hyperthyroidism: A Nationwide Cohort Study in Korea. Thyroid. 2020;30(7):955-965.
7. Corona G, Croce L, Sparano C, Petrone L, Sforza A, Maggi M, et al. Thyroid and heart, a clinically relevant relationship. J Endocrinol Invest. 2021;44(12):2535-2544.
8. Liu J, Wu G, Li S, Cheng L, Ye X. The genetic association between hyperthyroidism and heart failure: a Mendelian randomization study. Front Endocrinol (Lausanne). 2024;15:1344282.
9. Biondi B, Kahaly GJ. Cardiovascular involvement in patients with different causes of hyperthyroidism. Nat Rev Endocrinol. 2010;6(8):431-443.
10. Squizzato A, Gerdes VEA, Brandjes DPM, Büller HR, Stam J. Thyroid diseases and cerebrovascular disease. Stroke. 2005;36(10):2302-2310.
11. Channick BJ, Adlin EV, Marks AD, Denenberg BS, McDonough MT, Chakko CS, et al. Hyperthyroidism and mitral-valve prolapse. N Engl J Med. 1981;305(9):497-500.
12. Ata F, Khan HA, Choudry H, Khan AA, Tahir S, Cerqueira TL, et al. A systematic review of the clinical characteristics and course of atrioventricular blocks in hyperthyroidism. Ann Med. 2024;56(1):2365405.
13. Stuijver DJF, van Zaane B, Romualdi E, Brandjes DPM, Gerdes VEA, Squizzato A. The effect of hyperthyroidism on procoagulant, anticoagulant and fibrinolytic factors: a systematic review and meta-analysis. Thromb Haemost. 2012;108(6):1077-1088.
14. Araruna LVM, de Oliveira DC, Pereira MC, Moura Neto A, Tambascia MA, Zantut-Wittmann DE. Interplay Between Thyroid Hormone Status and Pulmonary Hypertension in Graves' Disease: Relevance of the Assessment in Thyrotoxic and Euthyroid Patients. Front Endocrinol (Lausanne). 2021;12:780397.
15. Robinson K, Menon S, Rungvivatjarus T. Symptomatic Pericardial Effusion Associated With Graves' Disease in a Pediatric Patient. Pediatrics. 2024;153(6):e2023064782.
16. Gupta P, Chhabra L, Hiendlmayr B, Spodick DH. Thyrotoxic pericarditis: An underappreciated phenomenon. Int J Cardiol. 2015;198:32-33.
17. Inami T, Seino Y, Goda H, Okazaki H, Shirakabe A, Yamamoto M, et al. Acute pericarditis: unique comorbidity of thyrotoxic crisis with Graves' disease. Int J Cardiol. 2014;171(3):e129-130.
18. Berta E, Lengyel I, Halmi S, Zrínyi M, Erdei A, Harangi M, et al. Hypertension in Thyroid Disorders. Front Endocrinol (Lausanne). 2019;10:482.
19. Xu GM, Hu MX, Li SY, Ran X, Zhang H, Ding XF. Thyroid disorders and gastrointestinal dysmotility: an old association. Front Physiol. 2024;15:1389113.
20. Ferrari SM, Fallahi P, Ruffilli I, Elia G, Ragusa F, Benvenga S, et al. The association of other autoimmune diseases in patients with Graves' disease (with or without ophthalmopathy): Review of the literature and report of a large series. Autoimmun Rev. 2019;18(3):287-292.
21. Tozzoli R, Kodermaz G, Perosa AR, Tampoia M, Zucano A, Antico A, et al. Autoantibodies to parietal cells as predictors of atrophic body gastritis: a five-year prospective study in patients with autoimmune thyroid diseases. Autoimmun Rev. 2010;10(2):80-83.
22. Daher R, Yazbeck T, Jaoude JB, Abboud B. Consequences of dysthyroidism on the digestive tract and viscera. World J Gastroenterol. 2009;15(23):2834-2838.
23. Nuovo JA, Baker JR, Wartofsky L, Lukes YG, Burman KD. Autoantibodies to insulin are present in sera of patients with autoimmune thyroid disease. Diabetes. 1988;37(3):317-320.
24. Xian W, Wu D, Liu B, Hong S, Huo Z, Xiao H, et al. Graves Disease and Inflammatory Bowel Disease: A Bidirectional Mendelian Randomization. J Clin Endocrinol Metab. 2023;108(5):1075-1083.
25. Bonapace ES, Srinivasan R. Simultaneous occurrence of inflammatory bowel disease and thyroid disease. Am J Gastroenterol. 2001;96(6):1925-1926.
26. Wang TC, Chiu CJ, Chen PC, Chang TY, Tyler RS, Rojas-Roncancio E, et al. Increased Incidence of Tinnitus Following a Hyperthyroidism Diagnosis: A Population-Based Longitudinal Study. Front Endocrinol (Lausanne). 2021;12:741719.
27. Aydogan F, Ayhan Tuzcu E, Aydogan A, Akkucuk S, Coskun M, Ustun I, et al. Effect of radioactive iodine therapy on lacrimal gland functions in patients with hyperthyroidism. Clin Nucl Med. 2014;39(4):315-318.
28. Gürez C, Ergül N. Does Radioactive Iodine Treatment Damage the Lacrimal System? Clin Nucl Med. 2024;49(6):513-515.
29. Leonard TJ, Graham EM, Stanford MR, Sanders MD. Graves' disease presenting with bilateral acute painful proptosis, ptosis, ophthalmoplegia, and visual loss. Lancet. 1984;2(8400):431-433.
30. You AS, Kalantar-Zadeh K, Brent GA, Narasaki Y, Daza A, Sim JJ, et al. Impact of Thyroid Status on Incident Kidney Dysfunction and Chronic Kidney Disease Progression in a Nationally Representative Cohort. Mayo Clin Proc. 2024;99(1):39-56.
31. Gopinath B, Harris DC, Wall JR, Kifley A, Mitchell P. Relationship between thyroid dysfunction and chronic kidney disease in community-dwelling older adults. Maturitas. 2013;75(2):159-164.
32. Neves PDM de M, Muniz MPR, Morgantetti GF, Cutrim ÉMM, Macieira C de A, Salgado-Filho N, et al. Membranous Nephropathy Secondary to Graves' Disease: A Case Report. Front Immunol. 2022;13:824124.
33. M'Rabet-Bensalah K, Aubert CE, Coslovsky M, Collet TH, Baumgartner C, den Elzen WPJ, et al. Thyroid dysfunction and anaemia in a large population-based study. Clin Endocrinol (Oxf). 2016;84(4):627-631.
34. Szczepanek-Parulska E, Hernik A, Ruchała M. Anemia in thyroid diseases. Pol Arch Intern Med. 2017;127(5):352-360.
35. Hymes K, Blum M, Lackner H, Karpatkin S. Easy bruising, thrombocytopenia, and elevated platelet immunoglobulin G in Graves' disease and Hashimoto's thyroiditis. Ann Intern Med. 1981;94(1):27-30.
36. Conrad N, Misra S, Verbakel JY, Verbeke G, Molenberghs G, Taylor PN, et al. Incidence, prevalence, and co-occurrence of autoimmune disorders over time and by age, sex, and socioeconomic status: a population-based cohort study of 22 million individuals in the UK. Lancet. 2023;401(10391):1878-1890.
37. Hong J, Zhang L, Lai Y, Chen X, Chen Y, Yang J. Causal association between thyroid dysfunction and sepsis: a two-sample mendelian randomization study. Front Endocrinol (Lausanne). 2024;15:1348248.
38. Moroto D, Torquato-Vieira ICO, Fiorin LB, Camacho CP, Castiglioni MLV, Maciel RMB, et al. Long-Term Follow-up of Patients With Autonomous Thyroid Nodules Treated With Radioiodine. Clin Nucl Med. 2024;49(6):529-535.

39. Metso S, Jaatinen P, Huhtala H, Luukkaala T, Oksala H, Salmi J. Long-term follow-up study of radioiodine treatment of hyperthyroidism. Clin Endocrinol (Oxf). 2004;61(5):641-648.
40. Popoviciu MS, Paduraru L, Nutas RM, Ujoc AM, Yahya G, Metwally K, et al. Diabetes Mellitus Secondary to Endocrine Diseases: An Update of Diagnostic and Treatment Particularities. Int J Mol Sci. 2023;24(16):12676.
41. Song E, Koo MJ, Noh E, Hwang SY, Park MJ, Kim JA, et al. Risk of Diabetes in Patients with Long-Standing Graves' Disease: A Longitudinal Study. Endocrinol Metab (Seoul). 2021;36(6):1277-1286.
42. Pompeo A, Nepa A, Maddestra M, Feliziani V, Genovesi N. Thyrotoxic hypokalemic periodic paralysis: An overlooked pathology in western countries. Eur J Intern Med. 2007;18(5):380-390.
43. Vieira IH, Rodrigues D, Paiva I. Vitamin D and Autoimmune Thyroid Disease-Cause, Consequence, or a Vicious Cycle? Nutrients. 2020;12(9):2791.
44. Popelier M, Jollivet B, Fouquet B, Lasfargues G, Valat C, Valat JP, et al. [Phosphorus-calcium metabolism in hyperthyroidism]. Presse Med. 1990;19(15):705-708.
45. Kimmoun A, Abboud G, Strazeck J, Merten M, Guéant JL, Feillet F. Acute decompensation of isovaleric acidemia induced by Graves' disease. Intensive Care Med. 2008;34(12):2315-2316.
46. Dolev E, Deuster PA, Solomon B, Trostmann UH, Wartofsky L, Burman KD. Alterations in magnesium and zinc metabolism in thyroid disease. Metabolism. 1988;37(1):61-67.
47. Benites-Zapata VA, Ignacio-Cconchoy FL, Ulloque-Badaracco JR, Hernandez-Bustamante EA, Alarcón-Braga EA, Al-Kassab-Córdova A, et al. Vitamin B12 levels in thyroid disorders: A systematic review and meta-analysis. Front Endocrinol (Lausanne). 2023;14:1070592.
48. Bondeson AG, Bondeson L, Thompson NW. Hyperparathyroidism after treatment with radioactive iodine: not only a coincidence? Surgery. 1989;106(6):1025-1027.
49. Wang J, Wan K, Chang X, Mao RF. Association of autoimmune thyroid disease with type 1 diabetes mellitus and its ultrasonic diagnosis and management. World J Diabetes. 2024;15(3):348-360.
50. Silva JE, Bianco SDC. Thyroid-adrenergic interactions: physiological and clinical implications. Thyroid. 2008;18(2):157-165.
51. Yuan S, Kar S, Vithayathil M, Carter P, Mason AM, Burgess S, et al. Causal associations of thyroid function and dysfunction with overall, breast and thyroid cancer: A two-sample Mendelian randomization study. Int J Cancer. 2020;147(7):1895-1903.
52. Kitahara CM, K Rmendiné Farkas D, Jørgensen JOL, Cronin-Fenton D, Sørensen HT. Benign Thyroid Diseases and Risk of Thyroid Cancer: A Nationwide Cohort Study. J Clin Endocrinol Metab. 2018;103(6):2216-2224.
53. Tran TVT, Kitahara CM, de Vathaire F, Boutron-Ruault MC, Journy N. Thyroid dysfunction and cancer incidence: a systematic review and meta-analysis. Endocr Relat Cancer. 2020;27(4):245-259.
54. Qi W, Wang D, Hong Y, Yao J, Wang H, Zhu L, et al. Investigating the causal relationship between thyroid dysfunction diseases and osteoporosis: a two-sample Mendelian randomization analysis. Sci Rep. 2024;14(1):12784.
55. Rosen CJ, Adler RA. Longitudinal changes in lumbar bone density among thyrotoxic patients after attainment of euthyroidism. J Clin Endocrinol Metab. 1992;75(6):1531-1534.
56. Soheili-Nezhad S, Sprooten E, Tendolkar I, Medici M. Exploring the Genetic Link Between Thyroid Dysfunction and Common Psychiatric Disorders: A Specific Hormonal or a General Autoimmune Comorbidity. Thyroid. 2023;33(2):159-168.
57. Brandt F, Thvilum M, Almind D, Christensen K, Green A, Hegedüs L, et al. Hyperthyroidism and psychiatric morbidity: evidence from a Danish nationwide register study. Eur J Endocrinol. 2014;170(2):341-348.
58. Holmberg M, Malmgren H, Berglund PF, Johansson B, Nyström HF. Psychiatric complications in Graves' disease. Eur Thyroid J. 2024;13(1):e230247.
59. Zader SJ, Williams E, Buryk MA. Mental Health Conditions and Hyperthyroidism. Pediatrics. 2019;144(5):e20182874.
60. Goldfarb CR, Varma C, Roginsky MS. Diagnosis in delirium: prompt confirmation of thyroid storm. Clin Nucl Med. 1980;5(2):66.
61. Brüssel T, Matthay MA, Chernow B. Pulmonary manifestations of endocrine and metabolic disorders. Clin Chest Med. 1989;10(4):645-653.
62. Li Pi Shan W, Hatzakorzian R, Sherman M, Backman SB. Upper airway compromise secondary to edema in Graves' disease. Can J Anaesth. 2006;53(2):183-187.
